# Effects of transthoracic device closure on ventricular septal defects and reasons for conversion to open-heart surgery: A meta-analysis

**DOI:** 10.1038/s41598-017-12500-6

**Published:** 2017-09-22

**Authors:** Yang Zhou, Ling-Xi Liu, Fei Zhao, Shi-Hai Tang, Hua-Li Peng, Yun-Han Jiang

**Affiliations:** 1 Department of Cardiothoracic Surgery, the People’s Hospital of Leshan, Leshan, Sichuan Province 614000 P.R. China; 2 Department of Cardiovascular Surgery, Xinqiao Hospital, Third Military Medical University, Chongqing, Chongqing 400030 P.R. China

## Abstract

Transthoracic device closure (TTDC) is thought to be a promising technology for the repair of ventricular septal defects (VSDs). However, there is considerable controversy regarding the efficacy and safety of TTDC. The present study aimed to compare the benefits and safety of TTDC with those of conventional open-heart surgery (COHS) and analyze the associated factors causing complications, conversion to COHS and reoperation. Electronic database searches were conducted in PubMed, EMBASE, Cochrane Library, Clinicaltrials.gov and several Chinese databases. A total of 5 randomized controlled trials (RCTs), 7 cohort studies, 13 case-control studies, 129 case series and 13 case reports were included. Compared to COHS, TTDC exhibited superior efficacy with a significantly lower risk of post-operative arrhythmia; however, no significant differences in other outcomes were identified. Meta-regression analysis showed that perimembranous VSDs (pmVSDs), a smaller VSD, a smaller occluder, and a median or subxiphoid approach lowered the relative risk of several post-operative complications, conversion to COHS and reoperation. The current evidence indicates that TTDC is associated with a lower risk of post-operative arrhythmia and is not associated with an increased risk of complications. PmVSDs, a smaller VSD and occluder, and a median or subxiphoid approach correlate with better outcomes when using TTDC.

## Introduction

Ventricular septal defects (VSDs) are one of the most common congenital heart diseases, accounting for approximately 20–40% of all cardiac malformations^1,2^. Closure via conventional open-heart surgery (COHS) is the gold standard of therapy for patients with VSDs^3^. Although this technology can help surgeons to directly and reliably address multiple malformations, cardiopulmonary bypass and median sternotomy can cause severe mental and physical harm to patients with VSDs. Given these limitations associated with COHS, transcatheter device closure has become widely accepted as a therapeutic alternative for patients with VSD. However, in younger infants, the relatively large sheaths used for device delivery cannot pass through the very small femoral or radial artery. In addition, the percutaneous approach can be problematic due to its vascular-related complications. Based on these factors, transthoracic device closure (TTDC), a new hybrid technology combining percutaneous occlusion and open-heart surgery closure, has been developed. This novel approach can be used to avoid not only cardiopulmonary bypass and median sternotomy, but also vascular injuries caused by interventional closure.

Although several experts have compared the efficacy of TTDC and COHS, the matter remains controversial. Xing *et al*.^4^ reported fewer complications with TTDC than with COHS, whereas Zhang and colleagues^5^ found no significant differences between these approaches in terms of intra- and post-operative complications. Moreover, several investigators have reported a higher morbidity of an intra-operative residual shunt in the TTDC group than that in COHS^6^. Another unavoidable matter is the factors that affect the conversion from TTDC to COHS. However, few papers have summarized and analyzed this topic. Therefore, the objective of this systematic review was to compare the effectiveness of TTDC and COHS for the repair of VSDs and to comprehensively analyze the reasons for conversion to COHS.

## Methods

The systematic literature review and meta-analysis were performed in accordance with the recommendations of the Preferred Reporting Items for Systematic Reviews and Meta-Analyses (PRISMA) guidelines (Appendix 1)^7,8^.

### Search Strategy

The following databases were systematically searched through December 31, 2016 to identify trials that compared the uses of TTDC and COHS in VSD patients: PubMed (MEDLINE), Embase, the Cochrane Library, WANGFANG, VIP Database for China Science and Technology Journal (VIP), Chinese National Knowledge Infrastructure (CNKI), Chinese Biomedical and Medical Database (CBM), the Chinese Clinical Trial Registry (available at: http://www.chictr.org/) and the international clinical trial registry of the U.S. National Institutes of Health (available at: http://clinicaltrials.gov/). We conducted the search strategy using the following terms: (“Heart Septal Defects, Ventricular”[MeSH Terms]or “Ventricular Septal Defect*“[All Fields] or “Intraventricular Septal Defect*“[All Fields]) and (((“Minimally Invasive Surgical Procedures”[MeSH Terms] or “Minimally Invasive Surgical Procedures”[All Fields] or “Minimal Access Surgical Procedures”[All Fields] or “Minimal Surgical Procedure”[All Fields] or “Minimally Invasive Surgery”[All Fields] or “Minimally Invasive Surgeries”[All Fields] or “Minimal Surgical Procedures”[All Fields]) and Transthoracic[All Fields]) OR (Transthoracic[All Fields] AND Minimally[All Fields] AND Invasive[All Fields] AND (“equipment and supplies”[MeSH Terms] OR (“equipment”[All Fields] AND “supplies”[All Fields]) OR “equipment and supplies”[All Fields] OR “device”[All Fields]) AND Closure[All Fields])). We did not apply language limitations. References from the relevant reviews and original papers were also searched to identify potential trials.

### Study Selection

Studies were included if they fulfilled the following inclusion criteria: 1) randomized controlled trials (RCTs), prospective cohort studies or retrospective case-control studies that evaluated the efficacy of TTDC and COHS in VSD patients, or case series that described the use of TTDC in VSD patients; 2) the success rate as the outcome, along with detailed reports of the intra- and post-operative complications of TTDC and COHS; and 3) studies published as full articles or meeting abstracts with full data, theses, or case reports. Studies were excluded if they failed to meet the above-described criteria or provided incomplete or irretrievable data. If data were reported more than once, the study with the longest follow-up time was included.

Two independent authors (Yang Zhou and Shi-Hai Tang) screened the search results for relevant articles that met the inclusion and exclusion criteria. All disputes were resolved through consensus. If a consensus could not be reached, another author (Yun-Han Jiang) was asked to make a final decision.

### Data Extraction and Quality Assessment

Data were systematically extracted from the studies and independently compiled by two reviewers (Ling-Xi Liu and Fei Zhao) using a standardized electronic sheet and were cross-checked to reach a consensus. In cases of disagreement, a consensus was reached by discussion. Trial and patient characteristics were recorded, including the name of the first author, year of publication, country, case/control, study population, mean age or range, gender, weight, types of echocardiography, operative approach, type of occluder, and size and type of VSD. The outcomes of interest were the success rate, defined as the proportion of patients with no residual shunts and no need for conversion to COHS, and morbidity from intra- and post-operative complications, including arrhythmia, valvular regurgitation, residual shunts and others. The other events were clinical indicators, including the duration of the procedure, intensive care unit stay, hospital stay, total medical costs, and the number of transfused patients. VSDs were mainly classified according to the Anderson method^9,10^, and included muscular VSD (mVSD), perimembranous VSD (pmVSD), doubly committed subarterial VSD (dcsVSD) and the special type of VSD. The latter was defined as the coexistence of multiple types of VSDs, including a patient with both a mVSD and a pmVSD^11^ or an acquired VSD, such as a post-myocardial infarction VSD (pi-VSD)^12^ and an iatrogenic VSD. Gender was reported as the proportion of females in the patient cohort. The surgical approach included three classes: median or subxiphoid sternotomy (the median approach), and the left and right intercostal space beside the sternum approach (left approach and right approach, respectively). If patients were lost to follow-up in the trial, we performed an intention-to-treat (ITT) analysis.

The quality assessment of all selected studies was independently performed by the two investigators (Yun-Han Jiang and Hua-Li Peng). All eligible randomized controlled trials were assessed using the Cochrane Risk of Bias assessment tool. This tool included the following items: allocation sequence generation, allocation concealment, participant masking, personnel and outcome assessors, completeness of outcome data, and selective outcome reporting. The Newcastle-Ottawa Scale (NOS) was used to assess the quality of the cohort studies and the case-control studies^13,14^. For cohort studies, quality was evaluated based on three major components: selection of the study group (0–4 stars), quality of the adjustment for confounding variables (0–2 stars) and the assessment of outcome in the cohorts (0–3 stars). Case-control studies were also assessed based on three factors: selection of the case and controls (0–4 stars), comparability of the cases and controls (0–2 stars) and the ascertainment of exposure (0–3 stars). Furthermore, we chose an 18-item, validated quality appraisal tool to evaluate the methodological quality of the case series^15^. The quality assessments for each item were binary determinations of various aspects of the study, including the study objective; study population; intervention and co-intervention; outcome measures; statistical analysis; the results and conclusions; competing interests; and sources of support. The score of the assessment was the number of yes responses, and the score of acceptable quality was ≥14^15,16^. Disagreements in the quality assessment were resolved through discussion.

### Statistical Analysis

All data syntheses and analyses were performed in STATA version 14.0 (Stata Corporation, College Station, Texas, USA) and Review Manager 5.3 (Cochrane Collaboration, Oxford, UK). Dichotomous data were expressed as relative risks (RRs) and 95% confidence intervals (CI) for the RCT and cohort studies and as odds ratios (ORs) and 95% CIs for case-control studies, whereas continuous data were expressed as standard mean difference (SMD). A random-effects model was used to obtain a conservative estimate of treatment efficacy, which takes into account both within-study and between-study variability^17^. Furthermore, post hoc power analyses and trial sequential analyses (TSAs) were conducted to detect the statistical power and to control for random errors, respectively. The power analysis for this meta-analysis was performed using Power and Precision V4. If the power was ≥ 0.80, the conclusion was deemed convincing. TSAs were used to account for the amount of eligible evidence, with type I error, power and risk reduction settings of 5%, 80% and 20%, respectively for binary results and additional information (trials, patients, and events) obtained from the included trials^18^. If the Z curve of the cumulative meta-analysis crossed one of the monitoring boundaries or required information sizes, a firm conclusion was established and no further trials were required. TSAs were conducted using TSA software version 0.9 (beta) (Copenhagen Trial Unit). The heterogeneity test was performed with the I^2^ statistic; the level of significance was set at P < 0.10^17^, and an I^2^ > 50% indicated a high degree of heterogeneity^19,20^. Additionally, a sensitivity analysis was also performed. The reasons for post-operative complications, conversion to open-heart surgery and late-term reoperation were analyzed via meta-regression, including all data from the case series, RCTs, cohort studies and case-control studies. The estimated incidence of complications was calculated using the Freeman-Tukey double arcsine transformation^21^, which transformed the incidence distribution into a normal distribution. Potentially relevant factors included the surgical approach (median approach, left approach or right approach); type of VSD (mVSD, pmVSD, dcsVSD or the special type of VSD); the size of the VSD and occluder; and the age, gender and weight of the patients. Publication bias was tested using a funnel plot and Egger’s test^22^. We used a trim-and-fill method to provide an adjusted summary RR, including the potential missing trials, if publication bias was evident^23^. Empirical continuity correction was used for double-zero studies, which added 0.5 to the number of events and non-events in both groups^24^. All analyses were conducted at the 2-sided significance level of 0.05.

## Results

### Literature selection and baseline characteristics

**Figure 1:**
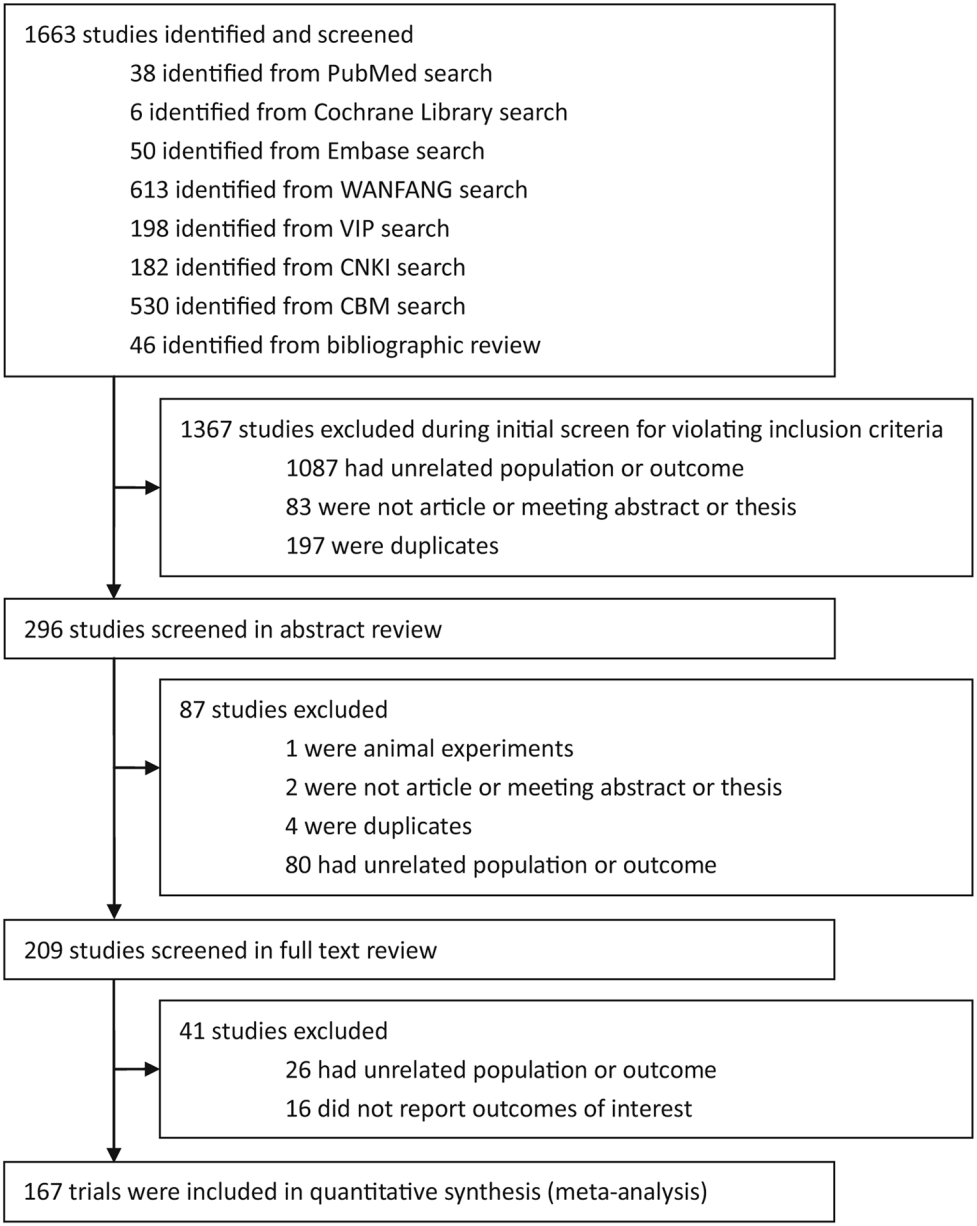
Data flow chart of study selection.

In total, 1,663 potential articles were initially retrieved through the database searches and bibliography reviews (Fig. 1). Consequently, 167 studies, consisting of 17,227 patients, were included in the meta-analysis^4–6,11,12,25–186^. Among the 167 included trials, 23 control studies, including 5 RCTs, 6 prospective cohort studies and 12 retrospective case-control studies, focused on the efficacy of TTDC and COHS for the treatment of VSDs. The remaining 144 articles reported only the results of TTDC for VSDs, including 1 cohort study, 1 case-control study, 129 case series (115 retrospective case series and 14 prospective case series) and 13 case reports. All articles underwent ITT analysis.

Among all the selected articles, 146 trials were conducted primarily in China^4–6,12,25–34,36–53,55–59,61,63,65–160,162,167,168,171–175,180,185,186^, 3 articles were conducted in other Asian countries (one each in India, South Korea and Turkey)^166,169,184^, 10 studies were conducted in the United States^11,62,64,163,170,176,178,179,181,182^, 5 studies were performed in European countries (one each in Austria, France, Germany, Poland, Russian Federation)^35,54,161,165,183^, 2 articles were conducted in South American countries (1 Brazil and 1 Chile)^164,177^, and 1 case-control study was performed in Australia^60^. A total of 108 articles were written in Chinese^5,12,26–29,31,32,36–43,46,70–132,134–159,174,175^, whereas 58 articles were written in English^4,6,11,25,30,33–35,44,45,47–69,133,160–163,165–173,176–186^, and the remaining article was written in Portuguese^164^. All trials were published between 1998 and 2016. The basic characteristics of the included studies are presented in Supplemental Table 1.

All included RCTs were judged to be at moderate risk of bias (Supplemental Fig. 1) because the surgical interventions might have impeded blinding of the participants and personnel. Three of the RCTs did not report the methods of allocation and one lacked the method of random sequence generation. Most cohort studies (71.43%) and case-control studies (61.54%) received a maximal number of stars according to the NOS tool (Supplemental Tables 2 and 3)^5,30–34,38–43,45^. Of the included cohort studies, one had selected an inadequate non-exposed cohort, and one did not describe the comparability of cohorts. Among the 13 case-control studies, two studies had not selected an adequate control and non-response rate for exposure and one had not assessed the results of cases and controls separately but instead reported the combined outcome. Two case-control studies did not mention the comparability of cases and controls, and one only controlled for age. Of the 129 identified case series, 56 studies (43.42%) were considered to be of acceptable quality according to the 18-item, validated quality appraisal tool^47–50,52,54,56,58,60,61,64,66,68–70,72–74,76,81,89,92,95,98,103–105,113,115,117,125,128,129,131,135,136,138,140,141,145,146,149,152,153,155,156,158,160,162,164–166,168,171–173^ (Supplemental Table 4). Four series did not clearly elucidate the study objective in the abstract, introduction or methods section; five did not account for the characteristics of the included participants; 121 case series collected cases at a single centre; 85 did not apply the criteria for inclusion; two recruited participants inconsecutively; four did not clearly describe the primary or additional interventions; 20 did not clearly defined the outcome measures in the introduction or methods section; 116 did not use appropriate statistical tests to assess the relevant outcome; 17 did not report the length of follow-up; 59 did not report the loss of follow-up; 53 did not estimate the random variability of the outcomes; 1 did not report the adverse events; most of the series (n = 128) did not report the source of support for the study.

### Heterogeneity and sensitivity analyses

Heterogeneity among all three types of studies was high (I^2^ > 75%) for most of the clinical implications: intra-operative arrhythmia and residual shunts in cohort studies, and post-operative valvular regurgitation and residual shunts in cohort studies.

Meanwhile, a sensitivity analysis was used to test the robustness of the results. The pooled RR or OR of the primary results and the effect size of the single-arm meta-regression analysis were essentially unaltered by the exclusion of each selected study. The studies were also analyzed using random-effects and fixed-effects models and were tested by the trim-and-fill method (Supplemental Table 6).

### Publication bias

**Figure 2:**
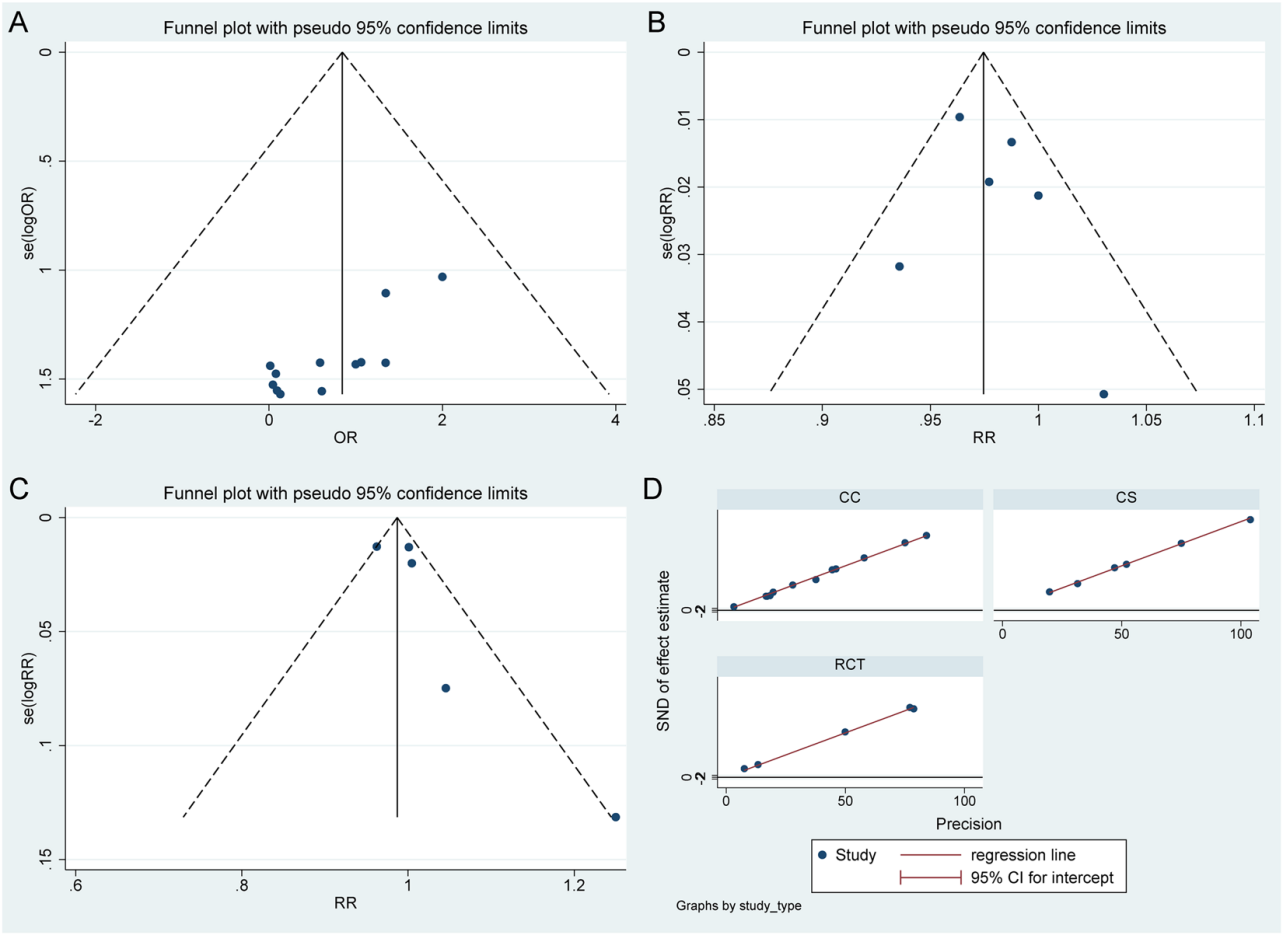
Publication bias of success rate. Funnel plots for success rate showed no significant asymmetry in case controls, (**A**) cohort studies (**B**) and randomized controlled trials (**C**), which are consistent with Egger’s regression plot for success rate in all three study types (**D**). CC, case controls; CS, cohort study; RCT, randomized controlled trial.

For most of the results, including success rate (p = 0.686), no statistical evidence of publication bias was detected by a funnel plot and Egger’s test (Fig. 2; Supplemental Table 5). However, given the limited data of several outcomes, such as intra-operative aortic regurgitation (AR), the publication bias was obvious, although not detected by Egger’s test. In addition, Egger’s test and a funnel plot analysis indicated significant asymmetry in the outcomes of a single-arm meta-regression analysis (Supplemental Table 6) because of the presence of publication bias for case series and case reports.

### Clinical implications

**Figure 3:**
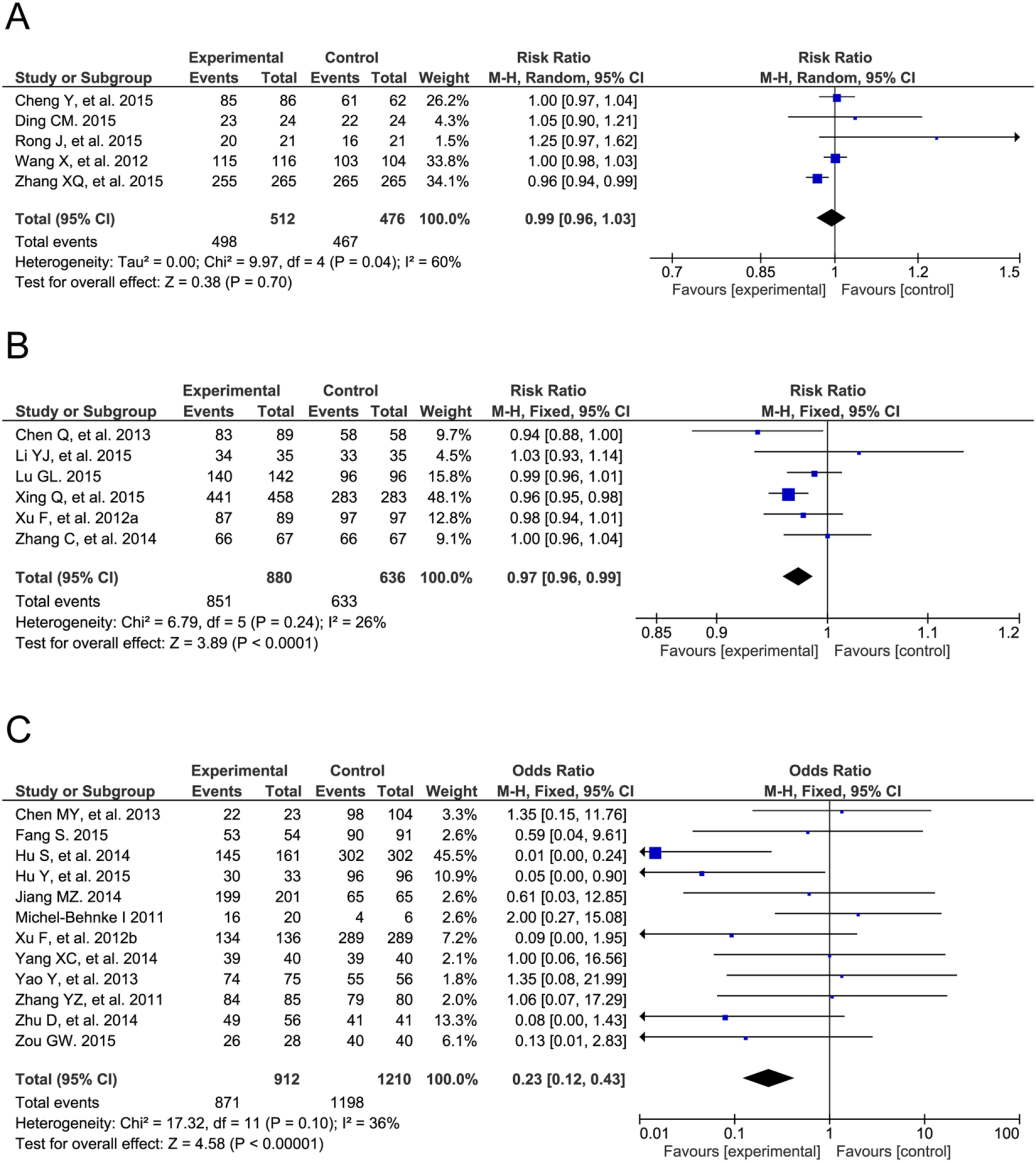
Forest plot of success rate. There is no significant difference in success rate in RCTs between TTDC and COHS (**A**). However, the success rate of TTDC is less than that of COHS in cohort studies (**B**) and case controls (**C**).

The success rate was defined as the rate of patients without fatal or serious early-term or late-term complications requiring reoperation. Data regarding the success rate were obtained from 23 trials involving 4,626 participants, including five RCTs, six prospective cohort studies and 12 retrospective case-control studies. The success rate was found to be significantly lower in the TTDC group than in the COHS group in cohort studies (RR = 0.97, 95% CI: 0.96 to 0.99, P < 0.0001) and case-control studies (OR = 0.23, 95% CI: 0.12 to 0.43, P < 0.00001). However, there were no significant differences among RCTs (RR = 0.99, 95% CI: 0.96 to 1.03, P = 0.70). There was no evidence of between-study heterogeneity in the cohort studies (I^2^ = 26%, P = 0.24) and case-control studies (I^2^ = 36%, P = 0.10), whereas moderate heterogeneity was observed in the RCTs (I^2^ = 60%, P = 0.04; Fig. 3).

A trial sequential analysis yielded an estimated diversity-adjusted required information size (DARIS) of 316,373 individuals in the RCTs. However, only 0.31% of the DARIS was reached, and the cumulative Z curve was located between the trial sequential monitoring boundaries and the conventional boundaries (Supplemental Fig. 2). A post hoc power analysis of the success rate yielded a value of 14%, indicating a 14% likelihood that the TTDC success rate is equal to that of COHS in the RCTs. Besides the success rate, other clinical outcomes were also compared among the three study types, including the duration of the operation, length of stay (LOS) in the intensive care unit (ICU), average length of stay (ALOS) in the hospital as an in-patient, transfusion rate and hospital costs (Supplemental Table 5). The summary outcomes showed that when compared with COHS, TTDC could decrease the durations of the procedure, ICU stay and hospital stay, as well as the number of transfusions. However, there were no reductions in the total cost.

### Intra-operative complications

**Figure 4:**
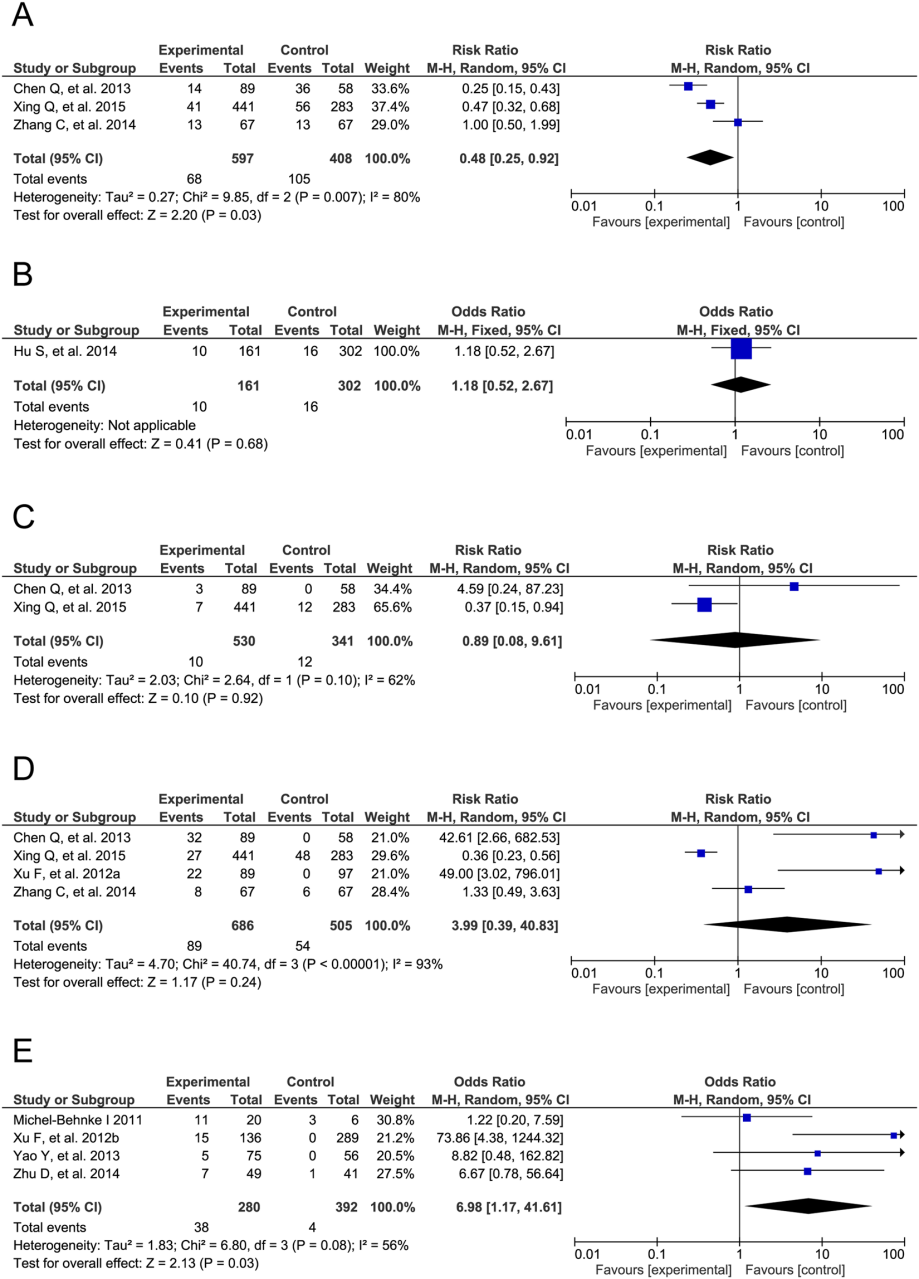
Forest plots of intra-operative main complications. TTDC reduce the risk of intra-operative arrhythmias in cohort studies (**A**), and has non-significant effect in case control (**B**). Furthermore, there is no significant difference in intra-operative aortic valve insufficiency (**C**) and residual shunts (**D**) between TTDC and COHS in cohort studies. However, results of cohort studies showed a higher risk in intra-operative residual shunts in TTDC (**E**).

Regarding intra-operative arrhythmias, the included case-control studies showed no significant differences between TTDC and COHS (OR = 1.18, 95% CI: 0.52 to 2.67, P = 0.68). By contrast, in the cohort studies, TTDC was shown to reduce the risk of intra-operative arrhythmias when compared with COHS (RR = 0.48, 95% CI: 0.25 to 0.92, P = 0.03; Fig. 4). The different types of arrhythmias were also analyzed in all three study types (Supplemental Table 5). However, no significant difference in intra-operative aortic valve insufficiency between TTDC and COHS was observed in the cohort studies (RR = 0.89, 95% CI: 0.08 to 9.61, P = 0.92; Fig. 4).

The intra-operative residual shunts rate was calculated as the occurrence of moderate to large residual shunts during TTDC or COHS. No significant differences in this rate were observed in the cohort studies (RR = 3.39, 95% CI: 0.39 to 4.83, P = 0.24), whereas TTDC was associated with a higher risk of intra-operative residual shunts, compared with COHS (case-control studies: OR = 6.98, 95% CI: 1.17 to 41.61, P = 0.03; Fig. 4). Additionally, the risks of other intra-operative complications, including left ventricular dysfunction, were analyzed in all three study types (Supplemental Table 5).

### Post-operative complications

**Figure 5:**
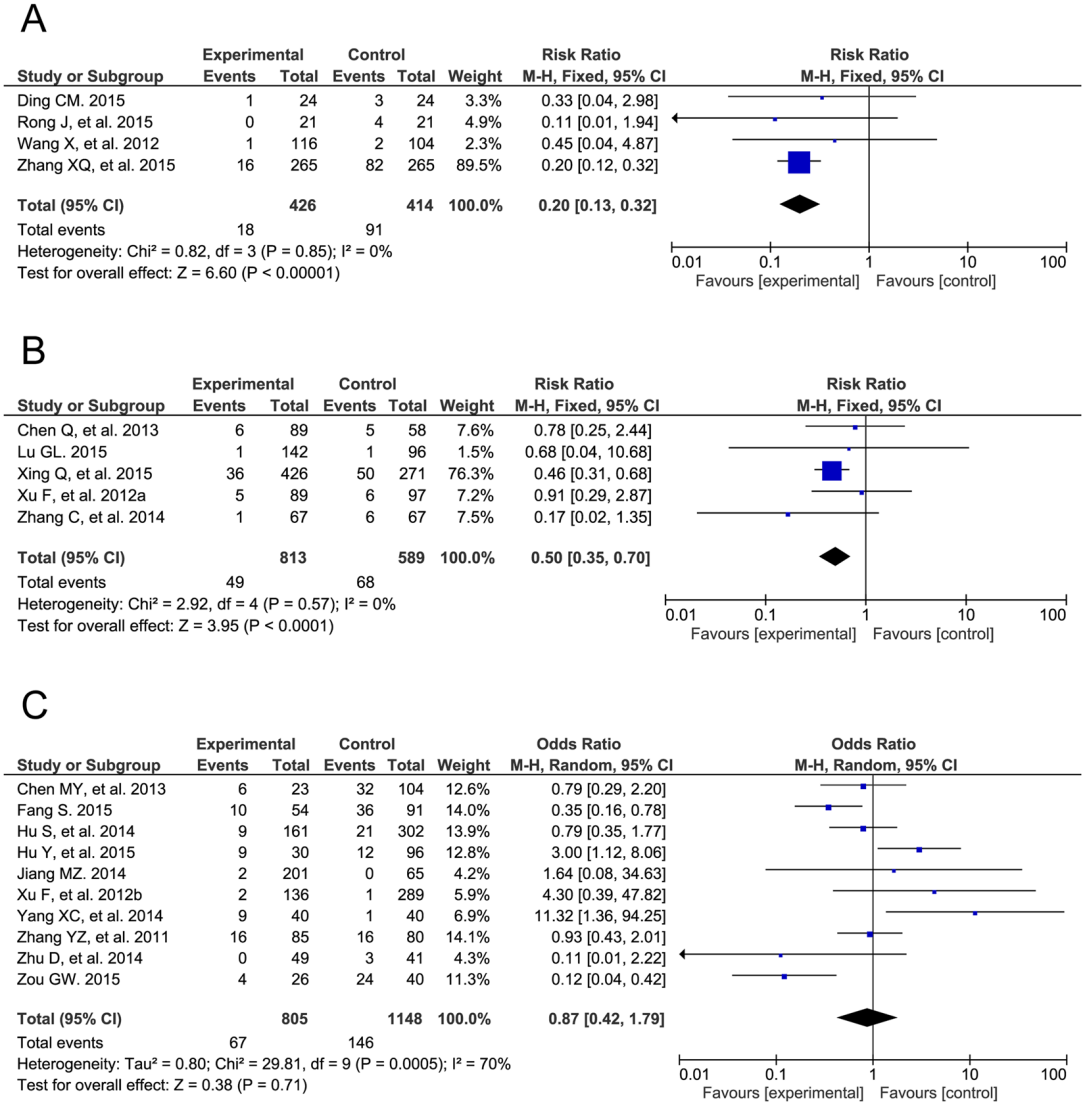
Forest plot of post-operative arrhythmia. The post-operative arrhythmias of TTDC is less than that of COHS in RCTs (**A**) and cohort studies (**B**), and is as the same as that of COHS in case controls (**C**).

**Figure 6:**
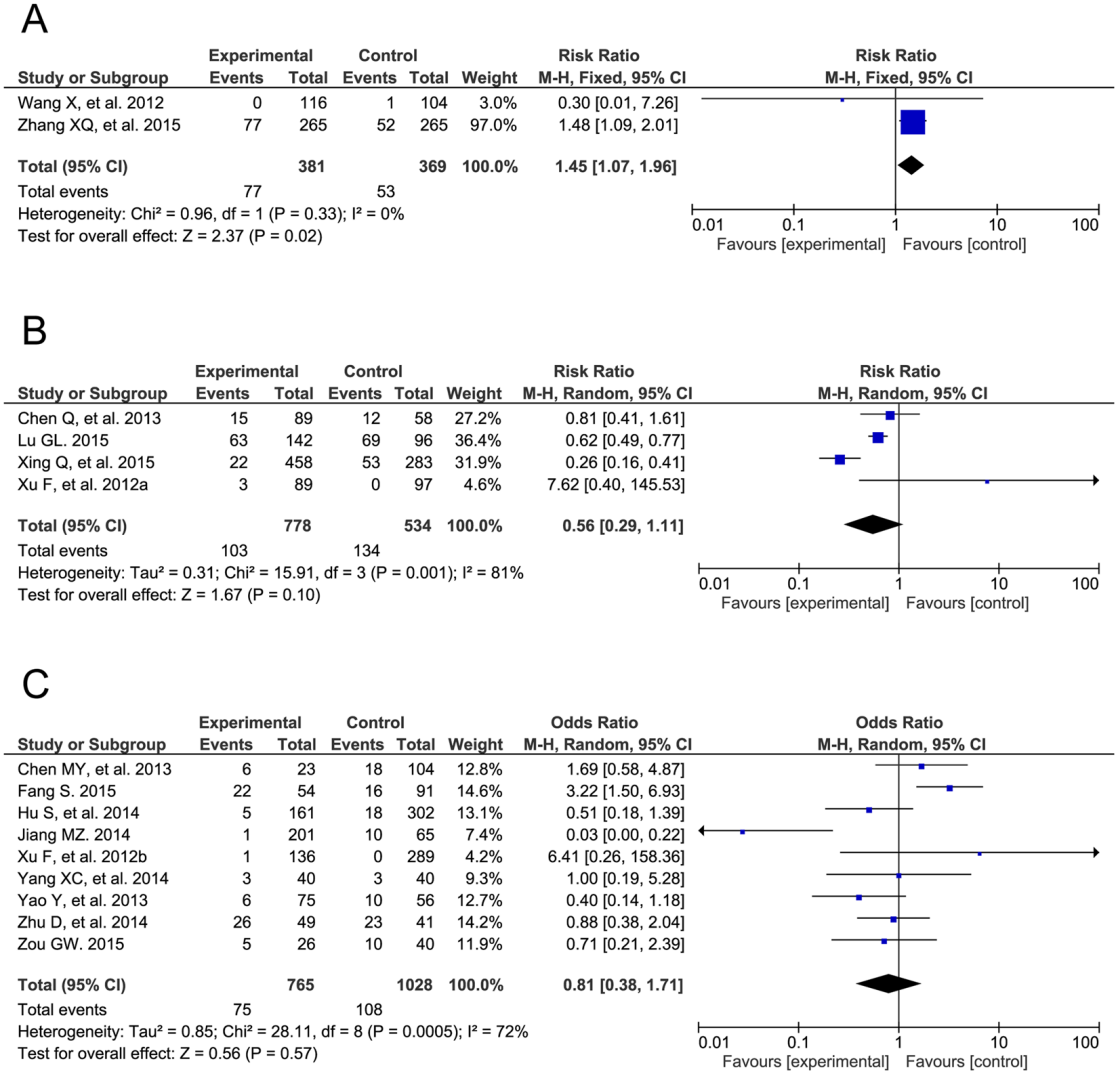
Forest plot of post-operative valvular insufficiency. TTDC has less risk of post-operative valvular insufficiency than that of COHS in RCT (**A**), and no significant advantages in cohort study group (**B**) and case control group (**C**).

**Figure 7:**
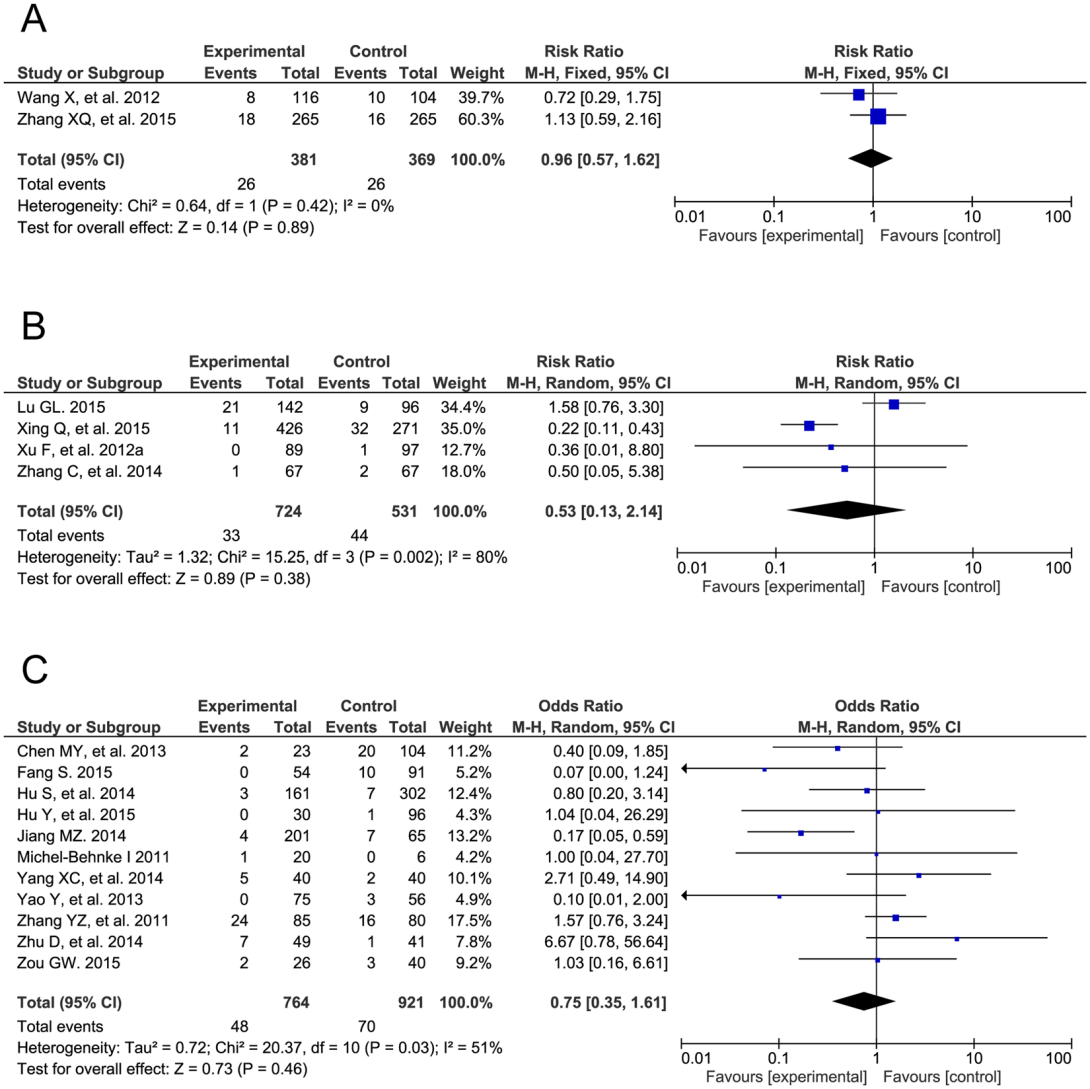
Forest plot of post-operative residual shunt. There is no significant difference in post-operative residual shunt between TTDC and COHS in RCTs (**A**), cohort studies (**B**) and case controls (**C**).

Regarding the post-operative complication of arrhythmia, we observed significant improvements in four of the RCTs (RR = 0.20, 95% CI: 0.13 to 0.32, P < 0.00001) and five of the cohort studies (RR = 0.50, 95% CI: 0.35 to 0.70, P < 0.0001) but no evident differences in 10 of the case-control studies (OR = 0.87, 95% CI: 0.42 to 1.79, P = 0.71; Fig. 5). The different types of arrhythmias were also analyzed among all three study types (Supplemental Table 5). There were different results in post-operative valvular regurgitation in the three groups (RCT: RR = 1.45, 95% CI: 1.07 to 1.96, P = 0.02; cohort study: RR = 0.56, 95% CI: 0.29 to 1.11, P = 0.10; case-control study: OR = 0.81, 95% CI: 0.38 to 1.71, P = 0.57; Fig. 6). The detailed pooled results of valvular regurgitation are listed in Supplemental Table 5. Furthermore, a post-operative residual shunt was defined as a more-than-trivial residual shunt after TTDC or COHS. Additionally, TTDC showed no improvement compared to COHS on post-operative residual shunts (RCT: RR = 0.96, 95% CI: 0.57 to 1.62, P = 0.89; cohort study: RR = 0.53, 95% CI: 0.13 to 2.14, P = 0.38; case-control study: OR = 0.75, 95% CI: 0.35 to 1.61, P = 0.46; Fig. 7). The risks of other intra-operative complications, such as incision infection, were also analyzed in all three study types (Supplemental Table 5).

### Reasons for post-operative complications

**Figure 8:**
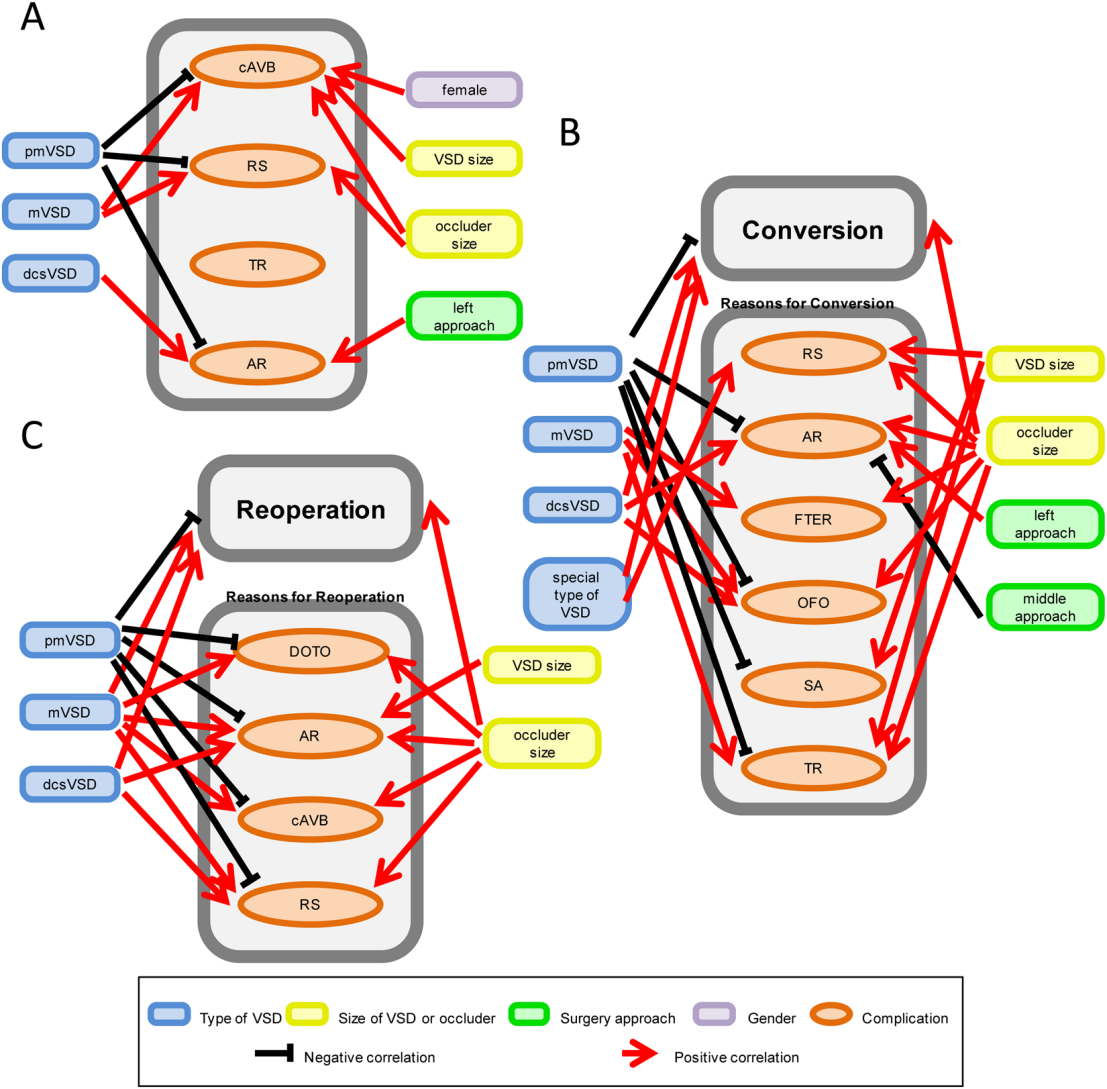
The relevant factors of main post-operative complications (**A**), conversion to conventional open-heart surgery (**B**) and reoperation (**C**). cAVB, complete atrioventricular block; RS, residual shunt; TR, tricuspid regurgitation; AR, aortic regurgitation; VSD, ventricular septal defect; pmVSD, perimembrane ventricular septal defect; mVSD muscular ventricular septal defect; dcsVSD, doubly committed subarterial ventricular septal defect; FTER, fail to establish the conveying rail; OFO, occluder fall off; SA, severe arrhythmia; DOTO, dropout of the occluder.

To identify the more specific indications of TTDC, we conducted single-arm meta-regression analyses for post-operative complications and the reasons for conversion to COHS (including post-operation before discharge and reoperation). A meta-regression analysis identified factors that increased the RR of post-operative complete atrioventricular block, including mVSD (p = 0.002), larger VSD (p = 0.013), female gender (p = 0.023) and larger occluder (p = 0.006), whereas pmVSD was associated with a lower RR (p = 0.009, Fig. 8 and Supplemental Table 7). The pooled results of post-operative residual shunts showed that pmVSD was a protective factor (p = 0.000), whereas mVSD (p = 0.000) and the size of the occluder (p = 0.028, Fig. 8 and Supplemental Table 7) were risk factors. Moreover, the occurrence of post-operative AR was lower among patients with pmVSD (p = 0.000), whereas patients with dcsVSD (p = 0.000) had an increased RR of AR. Additionally, the left approach (p = 0.033, Fig. 8 and Supplemental Table 7) was associated with adverse effects. However, none of the identified factors had significant protective or adverse effects on post-operative tricuspid regurgitation (Fig. 8 and Supplemental Table 7).

### Reasons for conversion to open-heart surgery in the perioperative period

A total of 161 studies involving 14,321 patients were analyzed to determine the reasons for conversion to COHS during the perioperative period. The analysis revealed that pmVSD was the only protective factor (p = 0.003), whereas dcsVSD (p = 0.048), the special type of VSD (p = 0.022) and the size of the occluder (p = 0.001) increased the risk of conversion to COHS. Notably, residual shunting was the main reason for a conversion from TTDC to COHS. In a meta-regression analysis, the special type of VSD (p = 0.006), size of the VSD (p = 0.025) and size of the occluder (p = 0.001, Supplemental Table 7) were identified as significant risk factors for residual shunts and, therefore, for conversion. AR was the second main reason for conversion to COHS. A meta-regression analysis showed that the left approach (p = 0.001), dcsVSD (p = 0.001) and the size of the occluder (p = 0.039) increased the relative risk of AR, whereas the middle approach (p = 0.012) and pmVSD (p = 0.000) were protective factors for AR. A multivariate meta-regression analysis found that the size of the occluder was an independent risk factor for AR.

A failure to establish the occluder conveying rail during the operation was the third reason for conversion to COHS, and here, the size of the occluder (p = 0.032) and mVSD (p = 0.032, Fig. 8 and Supplemental Table 7) were risk factors. Occluder fall-off was also an important reason for conversion to COHS. Meanwhile, the type of VSD was a significant predictor of occluder fall-off and, therefore, of conversion; mVSD and dcsVSD (P = 0.001 and 0.005, respectively) were associated with adverse effects, whereas pmVSD was associated with positive outcomes (p = 0.000, Supplemental Table 7). The size of the occluder also increased the risk of occluder fall-off (OFO). Severe arrhythmia was another main reason for conversion to COHS. The incidence of severe arrhythmia was greater with a larger VSD (p = 0.013) but smaller among cases of pmVSD (p = 0.014, Supplemental Table 7). Finally, tricuspid regurgitation was also cited as a reason for the high incidence of conversion to COHS.. The sizes of the VSD (p = 0.001) and occluder (p = 0.002) and mVSD (p = 0.017) were risk factors for tricuspid regurgitation, whereas pmVSD (p = 0.003, Fig. 8 and Supplemental Table 7) was identified as a beneficial factor.

### Reasons for performing open-heart reoperation

Reoperation was found to be a rare event among patients who underwent TTDC. Among the included trials, 167 trials with 14,921 patients presented reoperation data. In meta-regression analyses, mVSD (p = 0.001), dcsVSD (p = 0.008) and the size of the occluder (p = 0.021) were found to increase the risk of reoperation, whereas pmVSD (p = 0.000) was an independent protective factor. mVSD (p = 0.001) and the size of the occluder (p = 0.042) were risk factors for occluder fall-off, whereas pmVSD (p = 0.000) was less strongly associated with occluder fall-off. mVSD (p = 0.003), dcsVSD (p = 0.002), a larger VSD (p = 0.032) and a larger occluder (p = 0.018) contributed to an increased risk of aortic valve insufficiency, whereas pmVSD (p = 0.000) was associated with a decreased risk. Furthermore, mVSD (p = 0.001) and the size of the occluder (p = 0.017) were risk factors for a complete atrioventricular block, whereas pmVSD (p = 0.001) was associated with a reduced occurrence. In addition, pmVSD (p = 0.000) was associated with a reduced incidence of residual shunts, whereas mVSD (p = 0.001), dcsVSD (p = 0.029), and a larger occluder (p = 0.022, Fig. 8 and Supplemental Table 7) were identified as risk factors for residual shunts.

## Discussion

TTDC is a new treatment for VSDs. In 1998, Amin *et al*. 181 first used an Amplatzer occluder to performed TTDC for mVSD in an animal model and an infant. Since then, many articles have described the use of TTDC with the Amplatzer occluder in VSD patients^52,57,64,181^. More recently, this technology has been widely applied throughout China^4–6,12,25–34,36–53,55–59,61,63,65–160,162,167,168,171–175,180,185,186^. Through this systematic review, we have attempted to compare the outcomes of TTDC versus COHS.

All included studies including RCTs, cohort studies, case-control studies, case series and case reports, carried a moderate risk of bias. The surgical nature of the intervention limited the blinding of the participants and personnel, and this contributed to the primary risk of bias in the RCTs. By contrast, all but two of the identified cohort studies had a low risk of bias. Among the case-control studies, the main risk of bias was associated with the comparability of cases and controls, while the selection of controls and the non-response rate were the other sources of bias. The moderate risk of bias among case series mainly resulted from the collection of cases at a single centre, the lack of statistical analysis and the failure to report sources of support. Furthermore, many case series provided detail clinical information about several cases but did not conduct statistical analysis or estimations of random variability. The majority of case series studies merely included several special cases but did not establish rigorous inclusion criteria. Furthermore, some of the case series were retrospective studies and therefore did not include follow-up data.

Although 6 cohort studies and 12 case-control studies demonstrated an increased RR for success with TTDC, the five RCTs showed no significant differences in the success rates between TTDC and COHS. First, patients in the TTDC and COHS groups of the RCTs were selected according to the indication of TTDC. Therefore, the results of these trials were less clinically heterogeneous. Furthermore, the DARIS indicated that more than 316 thousand patients would be needed to obtain a convincing conclusion, suggesting that there were few differences between these two types of surgery (albeit with low statistical power). In addition, the 95% CIs for the RRs of the RCT data (0.96 to 1.03) included a RR of 0.97 for the cohort study data. The OR of the case-control study data was much lower than anticipated, which was partly attributable to the lack of strict patients selection criteria. Furthermore, of the RCTs, only that of Zhang and colleagues^25^ reported a lower success rate with TTDC than with COHS, and the cases of TTDC failure were largely attributed to an inability to find a suitable type and size of occluder; this itself resulted from the selection of unsuitable cases for TTDC. Similarly, the inclusion criteria of the cohort studies conducted by Chen *et al*.^29^ and Xing *et al*.^4^ and a case-control study by Hu *et al*.^33^ did not include limitations regarding the position and size of the VSD or other specific clinical conditions. In the study by Hu *et al*.^44^, approximately 10% of the cases of TTDC conversion to COHS were attributed to unsuitable occluders, as all complications were resolved by removing the occluders. These outcomes may be the consequence of a lack of multiple attempts with different types and sizes of occluders. Therefore, among selected patients, the success rates of TTDC and COHS may be identical. However, additional rigorous, large-scale RCTs are required to address this point.

Regarding intra-operative complications, we found no convincing evidence of a reduced risk of arrhythmias, aortic insufficiency or residual shunts in the TTDC group, due to contradictions between the results of cohort studies and case-control studies or limited evidence. In other words, TTDC was not inferior to COHS in terms of intra-operative complications.

We found that the TTDC group had a lower RR of post-operative arrhythmias than did the COHS group in the RCTs and cohort studies. TTDC is a minimally invasive operation that requires no sutures and interferes less with the anatomy surrounding the VSD (i.e., conduction tissue). In a case-control study by Yang *et al*.^37^, post-operative arrhythmia may be more likely to result from procedure-related early inflammation and edema, as these conditions can be treated by glucocorticoids. This procedure-related complication may be caused by a lack of experience with TTDC. In another study of the increasing risk of post-operative arrhythmia with TTDC^33^, all cases of post-operative transient sinus or superventricular arrhythmia were reversed to a sinus rhythm within 48-72 h after surgery. This finding may be attributable to early procedure-related inflammation or the limited number of cases in the TTDC group. TTDC had no significant effect on post-operative residual shunts, as shown by the inconsistent outcomes from the RCT, cohort study and case-control study data. The intensity of evidence from the RCTs and cohort studies allowed us to conclude that TTDC reduces the incidence of post-operative arrhythmia, but we acknowledge that additional trials are needed to confirm this conclusion. Additionally, the three study types yielded conflicting results regarding valvular regurgitation, and therefore additional large-scale RCTs are required to reach a conclusion.

According to the current findings regarding clinical indices, intra-operative complications and post-operative complications, TTDC is slightly safer than COHS in terms of several post-operative complications (including arrhythmias) but is no less safe than COHS in terms of other complications. Meanwhile, TTDC provides more advantages than COHS in some clinical indices, including the duration of operation, i.e., TTDC is a promising treatment for suitable types of VSDs. To determine more suitable indications for TTDC, the reasons for complications, conversion to COHS and reoperation were also comprehensively analyzed.

In our study, we found that several clinical factors influenced the occurrence of post-operative complications. First, the type of VSD has a major effect on many complications and is identified as a reason for conversion. TTDC may be highly recommended for the treatment of pmVSD, which is associated with a reduced RR of post-operative cAVB, residual shunts, and AR; reduce occurrence of conversion due to AR, occluder fall-off, severe arrhythmia, or tricuspid regurgitation and a reduced prevalence of late-term reoperation due to occluder drop off, AR, complete atrioventricular block, and residual shunts. Additionally, pmVSD is an independent protective factor for conversion to COHS due to occluder fall-off and AR, as well as for reoperation. Regarding pmVSD, it is easier to adjust the delivery sheath to a vertical position relative to the VSD^4^, thus avoiding damage to or interference with the surrounding tissues. However, when advancing a flexible guidewire to the mVSD, the angle of the sheath and ventricular septum deviates slightly from the right, and more attempts are generally required to establish the occluder conveying rail. Meanwhile, there are multiple coarse right ventricular trabeculations surrounding mVSD, which increases the difficulty of localizing the occluder and predisposes the patient to residual shunts. According to the Anderson classification^9,10^, inlet VSDs are classified as mVSDs and are more likely to cause post-operative cAVB^187,188^, which occurs in close proximity to the conduction tissue. In addition, the anatomy of dcsVSD contributes to its association with AR, as its features may interfere with the aortic valve and require a perpendicular approach^45^. The sharp and relatively stiff edge of the left disc of the occluder and its impingement of the aortic valve may also result in a high risk of post-operative AR among patients with dscVSD. However, the dcsVSD is located far away from the conduction system, and TTDC for dcsVSD therefore has no adverse effects on cAVB or conversion and reoperation for severe arrhythmias. Therefore, when patients with other types of VSDs (mVSD, dcsVSD, the special type of VSD) undergo TTDC, we need to be more careful to prevent post-operative complications (mVSD: cAVB and residual shunt; dcsVSD: AR) and avoid conversion to COHS for a myriad of reasons (the special type of VSD: residual shunt; mVSD: occluder fall off, tricuspid regurgitation and failure to establish the rail; dcsVSD: AR and occluder fall off). These other types of VSDs also tend to be associated with increased risk of late-term retreatment (mVSD: dropout off the occluder, AR, complete atrioventricular block and residual shunts; dcsVSD: AR and residual shunts).

The sizes of the VSD and occluder were other important risk factors for complications, conversion and reoperation. Both factors are susceptible to constriction of the atrioventricular conduction and interference with the surrounding tissues and could thus lead to an increased incidence of post-operative cAVB, conversion due to residual shunts and tricuspid regurgitation and reoperation due to AR. While the size of the VSD was associated with an increased risk of conversion due to severe arrhythmia, larger occluders are associated with an increased risk of post-operative residual shunts and conversion due to residual shunts, which are consistent with the findings of many studies^173^; AR; occluder fall off; failure to establish the rail; and increased risk of reoperation due to occluder fall off, complete atrioventricular block and residual shunts. A larger the occluder size increases the potential stretching of the surrounding tissue, including conduction tissue, and thus increases the risks of complication, conversion and reoperation. Similarly, An and colleagues recommended TTDC for patients with a relatively small VSD size^189^.

Finally, the choice of surgical approach could also affect the rates of complication and conversion. The median or subxiphoid approach is associated with a reduced RR of conversion due to AR without any other adverse effect; i.e., the middle approach leads to an easier operation. Nevertheless, the left intercostal space approach appeared to be associated with an increased probability of post-operative AR and conversion due to AR, as this approach is associated with great difficulty in establishing the occluder conveying rail and adaptation to the intracristal and subcristal VSD, which are near the aorta. The left approach is more suitable for dcsVSD because the left second intercostal space directly face the pulmonary annulus, and this access point is better enables a perpendicular approach to the infundibular septum. In these analyses, age was not a significant factor, probably because it was presented as an average value in each trial rather than as individual patient data.

This study had some limitations of note. First, only five RCTs were included, and therefore, subgroup analyses could not be performed. Only one or two studies were included in the assessment of intra- or post-operative complications. Given the limited evidence, we observed non-significant effects in the presented results. To increase the amount of evidence regarding TTDC for the treatment of VSDs, cohort studies and case-control studies were also included. Second, the findings of the RCTs, cohort studies and case-control studies conflicted because of the diverse inclusion criteria used in each type of study. Therefore, a post hoc power analysis was performed to determine the power of the conclusions, and TSA was conducted to ascertain the trend of uncertain outcomes. Third, the follow-up durations ranged from 3 months to 6 years, and therefore the assessment of post-operative complications of TTDC might have revealed significant heterogeneity that would neglect several long-term complications. Therefore, additional studies with longer and more consistent follow-up times are required. Finally, limitations affecting the meta-regression analysis must also be taken into account. Ecological bias should be considered because our findings are derived from aggregated data. However, the effect of covariates associated with the study outcomes may be confounded by those of other covariates not included in the analyses.

Our study has various strengths. We strictly followed the recommendations of the PRISMA guidelines when conducting the literature selection, data extraction and analysis, which allowed us to standardize the reporting and improve the clarity of the review. These results are objective and not affected by attrition bias due to the inclusion of an ITT analysis. In addition, this is the first meta-analysis to compare the efficacy and safety of TTDC to that of COHS for VSDs, as well as the first study to analyze the factors relevant to complications and the reasons for conversion to COHS and reoperation.

Our study suggests that when compared to COHS, TTDC is associated with reductions in the durations of surgery and ICU and hospital stays; number of transfusions and risk of post-operative arrhythmia, without leading to evident increases in other intra- or post-operative complications. A minimally invasive approach is preferable for suitable cases, and TTDC is more suitable for patients with pmVSD and smaller VSDs. Smaller occluders and a low median sternum or subxiphoid surgical approach are associated with fewer complications and a reduced risk of conversion to COHS and reoperation. Furthermore, when patients with mVSD undergo TTDC, additional care should be taken to prevent post-operative cAVB and residual shunts and to avoid conversion to COHS due to occluder fall-off, tricuspid regurgitation and a failure to establish the rail, as well as reoperation due to dropout from the occluder, AR, cAVB and residual shunts. The use of a smaller occluder with a longer connecting waist can reduce the risk of post-operative cAVB and reoperation for cAVB. Detailed pre-operative transthoracic echocardiography to evaluate the VSD position and intra-operative transesophageal echocardiography to supervise the process of TTDC should be considered to avoid the conversion and reoperation for this TTDC-related complication. Regarding dcsVSD, the left approach is recommended to better establish the rail and deploy the occluder, thus reducing the incidence of occluder fall-off. Multiple attempts under close transesophageal echocardiography monitoring can lower the risk of post-operative AR, conversion and reoperation for AR. The clinical practice guidelines should be updated to reflect the evidence presented herein.

Further research is required to determine the VSD size considered suitable for TTDC and the relationship between the sizes of the VSD and the occluder. More rigorous, larger-scale multi-center RCTs with consistent long-term follow-up may provide more meaningful information regarding the complications associated with TTDC. The mechanisms underlying the associations of the relevant factors with complications, conversion or reoperation should also be clarified, as this information will help us to better understand the indications and considerations of TTDC.

## Conclusions

According to the current evidence, TTDC is not inferior to COHS and is associated with a shorter duration of surgery, shorter ICU and hospital stay, fewer transfusions and a decreased incidence of post-operative arrhythmia. TTDC is more suitable for patients with pmVSD and smaller VSDs; additionally, smaller occluders the low median sternum or subxiphoid approach are highly recommended. Patients with larger or other types of VSDs (mVSD, dcsVSD or a special type of VSD) and in whom a larger occluder and other surgical approaches (left or right approach) have been used, as well as female patients, require additional attention because of an increased risk of post-operative complications, conversion to COHS and reoperation. Additional rigorous RCTs are needed to provide more information regarding complications.

## Electronic supplementary material

Supplemental materials
