# Supplementary material for: Effects of transthoracic device closure on ventricular septal defects and reasons for conversion to open-heart surgery: A meta-analysis
Source: Sci Rep. 2017 Sep 22;7:12219. doi: 10.1038/s41598-017-12500-6 (PMC5610181; doi:10.1038/s41598-017-12500-6)

**Effects of transthoracic device closure on ventricular septal defects and reasons for conversion to open-heart surgery: A meta-analysis**

**Yang Zhou^1^, Ling-Xi Liu ^1^, Fei Zhao^1^, Shi-Hai Tang^1^, Hua-Li Peng^1^, Yun-Han Jiang^2^***

^1^Department of Cardiothoracic Surgery, the People’s Hospital of Leshan, Leshan, Sichuan Province 614000, P.R. China.

^2^Department of Cardiovascular Surgery, Xinqiao Hospital, Third Military Medical University, Chongqing, Chongqing 400030, P.R. China.

^*^Correspondence and requests for materials should be addressed to Y-H.J. (email: jiangyunhan89@163.com)

Additional file

Appendix 1 the Preferred Reporting Items for Systematic Reviews and Meta-Analyses (PRISMA) 2009 Checklist

Appendix table and figures information

Supplemental tab A. Characteristics of Included Studies.

Supplemental tab B. Newcastle-Ottawa Scale (NOS) for Cohort Studies.

Supplemental tab C. NOS for Case Control Studies.

Supplemental tab D. Quality Appraisal for Case Series.

Supplemental tab E. Secondary Results of Clinical Index, Intra- and Post-operative Complications.

Supplemental tab F. Sensitivity Analyses of Complications, Conversion, and Reoperation.

Supplemental tab G. Relevant factors of Complications, Conversion and Reoperation.

Supplemental fig A. Risk of bias in randomized controlled trials. Risk of bias in randomized controlled trials is presented in risk of bias graph (A) and risk of bias summary (B).

Supplemental fig B. Trial sequential analysis (TSA) of success rate in randomized controlled trials. TSA showed that only 0.31% the DARIS has been reached and the cumulative Z-curve (blue) is located between the trial sequential monitoring boundaries (beyond the scale, not show) and the conventional boundaries (black) with an α of 5%, a β of 80%, and an absolute difference of 0.5% between the groups.


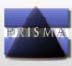
**PRISMA 2009 Checklist**

| **Section/topic** | **#** | **Checklist item** | **Reported on page #** |
| --- | --- | --- | --- |
| **TITLE** | | |  |
| Title | 1 | Identify the report as a systematic review, meta-analysis, or both. | 1 |
| **ABSTRACT** | | |  |
| Structured summary | 2 | Provide a structured summary including, as applicable: background; objectives; data sources; study eligibility criteria, participants, and interventions; study appraisal and synthesis methods; results; limitations; conclusions and implications of key findings; systematic review registration number. | 1 |
| **INTRODUCTION** | | |  |
| Rationale | 3 | Describe the rationale for the review in the context of what is already known. | 1,2 |
| Objectives | 4 | Provide an explicit statement of questions being addressed with reference to participants, interventions, comparisons, outcomes, and study design (PICOS). | 1,2 |
| **METHODS** | | |  |
| Protocol and registration | 5 | Indicate if a review protocol exists, if and where it can be accessed (e.g., Web address), and, if available, provide registration information including registration number. | 2 |
| Eligibility criteria | 6 | Specify study characteristics (e.g., PICOS, length of follow-up) and report characteristics (e.g., years considered, language, publication status) used as criteria for eligibility, giving rationale. | 2 |
| Information sources | 7 | Describe all information sources (e.g., databases with dates of coverage, contact with study authors to identify additional studies) in the search and date last searched. | 2 |
| Search | 8 | Present full electronic search strategy for at least one database, including any limits used, such that it could be repeated. | 2 |
| Study selection | 9 | State the process for selecting studies (i.e., screening, eligibility, included in systematic review, and, if applicable, included in the meta-analysis). | 2 |
| Data collection process | 10 | Describe method of data extraction from reports (e.g., piloted forms, independently, in duplicate) and any processes for obtaining and confirming data from investigators. | 3 |
| Data items | 11 | List and define all variables for which data were sought (e.g., PICOS, funding sources) and any assumptions and simplifications made. | 3 |
| Risk of bias in individual studies | 12 | Describe methods used for assessing risk of bias of individual studies (including specification of whether this was done at the study or outcome level), and how this information is to be used in any data synthesis. | 3 |
| Summary measures | 13 | State the principal summary measures (e.g., risk ratio, difference in means). | 3 |
| Synthesis of results | 14 | Describe the methods of handling data and combining results of studies, if done, including measures of consistency (e.g., I^2^) for each meta-analysis. | 4 |

| **Section/topic** | **#** | **Checklist item** | **Reported on page #** |
| --- | --- | --- | --- |
| Risk of bias across studies | 15 | Specify any assessment of risk of bias that may affect the cumulative evidence (e.g., publication bias, selective reporting within studies). | 3 |
| Additional analyses | 16 | Describe methods of additional analyses (e.g., sensitivity or subgroup analyses, meta-regression), if done, indicating which were pre-specified. | 4 |
| **RESULTS** | | |  |
| Study selection | 17 | Give numbers of studies screened, assessed for eligibility, and included in the review, with reasons for exclusions at each stage, ideally with a flow diagram. | 4, Fig 1 |
| Study characteristics | 18 | For each study, present characteristics for which data were extracted (e.g., study size, PICOS, follow-up period) and provide the citations. | 4, Table 1 |
| Risk of bias within studies | 19 | Present data on risk of bias of each study and, if available, any outcome level assessment (see item 12). | 5, Fig 2S |
| Results of individual studies | 20 | For all outcomes considered (benefits or harms), present, for each study: (a) simple summary data for each intervention group (b) effect estimates and confidence intervals, ideally with a forest plot. | 5-6 |
| Synthesis of results | 21 | Present results of each meta-analysis done, including confidence intervals and measures of consistency. | 5-6 |
| Risk of bias across studies | 22 | Present results of any assessment of risk of bias across studies (see Item 15). | 5 |
| Additional analysis | 23 | Give results of additional analyses, if done (e.g., sensitivity or subgroup analyses, meta-regression [see Item 16]). | 6-8 |
| **DISCUSSION** | | |  |
| Summary of evidence | 24 | Summarize the main findings including the strength of evidence for each main outcome; consider their relevance to key groups (e.g., healthcare providers, users, and policy makers). | 8,9 |
| Limitations | 25 | Discuss limitations at study and outcome level (e.g., risk of bias), and at review-level (e.g., incomplete retrieval of identified research, reporting bias). | 9, 10 |
| Conclusions | 26 | Provide a general interpretation of the results in the context of other evidence, and implications for future research. | 10 |
| **FUNDING** | | |  |
| Funding | 27 | Describe sources of funding for the systematic review and other support (e.g., supply of data); role of funders for the systematic review. | No funder |

*From:*  Moher D, Liberati A, Tetzlaff J, Altman DG, The PRISMA Group (2009). Preferred Reporting Items for Systematic Reviews and Meta-Analyses: The PRISMA Statement. PLoS Med 6(7): e1000097. doi:10.1371/journal.pmed1000097

For more information, visit: **www.prisma-statement.org**.

Supplemental Tab A Characteristics of Included Studies

|  | **Publication type** | **Study type** | **Country** | **Interventions** | **Number of patients** | **VSD type** | **Surgery approach** | **Occluder type** | **VSD size mm** | **Age (y)** | **Gender (women/men)** | **Weight (Kg)** | **Follow-up (months)** |
| --- | --- | --- | --- | --- | --- | --- | --- | --- | --- | --- | --- | --- | --- |
| Wang X et al (2012) | Article | RCT | China | TTDC/COHS | 116/104 | pm, m | Middle | NA | NA | 6.2±5.4 / 7.2±4.3 | 115/105 | 18.8±9.5 / 20.5±12.3 | NA |
| Zhang XQ et al (2015) | Article | RCT | China | TTDC/SRRIAT | 265/265 | pm, dcs | Middle | NA | 7.05±2.42 / 7.24±2.32 | 1.18±0.67 / 1.21±0.68 | 256/274 | 8.94±3.06 / 8.58±2.96 | NA |
| Ding CM (2015) | Article | RCT | China | TTDC/COHS | 24/24 | pm, dcs | Middle | NA | 4-8 | 5.6±1.3 / 5.4±1.2 | 19/29 | 18.7±3.8 / 18.4±3.6 | NA |
| Rong J et al (2015) | Article | RCT | China | TTDC/MTOHS | 21/21 | pm, m | Middle | NA | NA | 4.36±3.94 / 4.59±3.19 | 17/25 | 17.36±10.86 / 17.5l±10.93 | NA |
| Cheng Y et al (2015) | Article | RCT | China | TTDC/COHS | 86/62 | NA | NA | NA | NA | 9.7±2.1 / 10.2±2.2 | 77/71 | 4.3-66 / 4.8-71 | NA |
| Xing Q et al (2015) | article | PCS | China | TTDC/COHS | 458/283 | pm, m, dcs | Middle, Left | p-c, p-e, m, pda, sp1 | 5.21±2.95 / 6.83±3.75 | 0.95±0.56 / 0.86±0.71 | 348/393 | 9.82±5.88 / 8.56±7.79 | 47.31±19.69 / 41.23±18.47 |
| Chen Q et al (2013) | article | PCS | China | TTDC/COHS | 89/58 | pm | Middle | A4B2 | NA | 13.3±11.9 / 13.7±13.6 | 82/65 | 34.2±16.8 / 34.6±18.5 | 1.5±0.5 / 1.4±0.5 |
| Xu F et al (2012a) | article | PCS | China | TTDC/COHS | 89/97 | pm | Middle | p-c, p-e | 5.1±1.5 / 6.4±1.7 | 0.73±0.26 / 0.51±0.24 | 96/90 | 9.8±1.8 / 7.7±1.9 | NA |
| Zhang C et al (2014) | article | PCS | China | TTDC/COHS | 67/67 | pm, m, dcs | Middle, Left | NA | 6.1±3.4 / 8.6±5.1 | 6.3±2.4 / 5.8±2.7 | 72/62 | 17.3±3.9 / 15.0±3.3 | NA |
| Li YJ et al (2015) | article | PCS | China | TTDC/COHS | 35/35 | NA | Middle, Left | NA | NA | 2.1±0.8 / 2.1±0.9 | 47/23 | NA | NA |
| Lu GL (2015) | Masters' Thesis | PCS | China | TTDC/COHS | 142/96 | pm, m, dcs | Middle, Left | NA | 5.1±1.9 / 7.1±3.2 | 4.4±5.3 / 4.1±4.1 | 121/117 | 16.4±12.3 / 14.9±9.1 | NA |
| Zhang GC et al (2013) | Article | PCS | China | TTDC-tee/TTDC-tte | 30/41 | pm | Middle | p-c, p-e | 5.72±0.28 / 5.62±0.17 | 0.68±0.06 / 0.63±0.05 | 35/36 | 8.13±0.45 / 7.85±0.39 | 3.2±2.1 / 3.5±2.2 |
| Hu Y et al (2015) | Article | RCC | China | TTDC/SRRIAT | 33/96 | pm | Middle | p-c, p-e | 5.1±2.2 / 4.2±1.8 | 5.3±3.6 / 4.4±3.0 | 69/57 | 18.1±10.4 / 16.4±6.1 | 20 (12-30) |
| Michel-Behnke I et al (2011) | Article | RCC | Austria | TTDC/COHS | 20/6 | pm, m | Middle | AO-m, AO-mi, AO-pm, AO-a | 3±4.13 / 5.33±1.63 | 11.96±26.42 / 0.60±1.20 | NA | 15.42±22.35 / 5.07±2.77 | 11.31±11.36 / 33.32±12.06 |
| Zhang YZ et al (2011) | Article | RCC | China | TTDC/COHS | 85/80 | pm, m, dcs | Middle, Left | p-c, p-e, m, A4B2 | 6.3±3.6 / 8.8±5.3 | 9.2±10.2 / 8.8±6.2 | 93/72 | NA | NA |
| Chen MY et al (2013) | Article | RCC | China | TTDC/COHS | 23/104 | pm, m, dcs | NA | NA | 6.30±2.03 / 10.32±3.50 | 3.73±3.20 / 1.75±1.99 | 60/67 | 13.65±6.87 / 9.74±6.27 | 11 / 10 |
| Yang XC et al (2014) | Article | RCC | China | TTDC/COHS | 40/40 | pm | Middle | NA | 5.0±0.3 / 6.8±0.4 | 15.0±3.0 / 15.5±3.5 | 34/46 | 24.2±5.5 / 24.2±7.5 | NA |
| Jiang MZ (2014)# | Masters' thesis | RCC | China | TTDC/COHS | 201/65 | pm, m, dcs | Middle | NA | 4.86±1.04 / 4.76±1.22 | 1.43±0.45 / 1.41±0.64 | 138/128 | 9.04±1.65 / 9.17±1.50 | 7-27.3 / 3.7-26 |
| Xu F et al (2012b) | Article | RCC | China | TTDC/COHS | 136/289 | pm, m, dcs | Middle | NA | 5.7±2.2 / 6.1±2.8 | 1.4±1.0 / 1.3±0.7 | 191/234 | 11.8±4.8 / 11.2±5.0 | 15 (6-25) |
| Fang S (2015) | Masters' thesis | RCC | China | TTDC/COHS | 54/91 | dcs | NA | NA | 4.09±1.05 / 4.68±1.35 | 2.63 (1.81-4.71) / 2.00 (1.00-4.58) | 53/92 | 14.00 (11.00-17.13) / 12.00 (10.00-17.00) | NA |
| Zou GW (2015) | Masters' thesis | RCC | China | TTDC/COHS | 28/40 | m, dcs | Middle | NA | 6.12±1.46 / 6.23±1.51 | 6.87±6.73 / 9.19±8.88 | 24/42 | 21.43±13.32 / 26.48±16.64 | 26.8 (12-60) |
| Yao Y et al (2013) | Article | RCC | China | TTDC/COHS | 75/56 | pm, m, dcs | Middle | NA | NA | 4.72±0.37 / 4.62±0.49 | 53/78 | 18.37±1.01 / 17.71±1.22 | NA |
| Zhu D et al (2014) | Article | RCC | China | TTDC/COHS | 56/41 | dcs | Middle, Left | NA | 5.03±1.31 / 6.03±1.94 | 5.82±4.32 / 4.36±3.21 | NA | 20.28±11.24 / 16.80±8.90 | 3.0±1.5 |
| Hu S et al (2014) | Article | RCC | China | TTDC/COHS | 161/302 | pm, dcs | Middle, Left | NA | 6.95±1.90 / 6.81±2.13 | 3.70±2.31 / 3.82±2.43 | 223/240 | 16.70±7.46 / 15.6±5.0 | 12.6 (3-36) |
| Li HX et al (2011) | Article | RCC | China | TTDC-SBA/TTDC-HBA/TTDC-DT | 20/26/49 | pm, m | Middle, Left | p-c, p-e | NA | 9±10 | 53/44 | 27±20 | NA |
| Bai W et al (2012) | Article | RCSS | China | TTDC | 49 | pm | Middle | p-c, p-e | NA | NA | NA | NA | NA |
| Yang Y et al (2014) | Article | PCSS | China | TTDC | 942 | pm | Middle | p-c, p-e, A4B2 | NA | 4.61±3.37 | 441/501 | 18.01±7.25 | 21.32±8.67 |
| Ou-Yang WB et al (2015) | Article | PCSS | China | TTDC-TI | 187 | pm | Middle, Left | p-c, p-e | 5.31±2.86 | 8.2±10.2 | NA | NA | 12.6±10.4 |
| Wu Q et al (2012) | Article | RCSS | China | TTDC | 446 | pm, m, dcs | Middle | p-c, p-e, m, pda | 4.33±1.78 | 4.6±2.8 | 210/236 | 16.9±6.3 | NA |
| Chen Q et al (2010) | Article | PCSS | China | TTDC | 15 | dcs | Middle | p-e | 7.0±1.5 | 6.53±4.16 | 7/8 | 22.80±7.99 | 6.1±3.8 |
| Cao H et al (2011) | Article | PCSS | China | TTDC | 18 | pm | Middle | p-c, p-e | 6.5±1.0 | 1.0±0.6 | NA | 10.2±3.6 | NA |
| Quansheng X et al (2009) | Article | RCSS | China | TTDC | 21 | pm | Middle | p-c, p-e | NA | 3.6 (0.9-12) | 8/13 | 15.8±6.4 | ≥5 |
| Omelchenko A et al (2016) | Article | RCSS | Russian Federation | TTDC | 51 | pm, m | Middle | p-c, p-e, m* | 5.7±1.6 | 0.7±0.2 | 25/26 | 7.9±3.4 | 14.3±6.7 |
| Wan L et al (2015) | Article | RCSS | China | TTDC | 95 | pm, m, dcs | Middle | NA | 3.0-8.0 | NA | 33/62 | NA | 3-22 |
| Xing Q et al (2011) | Article | RCSS | China | TTDC | 32 | pm, m | Middle | p-c, p-e, m, pda | NA | NA | NA | NA | 18.3±9.6 |
| Zhang GC et al (2012) | Article | PCSS | China | TTDC | 18 | pm | Middle | p-c, p-e | 6.49±0.99 | 1.0±0.6 | 10/8 | 10.2±3.6 | NA |
| Li F et al (2008) | Article | RCSS | China | TTDC | 11 | pm,dcs | Middle | p-c, p-e | 4.59±0.97 | 14.64±8.95 | 7/4 | NA | NA |
| Zeng XJ et al (2008) | Article | RCSS | China | TTDC | 12 | pm | Middle | NA | 6.08±1.16 | 20.42±6.50 | 9/3 | 50.83±1.00 | NA |
| Crossland DS et al (2008) | Article | RCSS | Australia | TTDC | 8 | m | Middle | NA | 7.38±1.83 | 0.32±0.23 | NA | 4.53±1.3 | 5.25±6.39 |
| Xing Q et al (2010) | Article | PCSS | China | TTDC | 408 | pm | Middle | p-c, p-e | 5.3±1.6 | 3.1±1.7 | 195/213 | 13.6±5.5 | 14.6±6.2 |
| Bacha EA et al (2005) | Article | PCSS | USA | TTDC | 12 | NA | Middle | NA | NA | 1.39±1.50 | NA | 8.16±5.66 | 14.17±5.17 |
| Pan S et al (2012) | Article | RCSS | China | TTDC | 6 | dcs | Left | NA | 4.17±1.17 | 2.3±0.8 | 4/2 | 16.17±4.12 | NA |
| Bacha EA et al (2003) | Article | PCSS | USA | TTDC | 6 | NA | Middle | NA | NA | 1.06±1.33 | NA | 8.17±6.74 | 4.33±1.86 |
| Xu HS et al (2013) | Article | RCSS | China | TTDC | 235 | pm,dcs | Middle | p-c, p-e | NA | NA | NA | NA | NA |
| Gan C et al (2008) | Article | PCSS | China | TTDC | 30 | pm, dcs | Middle | p-c, p-e | 6.1±1.7 | 29.2±7.3 | 14/16 | 12.0±1.8 | NA |
| Ye JJ et al (2009) | Article | RCSS | China | TTDC | 7 | pm, m | Right | NA | 3-10 | 0.08-10.00 | NA | 3.2-35.0 | NA |
| Wang S et al (2014) | Article | RCSS | China | TTDC | 61 | pm | Middle | p-c, p-e | 4.2 (2.5-7) | 2.1±1.3 | 33/28 | 12.1±9.3 | 14.6±5.2 |
| Cao H et al (2016) | Article | RCSS | China | TTDC | 81 | dcs | Middle | NA | 6.4±2.5 | 10.5±9.6 | 43/38 | 22.5±15.1 | 30±16.8 |
| Liu HY et al (2015) | Article | RCSS | China | TTDC | 891 | pm | Middle | p-c, p-e | NA | NA | 346/545 | NA | NA |
| Zheng BR et al (2012) | Article | RCSS | China | TTDC | 39 | pm, m | Middle | p-c, p-e | 3-11 | 2.8±0.8 | 26/13 | 15.71±1.98 | 18.3±6.6 |
| Li XB et al (2012) | Article | RCSS | China | TTDC | 51 | pm | Middle | NA | NA | 0.7±0.2 | NA | 7.4±2.1 | 3.6±1.4 |
| Chen JX et al (2012) | Article | RCSS | China | TTDC | 142 | pm, m | Middle | p-c, p-e, m | NA | 3.8±1.9 | 55/67 | 16.7±9.4 | NA |
| Wang QM et al (2011) | Article | RCSS | China | TTDC | 45 | NA | Middle | NA | 4-8 | 0.58±0.38 | 18/27 | 9±3.5 | 3-20 |
| Wang EW et al (2011) | Article | RCSS | China | TTDC | 188 | pm, m | Middle, Left | p-e, m | 5.2±1.5 | 5.2±2.7 | 84/104 | 12.2±3.7 | NA |
| Li W et al (2016) | Article | RCSS | China | TTDC | 205 | pm,m | Middle, Left | NA | 5.3±1.6 | 10.2±12.5 | 93/112 | NA | NA |
| Zhao L et al (2014) | Article | RCSS | China | TTDC | 50 | pm, m, dcs | Middle | p-c, p-e, m | 3.6-9.5 | 2.5-38 | 22/28 | 11.0-66.0 | NA |
| Song B et al (2014) | Article | RCSS | China | TTDC | 39 | m | Middle | NA | NA | 0.83-3 | 13/26 | 6-15 | NA |
| Liang WJ et al (2015) | Article | RCSS | China | TTDC | 40 | pm | Right | NA | 4.7±0.8 | 2.5±1.4 | 17/23 | 9.5±4.2 | NA |
| Jing H et al (2015) | Article | RCSS | China | TTDC | 9 | dcs | Left | p-e | 4.78±1.56 | 6.22±4.71 | 4/5 | NA | NA |
| Sun Y et al (2015) | Article | RCSS | China | TTDC | 41 | pm, m, dcs | Middle | p-c, p-e | 5.2±1.13 | 1.26±1.54 | 16/25 | 10.78±6.87 | NA |
| Xu WZ et al (2011) | Article | RCSS | China | TTDC | 21 | dcs | Middle | p-e | 4.6±0.9 | 4.04±3.12 | 11/10 | 15.97±7.35 | NA |
| Liu ZH et al (2009) | Article | RCSS | China | TTDC | 19 | pm, m, dcs | Middle, Left | p-c, m, p-e | 5.49±2.90 | 8.31±7.82 | 9/10 | NA | NA |
| Shi MT (2013) | Article | RCSS | China | TTDC | 106 | pm, m, dcs | Middle, Left | p-c, m, p-e | 2.5-12 | 4-28 | 52/54 | NA | NA |
| Wang QM et al (2014) | Article | RCSS | China | TTDC | 103 | pm, m, dcs | Middle | p-c, p-e, m | 4-10 | 0.65±0.18 | 39/64 | 8.0±3.6 | NA |
| Zhu XM et al (2013) | Article | RCSS | China | TTDC | 48 | pm, m, dcs | Middle | NA | 3-10 | 2.5-43 | 20/28 | 10-65 | 32.5 (1-48) |
| Cao XX et al (2015) | Article | RCSS | China | TTDC | 425 | pm, m, dcs | Middle | NA | NA | 0.5-47 | 199/226 | NA | 12 |
| Hu L (2013) | Article | RCSS | China | TTDC | 21 | NA | Middle | p-c, p-e, m | 5.8±1.9 | 15.4±1.9 | 9/12 | 42.8±1.7 | 3-6 |
| Li Y et al (2011) | Article | RCSS | China | TTDC | 49 | pm | Middle | p-c, p-e | 5.5±1.7 | 1.92±0.56 | NA | 13.3±5.7 | 17 (6-36) |
| Li Y et al (2011) | Article | RCSS | China | TTDC | 56 | pm, m | Middle | NA | 6 (3-11) | 9.2 (10-40) | 36/20 | 23.8 (9-65) | 3-6 |
| Hu ZK et al (2014) | Article | RCSS | China | TTDC | 26 | pm, m | Middle | NA | 4.3±2.0 | 6±3 | 12/14 | 20±8 | NA |
| Lin XB et al (2014) | Article | RCSS | China | TTDC | 62 | pm, m, dcs | Middle, Left | p-c, p-e | NA | 2-57 | 34/28 | NA | 12 |
| Zhenf Y et al (2011) | Article | RCSS | China | TTDC | 21 | NA | Middle, Left | NA | 5-11 | 2.5±0.5 | 8/13 | 15±5 | NA |
| Guo JX et al (2013) | Article | RCSS | China | TTDC | 38 | pm,m, dcs | Middle | NA | 3-8 | 2.6- 13.0 | 18/20 | 10-35 | 10-24 |
| Zhang YF et al (2014) | Article | RCSS | China | TTDC | 50 | pm,m | Middle, Left | p-c, p-e | 3.9±1.6 | 3.9 (0.6-24) | NA | 16.2 (7-80) | 1-9 |
| Lu GL et al (2015) | Article | RCSS | China | TTDC | 142 | pm,m, dcs | Middle, Left | p-c, p-e, m, pda | 5.1±1.9 | 4.4±5.3 | 70/72 | 16.4±12.3 | 3-12 |
| Guo JX (2012) | Article | RCSS | China | TTDC | 20 | pm,m, dcs | Middle | NA | 3-8 | 2.6-13 | 8/12 | 10-35 | NA |
| Zhang YZ et al (2012) | Article | RCSS | China | TTDC | 85 | pm,m, dcs | Middle | p-c, p-e, m, A4B2 | 6±4 | 9.2±10.2 | 48/37 | 27±20 | 3-39 |
| Qiao JJ et al (2015) | Article | RCSS | China | TTDC | 118 | pm, dcs | Middle | NA | 4-8 | 0.5-13.0 | 46/72 | 6.0-35.0 | 3-22 |
| Song Y et al (2015) | Article | RCSS | China | TTDC | 50 | NA | Middle | NA | NA | 1.96±0.89 | 22/28 | 11.89±3.78 | 24.11±6.42 |
| Zheng BR et al (2013) | Article | RCSS | China | TTDC | 18 | NA | Middle | NA | NA | 1.08±0.43 | 13/5 | 10.15±2.69 | 5-29 |
| Qi JR et al (2009) | Article | RCSS | China | TTDC | 18 | pm,m | Middle | p-e, m, p-c | 5.5 (3.5-10.0) | 2.6 (1-6) | 9(9/18) | 10-20 | 12 (6 cases <12) |
| Xing QS et al (2011) | Article | RCSS | China | TTDC | 432 | pm, m | Middle, Left | p-c, m, p-e | 5.3±1.6 | 3.2±1.9 | 197/432 | 13.3±5.6 | 19.3±11.6 |
| Xing QS et al (2010) | Article | RCSS | China | TTDC | 136 | pm | Middle, Left | p-c, p-e | 5.5±3.1 | 1.8±1.4 | 62/74 | 12.7±7.3 | 18.3±6.6 |
| Yin SL et al (2014) | Article | PCSS | China | TTDC | 39 | dcs | Middle, Left | NA | 4.6±2.2 | 5.9±3.2 | 21/18 | NA | 7±2 |
| Zhang GC et al (2012) | Article | RCSS | China | TTDC | 46 | pm | Middle | NA | 5.55±1.01 | 2.06±2.38 | 24/22 | 11.78±5.96 | NA |
| Li SZ et al (2014) | Article | RCSS | China | TTDC | 18 | NA | Middle | p-c, p-e, m | 8±4 | 1.8±1.2 | 11/7 | NA | 3 |
| Zeng XJ et al (2009) | Article | RCSS | China | TTDC | 41 | pm | Middle | p-c, p-e | 3-8.5 | 18.7±4.9 | 27/14 | 46.3±16.1 | 7.9±4.1 |
| Hou YB et al (2015) | Article | RCSS | China | TTDC | 11 | pm, m | Middle, Left | NA | 3-8 | 3-6 | 4/7 | 12-65 | 1-6 |
| Zhu XL et al (2014) | Article | RCSS | China | TTDC | 21 | NA | Middle | NA | 4.5±2.5 | 1.3±0.7 | 11/10 | 9±3 | 3-6 |
| Yang CY et al (2014) | Article | RCSS | China | TTDC | 16 | NA | Middle | NA | NA | 1-3 | 7/9 | NA | NA |
| Zhang W et al (2011) | Article | RCSS | China | TTDC | 18 | pm, m | Middle | p-c, p-e, m | 3-10 | 8.65±14.16 | NA | NA | 1-10 |
| Liu HY et al (2013) | Article | RCSS | China | TTDC | 129 | pm | Middle | p-c, p-e | 5.00±2.45 | 0.75-57 | 69/60 | 22.06±16.15 | 10.34±2.08 |
| Wang SM et al (2013) | Article | RCSS | China | TTDC | 48 | pm | Middle | p-c, p-e | 2-9 | 2.95±2.14 | 21/27 | 13.4±8.2 | 1-6 |
| Lin Y et al (2010) | Article | RCSS | China | TTDC | 38 | NA | NA | NA | NA | 19 (2.5-36.0) | 25/13 | NA | 12 |
| Wang H et al (2012) | Article | RCSS | China | TTDC | 22 | NA | Middle | p-e | 4-12 | 2.5 (0.92-14) | 5/17 | 12.8±3.4 | 36 |
| Xu HS et al (2013) | Article | RCSS | China | TTDC | 42 | pm, m, spe | Left | NA | 3-14 | 1-58 | 24/18 | 9-70 | NA |
| Wan LZ et al (2010) | Article | RCSS | China | TTDC | 45 | pm, m | Middle | p-c, p-e, m | 4.5 (3-10) | 3.5 (1.17-11) | 18/27 | 16.8 (9-30) | 3-6 |
| Xi DW et al (2015) | Article | RCSS | China | TTDC | 142 | pm, m, dcs | NA | p-c, p-e, m | 2-10 | 0.67-11 | 52/90 | 7-35 | 3-24 |
| Yu CZ et al (2012) | Meeting abstract | RCSS | China | TTDC | 42 | pm, m, dcs | Middle | p-c, p-e, m | 2.5-8 | 0.5-9 | 14/28 | 7-29 | 3-24 |
| Li JH et al (2012) | Meeting abstract | RCSS | China | TTDC | 52 | dcs | NA | NA | 4.6±0.9 | 4.04±3.12 | 19/33 | NA | 6 |
| Liu HY et al (2012) | Meeting abstract | RCSS | China | TTDC | 429 | NA | NA | p-c, p-e, m | NA | NA | NA | NA | 1-38 |
| Li F et al (2007) | Meeting abstract | RCSS | China | TTDC | 11 | NA | Middle | NA | 3 -6 | 14.6 (2-25) | 7/4 | NA | NA |
| Xing QS et al (2011) | Meeting abstract | RCSS | China | TTDC | 121 | pm, m, dcs | Middle, Left | p-c, p-e, m, pda, sp1 | NA | 1.6±1.3 | NA | 8.6±6.8 | 26.8±12.5 |
| Xing QS et al (2007) | Meeting abstract | RCSS | China | TTDC | 36 | pm, m | Middle | p-c, p-e, m | 2-12 | 3.5 (0.92-13) | 13/23 | 16.1±5.8 | ≥4 |
| Hu JW (2015) | Masters' thesis | RCSS | China | TTDC | 10 | NA | Middle | NA | 5.4±1.35 | NA | NA | NA | NA |
| Guo WB (2010) | Meeting abstract | RCSS | China | TTDC | 62 | pm, m | NA | p-c, p-e, A4B2 | 6.2±2.5 | NA | NA | NA | 1-12 |
| Mao ZG et al (2016) | Article | RCSS | China | TTDC | 10 | pm, m | Middle | p-c, p-e, m | 5.85±1.90 | 1.65±0.76 | 4/6 | 10.80±3.00 | NA |
| Ma LC et al (2016) | Article | RCSS | China | TTDC | 15 | pm, m, dcs | Left | NA | 4.0±1.2 | 4.5±4.6 y | 8/7 | 14.6±14.1 | NA |
| Zhu D et al (2015) | Article | RCSS | China | TTDC | 35 | m, spe (ia) | Middle | NA | NA | 3.9 (0.08-7) | 19/16 | 14.1±5.6 | 15 |
| Yang SH et al (2015) | Article | RCSS | China | TTDC | 131 | pm, m | Middle | p-c, p-e | 4.10±1.37 | 2.94±2.44 | 68/63 | 14.56±7.47 | 1-12 |
| Shen S et al (2013) | Article | RCSS | China | TTDC | 18 | m | Middle | NA | 6.6±1.8 | 10.8±6.7 | 7/11 | 7.4±2.2 | 3-12 |
| Cao Y et al (2015) | Article | RCSS | China | TTDC | 23 | pm, m | Middle | NA | NA | 0.67±0.33 | 14/9 | 6.0±2.0 | 3-24 |
| MUHOOZI RWAKARYEBE MBAGGA MD (2014) | Masters' thesis | RCSS | China | TTDC | 45 | dcs | Left | p-c, p-e | 4.1±1.8 | 3.4±2.4 | 15/30 | 15.46±6.93 | 10-30 |
| Yu CJ et al (2013) | Article | RCSS | China | TTDC | 81 | pm,dcs | Middle | p-e | 4.6±0.9 | 2.36±1.44 | 33/48 | 13.97±5.25 | 12-36 |
| Wu Q et al (2013) | Article | RCSS | China | TTDC | 179 | pm, dcs | Middle | p-e | NA | 4.41±3.02 | 71/108 | 17.46±9.61 | NA |
| Lin ZB et al (2013) | Article | RCSS | China | TTDC | 22 | pm, m, dcs | Middle | p-e | 4.34±0.94 | 2-8 | 12/10 | 9.5-38 | NA |
| Wan L et al (2013) | Article | RCSS | China | TTDC | 44 | NA | Middle | p-e | 4.0-6.0 | 4.0±1.2 | 8/36 | 16.0±3.2 | NA |
| Liu J et al (2014) | Article | RCSS | China | TTDC | 63 | pm, m, dcs | Middle, Left | p-c, p-e | 1.6-10.8 | 8.99 (2-57) | 35/28 | 24.5 (10-85) | 2-47 |
| Xing QS et al (2015) | Meeting abstract | RCSS | China | TTDC | 786 | NA | NA | NA | NA | NA | NA | NA | NA |
| Ma LC et al (2016) | Article | RCSS | China | TTDC | 19 | dcs | Left | p-c, p-e | 5.4±1.8 | 3.2±4.3 | 9(9/19) | 14.3±10.9 | 14.7±7.0 |
| Dong HJ et al (2015) | Article | RCSS | China | TTDC | 15 | pm, m, dcs | Left | p-c, p-e | 5.1±2.7 | 5.3±4.2 | 7/8 | 9.8±6.4 | 3 |
| Xing QS et al (2007) | Article | RCSS | China | TTDC | 11 | pm | Middle | p-c, p-e | 6 (3-12) | 3.2 (0.08-12) | 3/8 | 15.8±6.4 | 5 |
| Zhu XM et al (2013) | Article | RCSS | China | TTDC | 45 | pm, m, dcs | Middle | NA | 3-10 | 2.5-43 | 17/28 | 10-65 | 32.5 (1-48) |
| Wang XN et al (2012) | Article | RCSS | China | TTDC | 35 | pm, m, dcs | Middle, Left | NA | 3-12 | 0.25-6 | 14/21 | 6.0-31.0 | 1-12 |
| Liu Y et al (2016) | Article | RCSS | China | TTDC | 289 | pm | Middle | p-c, p-e | 5.30±2.88 | 5.3±2.4 | 200/89 | 12.8±6.4 | 18.3±9.4 |
| Song SB et al (2016) | Article | RCSS | China | TTDC | 122 | pm | Right | p-c | 3.8±0.8 | 2.7±2.2 | 68/54 | 13.9±6.0 | 8.3±5.0 |
| Lin K et al (2008) | Article | RCSS | China | TTDC | 39 | pm, m, dcs | Middle | p-c, p-e | 6.1±2.0 | 1.21±0.65 | 23/16 | 12.4±2.3 | NA |
| Wang SX et al (2014) | Meeting abstract | RCSS | China | TTDC | 43 | NA | Left | NA | 5.53±2.60 | 3.96±4.26 | 23/20 | 15.29±4.26 | 1-12 |
| Wang C (2013) | Masters' thesis | RCSS | China | TTDC | 186 | pm, m, dcs | NA | p-c, p-e | 4.8±1.6 | NA | NA | NA | NA |
| Wang KX et al (2014) | Meeting abstract | RCSS | China | TTDC | 90 | NA | NA | NA | 4.0±1.6 | 3.4±5.5 | 44/46 | 15.2±14.9 | 1-14 |
| Liang F (2014) | Meeting abstract | RCSS | China | TTDC | 111 | NA | Middle, Left, Right | NA | 3.5-11.5 | 0.33-1 | NA | 6-13 | NA |
| Bian H et al (2012) | Article | RCSS | China | TTDC | 15 | NA | Middle | NA | 5-11 | 4 (1.5-10) | 8/7 | 12±5 | NA |
| Xing QS (2013) | Meeting abstract | RCSS | China | TTDC | 615 | pm, m, dcs | Middle, Left | p-c, p-e, sp1 | 5.3±4.6 | 4.3±8.5 | 288/327 | 16.5±12.8 | 49.6±15.6 |
| Xing QS et al (2008) | Article | RCSS | China | TTDC | 63 | pm, m | Middle, Left | p-c, p-e | 2-12 | 3.5±2.6 | 23/40 | 14.1±5.8 | 1-7 |
| Peng Q et al (2014) | Article | RCSS | China | TTDC | 85 | pm | Middle | NA | 3-10.5 | 19.8±4.8 | 47/38 | 42.3±16.5 | 8.3±5.1 |
| Zhang YZ et al (2013) | Article | RCSS | China | TTDC | 49 | m | Left, Right | p-c, p-e, m | 3.7±1.7 | 0.3-53.0 | 16/33 | 6-84 | 3-24 |
| Wang H et al (2016) | Article | RCSS | China | TTDC | 281 | pm | Middle | NA | 4-10 | NA | NA | NA | NA |
| Wu Q et al (2012) | Article | RCSS | China | TTDC | 446 | NA | Middle | p-c, p-e, m, pda | 4.33±1.78 | 4.6±2.8 | 210/236 | 16.9±6.3 | 1-12 |
| Wang SM et al (2012) | Article | RCSS | China | TTDC | 45 | NA | Middle | NA | 2-9 | 2.84±2.02 | 20/25 | 12.4±9.2 | 1-6 |
| Lin K et al (2013) | Article | PCSS | China | TTDC | 34 | dcs | Middle, Left | p-e | 4.9±1.6 | 7.9±5.8 | 15/19 | 25.3±15.4 | 20 (10-30) |
| Haponiuk I et al (2013) | Article | RCSS | Poland | TTDC | 10 | m | Middle | AO-m, AO-pda | 6.3 (4-10) | 8.5 (2.7-17.8) | NA | 6.34 (3.4-7.5) | 18 (2-38) |
| Liu L et al (2013) | Article | RCSS | China | TTDC | 62 | pm, dcs | Middle, Left | NA | pm7.5±1.8; dcs 5.4±1.2 | 4.49±2.92 | 25/37 | 16.47±5.97 | 22.3±5.2 |
| Bendaly EA et al (2011) | Article | RCSS | USA | TTDC | 6 | m | Middle | NA | NA | 0.82±0.76 | NA | 7.2±3.7 | 39.8±25.2 |
| Pedra CAC et al (2009) | Article | RCSS | Brasil | TTDC | 9 | NA | Middle | NA | NA | 0.73±0.63 | NA | 6.51±2.61 | 1 (0.25-2) |
| Fouilloux V et al (2012) | Article | RCSS | France | TTDC | 8 | m | Middle | NA | 6.78±2.82 | 0.31±0.22 | 4/4 | 4.15±1.23 | 34.86±17.28 |
| Thakkar B et al (2012) | Article | RCSS | India | TTDC | 24 | m | Middle | m | 8.42±1.46 | 0.50±0.20 | 14/10 | 4.27±0.56 | 26.23±6.63 |
| Gan C et al (2009) | Article | RCSS | China | TTDC | 8 | m | Middle | m, pda | 4.9±2.4 | 2.5±3.6 | 4/4 | 10.9±7.6 | NA |
| Tao K et al (2010) | Article | RCSS | China | TTDC | 61 | pm | Middle | p-c, p-e | 5.3±1.6 | 2.87±1.21 | 34/27 | 12.6±3.2 | 22 (16-28) |
| Kim SJ et al (2013) | Article | RCSS | South Korea | TTDC | 5 | m | Middle | AO-pda | 6.4±0.89 | 0.82±0.90 | 3/2 | 9.1±4.67 | 28.8 |
| Lim DS et al (2007) | Article | PCSS | USA | TTDC | 5 | m | NA | NA | NA | NA | NA | NA | NA |
| Gao Z et al (2014) | Article | PCSS | China | TTDC | 9 | m | Middle | NA | 12±4 | 0.66±0.12 | 3/6 | 7.38±0.95 | 4-14 |
| Zhu D et al (2013) | Article | RCSS | China | TTDC | 31 | pm | Middle | p-c, p-e | 5.8±1.7 | 2.1±0.9 | NA | 12.7±3.4 | 14.4±2.4 |
| Chen Q et al (2012) | Article | PCSS | China | TTDC | 97 | pm | Middle | NA | 7.6±1.6 | 13.2±11.8 | 55/42 | 34.1±16.9 | 16.44±6.72 |
| Zeng W et al (2011) | Article | RCSS | China | TTDC | 6 | pm, m | Middle | p-c, p-e | 7.17±2.40 | 25.83±12.06 | 4/2 | NA | NA |
| Zhang ZW et al (2006) | Meeting abstract | CR | China | TTDC | 2 | pm, m | Middle | NA | 9, 6.9 | 7/12, 1 | 1/1 | 6,9 | NA |
| Karimi M et al (2012) | Article | CR | USA | TTDC | 1 | spe | Middle | m | 14 | 20/12 | 1/0 | NA | 2 |
| Diab KA et al (2005) | Article | CR | USA | TTDC | 1 | m | Middle | AO-pda, AO-m | 9, 7 | 4/12 | NA | 5.7 | 3 |
| Becker P et al (2004) | Article | CR | Chile | TTDC | 1 | m | Middle | AO-m | 3-6 | 1.25 | 1/0 | 8 | 6 |
| Maheshwari S et al (2004) | Article | CR | USA | TTDC | 2 | m | NA | NA | NA | 1 case <2, 1 case 2-16 | NA | NA | 3 |
| Breinholt JP et al (2009) | Article | CR | USA | TTDC | 1 | m | Middle | NA | NA | 0.125 | 0/1 | NA | 16 |
| Zhu D et al (2011) | Article | CR | China | TTDC-COHS | 1 | m | Middle | AO-m | 4, 8 | 0.5 | 1/0 | NA | 6 |
| Amin Z et al (1998) | Communication | CR | USA | TTDC | 1 | m | Middle | NA | NA | 8/12 | 1/0 | NA | NA |
| Lim DS et al (2008) | Article | CR | USA | TTDC | 1 | NA | Middle | AO-pda | NA | 50 | NA | NA | NA |
| Schreiber C et al (2013) | Article | CR | Germany | TTDC | 1 | pm | Middle | p-c | 8 | 7 | 1/0 | NA | 24 |
| Celiker A et al (2008) | Article | CR | Turkey | TTDC | 1 | m | Middle | AO-m | 4-5 | 2.5/12 | NA | 5 | NA |
| Meng W et al (2013) | Article | CR | China | TTDC | 1 | spe | Middle | p-c | 15 | 51 | 0/1 | NA | 6 |
| Wu Q et al (2010) | Communication | CR | China | TTDC | 1 | pm | Middle | p-e | 4.23 | 0.25 | 1/0 | 5 | NA |

CSS, case series study; NA, not available; SRRIAT, surgical repair with right infra-axillary thoracotomy; MTOHS, mini-thoracotomy open-heart surgery; TTDC-SBA, TTDC with solid bougie auxiliary; TTDC-HBA, TTDC with hollow bougie auxiliary; TTDC-DT, TTDC with direct transportation; TTDC-tee, TTDC with transesophageal echocardiography; TTDC-tte, TTDC with transthoracic echocardiography; TTDC-TI, minimally invasive periventricular device closure with thoracoscopic instruments; TTDC-COHS, unique hybrid procedure combining both the perventricular closure and open surgical repair;

pm, perimembranous VSD; m, muscular VSD; dcs, doubly committed subarterial VSD; icVSD, intracristal VSD; spe, the special type of VSD; ia, iatrogenic VSD; mi, myocardial infarction VSD;

p-c, a perimembranous VSD concentric occluder (concentric); p-e, a perimembranous VSD eccentric occluder (eccentric); m, a muscular VSD occluder (muscular); pda, a patent ductus arteriosus (PDA) occluder; sp1, a special occluder (saddle-like) for subarterial VSD; A4B2, A4B2 occluders (the right disk is 2 mm larger than the waist; the left is 4 mm larger); AO-m, Amplatzer mvsd occluder; AO-mi, Amplatzer miVSD occluder; AO-pm, Amplatzer pmVSD occluder; AO-a, Amplatzer ASD occluder; AO-pda, Amplatzer PDA occluder;

#, three-arm studies

*, p-e was SQFDQ IV, p-c was SQFDQ II or SQFDQ III, m was SQFDQ I

Supplemental tab B NOS for Cohort Studies

|  | **Representativeness of the exposed cohort** | **Selection of the non-exposed cohort** | **Ascertainment of exposure** | **Demonstration that outcome of interest was not present at start of study** | **Comparability of cohorts on the basis of the design or analysis^b^** | **Assessment of outcome** | **Was follow-up long enough for outcome to occur** | **Adequacy of follow-up of cohorts** | **Total quality scores** |
| --- | --- | --- | --- | --- | --- | --- | --- | --- | --- |
| Xing Q et al (2015) | * |  | * | * | ** | * | * | * | 8 |
| Chen Q et al (2013) | * | * | * | * | ** | * | * | * | 9 |
| Xu F et al (2012) | * | * | * | * |  | * | * | * | 7 |
| Zhang C et al (2014) | * | * | * | * | ** | * | * | * | 9 |
| Li YJ et al (2015) | * | * | * | * | ** | * | * | * | 9 |
| Lu GL (2015) | * | * | * | * | ** | * | * | * | 9 |
| Zhang GC et al (2013) | * | * | * | * | ** | * | * | * | 9 |

a A study can be awarded a maximum of one star for each numbered item except for the item Comparability of cohorts on the basis of the design or analysis.

b A maximum of two stars can be awarded for Comparability of cohorts on the basis of the design or analysis.

Supplemental tab C. NOS for Case Control Studies

|  | **Adequate definition of case** | **Representativeness of cases** | **Selection of control** | **Definition of control** | **Control for important factor or additional factor^b^** | **Exposure assessment** | **Same method of ascertainment for cases and controls** | **Nonresponse rate** | **Total quality scores** |
| --- | --- | --- | --- | --- | --- | --- | --- | --- | --- |
| Hu Y et al (2015) | * | * | * | * | ** | * | * | * | 9 |
| Michel-Behnke I et al (2011) | * | * |  | * | ** | * | * |  | 7 |
| Zhang YZ et al (2011) | * | * | * | * | * | * | * | * | 8 |
| Chen MY et al (2013) | * | * | * | * |  | * | * | * | 7 |
| Yang XC et al (2014) | * | * | * | * | ** | * | * | * | 9 |
| Jiang MZ (2014) | * | * | * | * | ** | * | * | * | 9 |
| Xu F et al (2012) | * | * | * | * | ** | * | * | * | 9 |
| Fang S (2015) | * | * | * | * | ** | * | * | * | 9 |
| Zou GW (2015) | * | * | * | * | ** | * | * | * | 9 |
| Yao Y et al (2013) | * | * | * | * | ** | * | * | * | 9 |
| Zhu D et al (2014) | * | * |  | * | ** | * | * |  | 7 |
| Hu S et al (2014) | * | * | * | * | ** | * | * | * | 9 |
| Li HX et al (2011) | * | * | * | * |  | * |  | * | 6 |

a A study can be awarded a maximum of one star for each numbered item except for the item Control for important factor or additional factor.

b A maximum of two stars can be awarded for Control for important factor or additional factor.

Supplemental tab D. Quality Appraisal for Case Series

|  | Bai W et al (2012) | | Yang Y et al (2014) | | | Ou-Yang WB et al (2015) | | | | Wu Q et al (2012) | | | Chen Q et al (2010) | | | | Cao H et al (2011) | | | |  | | |
| --- | --- | --- | --- | --- | --- | --- | --- | --- | --- | --- | --- | --- | --- | --- | --- | --- | --- | --- | --- | --- | --- | --- | --- |
| Study Objective |  | |  | | |  | | | |  | | |  | | | |  | | | |  | | |
| 1. Is the hypothesis/aim/objective of the study stated clearly in the abstract, introduction, or methods section? | 1 | | 1 | | | 1 | | | | 1 | | | 1 | | | | 1 | | | |  | | |
| Study Population |  | |  | | |  | | | |  | | |  | | | |  | | | |  | | |
| 2. Are the characteristics of the participants included in the study described? | 1 | | 1 | | | 1 | | | | 1 | | | 1 | | | | 1 | | | |  | | |
| 3. Were the cases collected in more than one centre? | 0 | | 0 | | | 0 | | | | 1 | | | 0 | | | | 0 | | | |  | | |
| 4. Are the eligibility criteria (inclusion and exclusion criteria) for entry into the study explicit and appropriate? | 1 | | 1 | | | 1 | | | | 0 | | | 0 | | | | 1 | | | |  | | |
| 5. Were participants recruited consecutively? | 1 | | 1 | | | 1 | | | | 1 | | | 1 | | | | 1 | | | |  | | |
| 6. Did participants enter the study at a similar point in the disease? | 1 | | 1 | | | 1 | | | | 1 | | | 1 | | | | 1 | | | |  | | |
| Intervention and co-intervention |  | |  | | |  | | | |  | | |  | | | |  | | | |  | | |
| 7. Was the intervention clearly described in the study? | 1 | | 1 | | | 1 | | | | 1 | | | 1 | | | | 1 | | | |  | | |
| 8. Were additional interventions (cointerventions) clearly reported in the study? | 1 | | 1 | | | 1 | | | | 1 | | | 1 | | | | 1 | | | |  | | |
| Outcome measures |  | |  | | |  | | | |  | | |  | | | |  | | | |  | | |
| 9. Are the outcome measures clearly defined in the introduction or methods section? | 1 | | 1 | | | 1 | | | | 1 | | | 1 | | | | 1 | | | |  | | |
| 10. Were relevant outcomes appropriately measured with objective and/or subjective methods? | 1 | | 1 | | | 1 | | | | 1 | | | 1 | | | | 1 | | | |  | | |
| 11. Were outcomes measured before and after intervention? | 1 | | 1 | | | 1 | | | | 1 | | | 1 | | | | 1 | | | |  | | |
| Statistical analysis |  | |  | | |  | | | |  | | |  | | | |  | | | |  | | |
| 12. Were the statistical tests used to assess the relevant outcomes appropriate? | 0 | | 0 | | | 0 | | | | 0 | | | 0 | | | | 0 | | | |  | | |
| Results and conclusions |  | |  | | |  | | | |  | | |  | | | |  | | | |  | | |
| 13. Was the length of follow-up reported? | 1 | | 1 | | | 1 | | | | 1 | | | 1 | | | | 1 | | | |  | | |
| 14. Was the loss to follow-up reported? | 0 | | 0 | | | 1 | | | | 1 | | | 0 | | | | 0 | | | |  | | |
| 15. Does the study provide estimates of the random variability in the data analysis of relevant outcomes? | 1 | | 1 | | | 1 | | | | 1 | | | 1 | | | | 1 | | | |  | | |
| 16. Are adverse events reported? | 1 | | 1 | | | 1 | | | | 1 | | | 1 | | | | 1 | | | |  | | |
| 17. Are the conclusions of the study supported by results? | 1 | | 1 | | | 1 | | | | 1 | | | 1 | | | | 1 | | | |  | | |
| Competing interests and sources of support |  | |  | | |  | | | |  | | |  | | | |  | | | |  | | |
| 18. Are both competing interests and sources of support for the study reported? | 0 | | 0 | | | 0 | | | | 0 | | | 0 | | | | 0 | | | |  | | |
| Total score | 14 | | 14 | | | 15 | | | | 15 | | | 13 | | | | 14 | | | |  | | |
|  | Quansheng X et al (2009) | | | Omelchenko A et al (2016) | | | | Wan L et al (2015) | | | | Xing Q et al (2011) | | | Zhang GC et al (2012) | | | | Li F et al (2008) | | | |  |
| Study Objective |  | | |  | | | |  | | | |  | | |  | | | |  | | | |  |
| 1. Is the hypothesis/aim/objective of the study stated clearly in the abstract, introduction, or methods section? | 1 | | | 1 | | | | 1 | | | | 1 | | | 1 | | | | 1 | | | |  |
| Study Population |  | | |  | | | |  | | | |  | | |  | | | |  | | | |  |
| 2. Are the characteristics of the participants included in the study described? | 1 | | | 1 | | | | 1 | | | | 1 | | | 1 | | | | 1 | | | |  |
| 3. Were the cases collected in more than one centre? | 0 | | | 0 | | | | 0 | | | | 0 | | | 0 | | | | 0 | | | |  |
| 4. Are the eligibility criteria (inclusion and exclusion criteria) for entry into the study explicit and appropriate? | 0 | | | 1 | | | | 0 | | | | 1 | | | 1 | | | | 0 | | | |  |
| 5. Were participants recruited consecutively? | 1 | | | 1 | | | | 1 | | | | 1 | | | 1 | | | | 1 | | | |  |
| 6. Did participants enter the study at a similar point in the disease? | 1 | | | 1 | | | | 1 | | | | 1 | | | 1 | | | | 1 | | | |  |
| Intervention and co-intervention |  | | |  | | | |  | | | |  | | |  | | | |  | | | |  |
| 7. Was the intervention clearly described in the study? | 1 | | | 1 | | | | 1 | | | | 1 | | | 1 | | | | 1 | | | |  |
| 8. Were additional interventions (cointerventions) clearly reported in the study? | 1 | | | 1 | | | | 1 | | | | 1 | | | 1 | | | | 1 | | | |  |
| Outcome measures |  | | |  | | | |  | | | |  | | |  | | | |  | | | |  |
| 9. Are the outcome measures clearly defined in the introduction or methods section? | 1 | | | 1 | | | | 0 | | | | 1 | | | 1 | | | | 1 | | | |  |
| 10. Were relevant outcomes appropriately measured with objective and/or subjective methods? | 1 | | | 1 | | | | 1 | | | | 1 | | | 1 | | | | 1 | | | |  |
| 11. Were outcomes measured before and after intervention? | 1 | | | 1 | | | | 1 | | | | 1 | | | 1 | | | | 1 | | | |  |
| Statistical analysis |  | | |  | | | |  | | | |  | | |  | | | |  | | | |  |
| 12. Were the statistical tests used to assess the relevant outcomes appropriate? | 0 | | | 0 | | | | 0 | | | | 0 | | | 0 | | | | 0 | | | |  |
| Results and conclusions |  | | |  | | | |  | | | |  | | |  | | | |  | | | |  |
| 13. Was the length of follow-up reported? | 1 | | | 1 | | | | 1 | | | | 1 | | | 0 | | | | 1 | | | |  |
| 14. Was the loss to follow-up reported? | 0 | | | 0 | | | | 0 | | | | 1 | | | 0 | | | | 1 | | | |  |
| 15. Does the study provide estimates of the random variability in the data analysis of relevant outcomes? | 0 | | | 1 | | | | 1 | | | | 1 | | | 0 | | | | 1 | | | |  |
| 16. Are adverse events reported? | 1 | | | 1 | | | | 1 | | | | 1 | | | 1 | | | | 1 | | | |  |
| 17. Are the conclusions of the study supported by results? | 1 | | | 1 | | | | 1 | | | | 1 | | | 1 | | | | 1 | | | |  |
| Competing interests and sources of support |  | | |  | | | |  | | | |  | | |  | | | |  | | | |  |
| 18. Are both competing interests and sources of support for the study reported? | 0 | | | 0 | | | | 0 | | | | 0 | | | 0 | | | | 0 | | | |  |
| Total score | 12 | | | 14 | | | | 12 | | | | 15 | | | 12 | | | | 14 | | | |  |
|  | Zeng XJ et al (2008) | | Crossland DS et al (2008) | | | | Xing Q et al (2010) | | | | Bacha EA et al (2005) | | | Pan S et al (2012) | | | | Bacha EA et al (2003) | | | |  | |
| Study Objective |  | |  | | | |  | | | |  | | |  | | | |  | | | |  | |
| 1. Is the hypothesis/aim/objective of the study stated clearly in the abstract, introduction, or methods section? | 1 | | 1 | | | | 1 | | | | 1 | | | 1 | | | | 1 | | | |  | |
| Study Population |  | |  | | | |  | | | |  | | |  | | | |  | | | |  | |
| 2. Are the characteristics of the participants included in the study described? | 1 | | 1 | | | | 1 | | | | 1 | | | 1 | | | | 1 | | | |  | |
| 3. Were the cases collected in more than one centre? | 0 | | 0 | | | | 0 | | | | 0 | | | 0 | | | | 0 | | | |  | |
| 4. Are the eligibility criteria (inclusion and exclusion criteria) for entry into the study explicit and appropriate? | 0 | | 1 | | | | 1 | | | | 1 | | | 1 | | | | 1 | | | |  | |
| 5. Were participants recruited consecutively? | 1 | | 1 | | | | 1 | | | | 1 | | | 1 | | | | 1 | | | |  | |
| 6. Did participants enter the study at a similar point in the disease? | 1 | | 1 | | | | 1 | | | | 1 | | | 1 | | | | 1 | | | |  | |
| Intervention and co-intervention |  | |  | | | |  | | | |  | | |  | | | |  | | | |  | |
| 7. Was the intervention clearly described in the study? | 1 | | 1 | | | | 1 | | | | 1 | | | 1 | | | | 1 | | | |  | |
| 8. Were additional interventions (cointerventions) clearly reported in the study? | 1 | | 1 | | | | 1 | | | | 1 | | | 1 | | | | 1 | | | |  | |
| Outcome measures |  | |  | | | |  | | | |  | | |  | | | |  | | | |  | |
| 9. Are the outcome measures clearly defined in the introduction or methods section? | 1 | | 1 | | | | 1 | | | | 1 | | | 1 | | | | 1 | | | |  | |
| 10. Were relevant outcomes appropriately measured with objective and/or subjective methods? | 1 | | 1 | | | | 1 | | | | 1 | | | 1 | | | | 1 | | | |  | |
| 11. Were outcomes measured before and after intervention? | 1 | | 1 | | | | 1 | | | | 1 | | | 1 | | | | 1 | | | |  | |
| Statistical analysis |  | |  | | | |  | | | |  | | |  | | | |  | | | |  | |
| 12. Were the statistical tests used to assess the relevant outcomes appropriate? | 0 | | 0 | | | | 0 | | | | 0 | | | 0 | | | | 0 | | | |  | |
| Results and conclusions |  | |  | | | |  | | | |  | | |  | | | |  | | | |  | |
| 13. Was the length of follow-up reported? | 1 | | 1 | | | | 1 | | | | 1 | | | 1 | | | | 1 | | | |  | |
| 14. Was the loss to follow-up reported? | 0 | | 1 | | | | 1 | | | | 0 | | | 0 | | | | 1 | | | |  | |
| 15. Does the study provide estimates of the random variability in the data analysis of relevant outcomes? | 0 | | 0 | | | | 1 | | | | 0 | | | 0 | | | | 0 | | | |  | |
| 16. Are adverse events reported? | 0 | | 1 | | | | 1 | | | | 1 | | | 1 | | | | 1 | | | |  | |
| 17. Are the conclusions of the study supported by results? | 1 | | 1 | | | | 1 | | | | 1 | | | 1 | | | | 1 | | | |  | |
| Competing interests and sources of support |  | |  | | | |  | | | |  | | |  | | | |  | | | |  | |
| 18. Are both competing interests and sources of support for the study reported? | 0 | | 0 | | | | 0 | | | | 0 | | | 0 | | | | 0 | | | |  | |
| Total score | 11 | | 14 | | | | 15 | | | | 13 | | | 13 | | | | 14 | | | |  | |
|  | Xu HS et al (2013) | | Gan C et al (2008) | | | Ye JJ et al (2009) | | | | Wang S et al (2014) | | | Cao H et al (2016) | | | | Liu HY et al (2015) | | | |  | | |
| Study Objective |  | |  | | |  | | | |  | | |  | | | |  | | | |  | | |
| 1. Is the hypothesis/aim/objective of the study stated clearly in the abstract, introduction, or methods section? | 1 | | 1 | | | 1 | | | | 1 | | | 1 | | | | 1 | | | |  | | |
| Study Population |  | |  | | |  | | | |  | | |  | | | |  | | | |  | | |
| 2. Are the characteristics of the participants included in the study described? | 0 | | 1 | | | 0 | | | | 1 | | | 1 | | | | 1 | | | |  | | |
| 3. Were the cases collected in more than one centre? | 0 | | 0 | | | 0 | | | | 0 | | | 0 | | | | 0 | | | |  | | |
| 4. Are the eligibility criteria (inclusion and exclusion criteria) for entry into the study explicit and appropriate? | 1 | | 1 | | | 0 | | | | 1 | | | 1 | | | | 0 | | | |  | | |
| 5. Were participants recruited consecutively? | 1 | | 1 | | | 1 | | | | 1 | | | 1 | | | | 1 | | | |  | | |
| 6. Did participants enter the study at a similar point in the disease? | 1 | | 1 | | | 1 | | | | 1 | | | 1 | | | | 1 | | | |  | | |
| Intervention and co-intervention |  | |  | | |  | | | |  | | |  | | | |  | | | |  | | |
| 7. Was the intervention clearly described in the study? | 1 | | 1 | | | 1 | | | | 1 | | | 1 | | | | 1 | | | |  | | |
| 8. Were additional interventions (cointerventions) clearly reported in the study? | 1 | | 1 | | | 1 | | | | 1 | | | 1 | | | | 1 | | | |  | | |
| Outcome measures |  | |  | | |  | | | |  | | |  | | | |  | | | |  | | |
| 9. Are the outcome measures clearly defined in the introduction or methods section? | 1 | | 1 | | | 1 | | | | 1 | | | 1 | | | | 1 | | | |  | | |
| 10. Were relevant outcomes appropriately measured with objective and/or subjective methods? | 1 | | 1 | | | 1 | | | | 1 | | | 1 | | | | 1 | | | |  | | |
| 11. Were outcomes measured before and after intervention? | 1 | | 1 | | | 1 | | | | 1 | | | 1 | | | | 1 | | | |  | | |
| Statistical analysis |  | |  | | |  | | | |  | | |  | | | |  | | | |  | | |
| 12. Were the statistical tests used to assess the relevant outcomes appropriate? | 0 | | 1 | | | 0 | | | | 0 | | | 0 | | | | 0 | | | |  | | |
| Results and conclusions |  | |  | | |  | | | |  | | |  | | | |  | | | |  | | |
| 13. Was the length of follow-up reported? | 1 | | 1 | | | 1 | | | | 1 | | | 1 | | | | 1 | | | |  | | |
| 14. Was the loss to follow-up reported? | 1 | | 0 | | | 0 | | | | 0 | | | 1 | | | | 1 | | | |  | | |
| 15. Does the study provide estimates of the random variability in the data analysis of relevant outcomes? | 0 | | 1 | | | 0 | | | | 1 | | | 1 | | | | 1 | | | |  | | |
| 16. Are adverse events reported? | 1 | | 1 | | | 1 | | | | 1 | | | 1 | | | | 1 | | | |  | | |
| 17. Are the conclusions of the study supported by results? | 1 | | 1 | | | 1 | | | | 1 | | | 1 | | | | 1 | | | |  | | |
| Competing interests and sources of support |  | |  | | |  | | | |  | | |  | | | |  | | | |  | | |
| 18. Are both competing interests and sources of support for the study reported? | 0 | | 0 | | | 0 | | | | 0 | | | 1 | | | | 0 | | | |  | | |
| Total score | 13 | | 15 | | | 11 | | | | 14 | | | 16 | | | | 14 | | | |  | | |
|  | Zheng BR et al (2012) | | Li XB et al (2012) | | | Chen JX et al (2012) | | | | Wang QM et al (2011) | | | Wang EW et al (2011) | | | | Li W et al (2016) | | | |  | | |
| Study Objective |  | |  | | |  | | | |  | | |  | | | |  | | | |  | | |
| 1. Is the hypothesis/aim/objective of the study stated clearly in the abstract, introduction, or methods section? | 1 | | 1 | | | 1 | | | | 1 | | | 0 | | | | 1 | | | |  | | |
| Study Population |  | |  | | |  | | | |  | | |  | | | |  | | | |  | | |
| 2. Are the characteristics of the participants included in the study described? | 1 | | 1 | | | 1 | | | | 1 | | | 1 | | | | 1 | | | |  | | |
| 3. Were the cases collected in more than one centre? | 0 | | 0 | | | 1 | | | | 0 | | | 0 | | | | 0 | | | |  | | |
| 4. Are the eligibility criteria (inclusion and exclusion criteria) for entry into the study explicit and appropriate? | 0 | | 1 | | | 0 | | | | 0 | | | 0 | | | | 0 | | | |  | | |
| 5. Were participants recruited consecutively? | 1 | | 1 | | | 1 | | | | 1 | | | 1 | | | | 1 | | | |  | | |
| 6. Did participants enter the study at a similar point in the disease? | 1 | | 1 | | | 1 | | | | 1 | | | 1 | | | | 1 | | | |  | | |
| Intervention and co-intervention |  | |  | | |  | | | |  | | |  | | | |  | | | |  | | |
| 7. Was the intervention clearly described in the study? | 1 | | 1 | | | 1 | | | | 1 | | | 1 | | | | 1 | | | |  | | |
| 8. Were additional interventions (cointerventions) clearly reported in the study? | 1 | | 1 | | | 1 | | | | 1 | | | 1 | | | | 1 | | | |  | | |
| Outcome measures |  | |  | | |  | | | |  | | |  | | | |  | | | |  | | |
| 9. Are the outcome measures clearly defined in the introduction or methods section? | 1 | | 1 | | | 1 | | | | 1 | | | 0 | | | | 1 | | | |  | | |
| 10. Were relevant outcomes appropriately measured with objective and/or subjective methods? | 1 | | 1 | | | 1 | | | | 1 | | | 1 | | | | 1 | | | |  | | |
| 11. Were outcomes measured before and after intervention? | 1 | | 1 | | | 1 | | | | 1 | | | 1 | | | | 1 | | | |  | | |
| Statistical analysis |  | |  | | |  | | | |  | | |  | | | |  | | | |  | | |
| 12. Were the statistical tests used to assess the relevant outcomes appropriate? | 0 | | 0 | | | 0 | | | | 0 | | | 0 | | | | 0 | | | |  | | |
| Results and conclusions |  | |  | | |  | | | |  | | |  | | | |  | | | |  | | |
| 13. Was the length of follow-up reported? | 1 | | 1 | | | 1 | | | | 1 | | | 1 | | | | 1 | | | |  | | |
| 14. Was the loss to follow-up reported? | 1 | | 1 | | | 1 | | | | 1 | | | 0 | | | | 1 | | | |  | | |
| 15. Does the study provide estimates of the random variability in the data analysis of relevant outcomes? | 0 | | 1 | | | 1 | | | | 1 | | | 1 | | | | 1 | | | |  | | |
| 16. Are adverse events reported? | 1 | | 1 | | | 1 | | | | 1 | | | 1 | | | | 1 | | | |  | | |
| 17. Are the conclusions of the study supported by results? | 1 | | 1 | | | 1 | | | | 1 | | | 1 | | | | 1 | | | |  | | |
| Competing interests and sources of support |  | |  | | |  | | | |  | | |  | | | |  | | | |  | | |
| 18. Are both competing interests and sources of support for the study reported? | 0 | | 0 | | | 0 | | | | 0 | | | 0 | | | | 0 | | | |  | | |
| Total score | 13 | | 15 | | | 15 | | | | 14 | | | 11 | | | | 14 | | | |  | | |
|  | Zhao L et al (2014) | | Song B et al (2014) | | | Liang WJ et al (2015) | | | | Jing H et al (2015) | | | Sun Y et al (2015) | | | | Xu WZ et al (2011) | | | |  | | |
| Study Objective |  | |  | | |  | | | |  | | |  | | | |  | | | |  | | |
| 1. Is the hypothesis/aim/objective of the study stated clearly in the abstract, introduction, or methods section? | 1 | | 1 | | | 1 | | | | 1 | | | 1 | | | | 1 | | | |  | | |
| Study Population |  | |  | | |  | | | |  | | |  | | | |  | | | |  | | |
| 2. Are the characteristics of the participants included in the study described? | 1 | | 1 | | | 1 | | | | 1 | | | 1 | | | | 1 | | | |  | | |
| 3. Were the cases collected in more than one centre? | 0 | | 0 | | | 0 | | | | 0 | | | 0 | | | | 0 | | | |  | | |
| 4. Are the eligibility criteria (inclusion and exclusion criteria) for entry into the study explicit and appropriate? | 0 | | 0 | | | 1 | | | | 0 | | | 0 | | | | 0 | | | |  | | |
| 5. Were participants recruited consecutively? | 1 | | 1 | | | 1 | | | | 1 | | | 1 | | | | 1 | | | |  | | |
| 6. Did participants enter the study at a similar point in the disease? | 1 | | 1 | | | 1 | | | | 1 | | | 1 | | | | 1 | | | |  | | |
| Intervention and co-intervention |  | |  | | |  | | | |  | | |  | | | |  | | | |  | | |
| 7. Was the intervention clearly described in the study? | 1 | | 1 | | | 1 | | | | 1 | | | 1 | | | | 1 | | | |  | | |
| 8. Were additional interventions (cointerventions) clearly reported in the study? | 1 | | 1 | | | 1 | | | | 1 | | | 1 | | | | 1 | | | |  | | |
| Outcome measures |  | |  | | |  | | | |  | | |  | | | |  | | | |  | | |
| 9. Are the outcome measures clearly defined in the introduction or methods section? | 1 | | 1 | | | 1 | | | | 1 | | | 1 | | | | 1 | | | |  | | |
| 10. Were relevant outcomes appropriately measured with objective and/or subjective methods? | 1 | | 1 | | | 1 | | | | 1 | | | 1 | | | | 1 | | | |  | | |
| 11. Were outcomes measured before and after intervention? | 1 | | 1 | | | 1 | | | | 1 | | | 1 | | | | 1 | | | |  | | |
| Statistical analysis |  | |  | | |  | | | |  | | |  | | | |  | | | |  | | |
| 12. Were the statistical tests used to assess the relevant outcomes appropriate? | 0 | | 0 | | | 0 | | | | 0 | | | 0 | | | | 0 | | | |  | | |
| Results and conclusions |  | |  | | |  | | | |  | | |  | | | |  | | | |  | | |
| 13. Was the length of follow-up reported? | 1 | | 1 | | | 1 | | | | 1 | | | 1 | | | | 1 | | | |  | | |
| 14. Was the loss to follow-up reported? | 1 | | 0 | | | 0 | | | | 0 | | | 1 | | | | 0 | | | |  | | |
| 15. Does the study provide estimates of the random variability in the data analysis of relevant outcomes? | 0 | | 1 | | | 0 | | | | 1 | | | 1 | | | | 1 | | | |  | | |
| 16. Are adverse events reported? | 1 | | 1 | | | 1 | | | | 1 | | | 1 | | | | 1 | | | |  | | |
| 17. Are the conclusions of the study supported by results? | 1 | | 1 | | | 1 | | | | 1 | | | 1 | | | | 1 | | | |  | | |
| Competing interests and sources of support |  | |  | | |  | | | |  | | |  | | | |  | | | |  | | |
| 18. Are both competing interests and sources of support for the study reported? | 0 | | 0 | | | 0 | | | | 0 | | | 0 | | | | 0 | | | |  | | |
| Total score | 13 | | 13 | | | 13 | | | | 13 | | | 14 | | | | 13 | | | |  | | |
|  | Liu ZH et al (2009) | | Shi MT (2013) | | | Wang QM et al (2014) | | | | Zhu XM et al (2013) | | | Cao XX et al (2015) | | | | Hu L (2013) | | | |  | | |
| Study Objective |  | |  | | |  | | | |  | | |  | | | |  | | | |  | | |
| 1. Is the hypothesis/aim/objective of the study stated clearly in the abstract, introduction, or methods section? | 1 | | 1 | | | 0 | | | | 1 | | | 1 | | | | 1 | | | |  | | |
| Study Population |  | |  | | |  | | | |  | | |  | | | |  | | | |  | | |
| 2. Are the characteristics of the participants included in the study described? | 1 | | 1 | | | 1 | | | | 1 | | | 1 | | | | 1 | | | |  | | |
| 3. Were the cases collected in more than one centre? | 0 | | 0 | | | 0 | | | | 0 | | | 0 | | | | 0 | | | |  | | |
| 4. Are the eligibility criteria (inclusion and exclusion criteria) for entry into the study explicit and appropriate? | 0 | | 0 | | | 0 | | | | 0 | | | 0 | | | | 0 | | | |  | | |
| 5. Were participants recruited consecutively? | 1 | | 1 | | | 1 | | | | 1 | | | 1 | | | | 1 | | | |  | | |
| 6. Did participants enter the study at a similar point in the disease? | 1 | | 1 | | | 1 | | | | 1 | | | 1 | | | | 1 | | | |  | | |
| Intervention and co-intervention |  | |  | | |  | | | |  | | |  | | | |  | | | |  | | |
| 7. Was the intervention clearly described in the study? | 1 | | 1 | | | 1 | | | | 1 | | | 1 | | | | 1 | | | |  | | |
| 8. Were additional interventions (cointerventions) clearly reported in the study? | 1 | | 1 | | | 1 | | | | 1 | | | 1 | | | | 1 | | | |  | | |
| Outcome measures |  | |  | | |  | | | |  | | |  | | | |  | | | |  | | |
| 9. Are the outcome measures clearly defined in the introduction or methods section? | 1 | | 1 | | | 1 | | | | 1 | | | 1 | | | | 1 | | | |  | | |
| 10. Were relevant outcomes appropriately measured with objective and/or subjective methods? | 1 | | 1 | | | 1 | | | | 1 | | | 1 | | | | 1 | | | |  | | |
| 11. Were outcomes measured before and after intervention? | 1 | | 1 | | | 1 | | | | 1 | | | 1 | | | | 1 | | | |  | | |
| Statistical analysis |  | |  | | |  | | | |  | | |  | | | |  | | | |  | | |
| 12. Were the statistical tests used to assess the relevant outcomes appropriate? | 0 | | 0 | | | 0 | | | | 0 | | | 0 | | | | 0 | | | |  | | |
| Results and conclusions |  | |  | | |  | | | |  | | |  | | | |  | | | |  | | |
| 13. Was the length of follow-up reported? | 0 | | 0 | | | 1 | | | | 1 | | | 1 | | | | 1 | | | |  | | |
| 14. Was the loss to follow-up reported? | 0 | | 0 | | | 0 | | | | 1 | | | 1 | | | | 0 | | | |  | | |
| 15. Does the study provide estimates of the random variability in the data analysis of relevant outcomes? | 1 | | 0 | | | 1 | | | | 0 | | | 0 | | | | 1 | | | |  | | |
| 16. Are adverse events reported? | 1 | | 1 | | | 1 | | | | 1 | | | 1 | | | | 1 | | | |  | | |
| 17. Are the conclusions of the study supported by results? | 1 | | 1 | | | 1 | | | | 1 | | | 1 | | | | 1 | | | |  | | |
| Competing interests and sources of support |  | |  | | |  | | | |  | | |  | | | |  | | | |  | | |
| 18. Are both competing interests and sources of support for the study reported? | 0 | | 0 | | | 0 | | | | 0 | | | 0 | | | | 0 | | | |  | | |
| Total score | 12 | | 11 | | | 12 | | | | 13 | | | 13 | | | | 13 | | | |  | | |
|  | Li Y et al (2011) | | Li Y et al (2011) | | | Hu ZK et al (2014) | | | | Lin XB et al (2014) | | | Zhenf Y et al (2011) | | | | Guo JX et al (2013) | | | |  | | |
| Study Objective |  | |  | | |  | | | |  | | |  | | | |  | | | |  | | |
| 1. Is the hypothesis/aim/objective of the study stated clearly in the abstract, introduction, or methods section? | 1 | | 1 | | | 1 | | | | 1 | | | 1 | | | | 1 | | | |  | | |
| Study Population |  | |  | | |  | | | |  | | |  | | | |  | | | |  | | |
| 2. Are the characteristics of the participants included in the study described? | 1 | | 1 | | | 1 | | | | 1 | | | 1 | | | | 1 | | | |  | | |
| 3. Were the cases collected in more than one centre? | 0 | | 0 | | | 0 | | | | 0 | | | 1 | | | | 0 | | | |  | | |
| 4. Are the eligibility criteria (inclusion and exclusion criteria) for entry into the study explicit and appropriate? | 1 | | 0 | | | 1 | | | | 1 | | | 0 | | | | 0 | | | |  | | |
| 5. Were participants recruited consecutively? | 1 | | 1 | | | 1 | | | | 1 | | | 1 | | | | 1 | | | |  | | |
| 6. Did participants enter the study at a similar point in the disease? | 1 | | 1 | | | 1 | | | | 1 | | | 1 | | | | 1 | | | |  | | |
| Intervention and co-intervention |  | |  | | |  | | | |  | | |  | | | |  | | | |  | | |
| 7. Was the intervention clearly described in the study? | 1 | | 1 | | | 1 | | | | 1 | | | 1 | | | | 1 | | | |  | | |
| 8. Were additional interventions (cointerventions) clearly reported in the study? | 1 | | 1 | | | 1 | | | | 1 | | | 1 | | | | 1 | | | |  | | |
| Outcome measures |  | |  | | |  | | | |  | | |  | | | |  | | | |  | | |
| 9. Are the outcome measures clearly defined in the introduction or methods section? | 1 | | 1 | | | 0 | | | | 1 | | | 1 | | | | 1 | | | |  | | |
| 10. Were relevant outcomes appropriately measured with objective and/or subjective methods? | 1 | | 1 | | | 1 | | | | 1 | | | 1 | | | | 1 | | | |  | | |
| 11. Were outcomes measured before and after intervention? | 1 | | 1 | | | 1 | | | | 1 | | | 1 | | | | 1 | | | |  | | |
| Statistical analysis |  | |  | | |  | | | |  | | |  | | | |  | | | |  | | |
| 12. Were the statistical tests used to assess the relevant outcomes appropriate? | 0 | | 0 | | | 0 | | | | 0 | | | 0 | | | | 0 | | | |  | | |
| Results and conclusions |  | |  | | |  | | | |  | | |  | | | |  | | | |  | | |
| 13. Was the length of follow-up reported? | 1 | | 1 | | | 0 | | | | 1 | | | 1 | | | | 1 | | | |  | | |
| 14. Was the loss to follow-up reported? | 1 | | 1 | | | 0 | | | | 1 | | | 0 | | | | 1 | | | |  | | |
| 15. Does the study provide estimates of the random variability in the data analysis of relevant outcomes? | 1 | | 0 | | | 1 | | | | 0 | | | 0 | | | | 0 | | | |  | | |
| 16. Are adverse events reported? | 1 | | 1 | | | 1 | | | | 1 | | | 1 | | | | 1 | | | |  | | |
| 17. Are the conclusions of the study supported by results? | 1 | | 1 | | | 1 | | | | 1 | | | 1 | | | | 1 | | | |  | | |
| Competing interests and sources of support |  | |  | | |  | | | |  | | |  | | | |  | | | |  | | |
| 18. Are both competing interests and sources of support for the study reported? | 0 | | 0 | | | 0 | | | | 0 | | | 0 | | | | 0 | | | |  | | |
| Total score | 15 | | 13 | | | 12 | | | | 14 | | | 13 | | | | 13 | | | |  | | |
|  | Zhang YF et al (2014) | | Lu GL et al (2015) | | | Guo JX (2012) | | | | Zhang YZ et al (2012) | | | Qiao JJ et al (2015) | | | | Song Y et al (2015) | | | |  | | |
| Study Objective |  | |  | | |  | | | |  | | |  | | | |  | | | |  | | |
| 1. Is the hypothesis/aim/objective of the study stated clearly in the abstract, introduction, or methods section? | 1 | | 1 | | | 1 | | | | 1 | | | 1 | | | | 1 | | | |  | | |
| Study Population |  | |  | | |  | | | |  | | |  | | | |  | | | |  | | |
| 2. Are the characteristics of the participants included in the study described? | 1 | | 1 | | | 1 | | | | 1 | | | 1 | | | | 1 | | | |  | | |
| 3. Were the cases collected in more than one centre? | 0 | | 0 | | | 0 | | | | 1 | | | 0 | | | | 0 | | | |  | | |
| 4. Are the eligibility criteria (inclusion and exclusion criteria) for entry into the study explicit and appropriate? | 0 | | 0 | | | 0 | | | | 0 | | | 0 | | | | 0 | | | |  | | |
| 5. Were participants recruited consecutively? | 1 | | 1 | | | 1 | | | | 1 | | | 1 | | | | 1 | | | |  | | |
| 6. Did participants enter the study at a similar point in the disease? | 1 | | 1 | | | 1 | | | | 1 | | | 1 | | | | 1 | | | |  | | |
| Intervention and co-intervention |  | |  | | |  | | | |  | | |  | | | |  | | | |  | | |
| 7. Was the intervention clearly described in the study? | 1 | | 1 | | | 1 | | | | 1 | | | 1 | | | | 1 | | | |  | | |
| 8. Were additional interventions (cointerventions) clearly reported in the study? | 1 | | 1 | | | 1 | | | | 1 | | | 1 | | | | 1 | | | |  | | |
| Outcome measures |  | |  | | |  | | | |  | | |  | | | |  | | | |  | | |
| 9. Are the outcome measures clearly defined in the introduction or methods section? | 1 | | 0 | | | 1 | | | | 1 | | | 1 | | | | 1 | | | |  | | |
| 10. Were relevant outcomes appropriately measured with objective and/or subjective methods? | 1 | | 1 | | | 1 | | | | 1 | | | 1 | | | | 1 | | | |  | | |
| 11. Were outcomes measured before and after intervention? | 1 | | 1 | | | 1 | | | | 1 | | | 1 | | | | 1 | | | |  | | |
| Statistical analysis |  | |  | | |  | | | |  | | |  | | | |  | | | |  | | |
| 12. Were the statistical tests used to assess the relevant outcomes appropriate? | 0 | | 0 | | | 0 | | | | 0 | | | 0 | | | | 0 | | | |  | | |
| Results and conclusions |  | |  | | |  | | | |  | | |  | | | |  | | | |  | | |
| 13. Was the length of follow-up reported? | 1 | | 1 | | | 0 | | | | 1 | | | 1 | | | | 1 | | | |  | | |
| 14. Was the loss to follow-up reported? | 1 | | 1 | | | 0 | | | | 0 | | | 0 | | | | 0 | | | |  | | |
| 15. Does the study provide estimates of the random variability in the data analysis of relevant outcomes? | 1 | | 1 | | | 0 | | | | 1 | | | 0 | | | | 1 | | | |  | | |
| 16. Are adverse events reported? | 1 | | 1 | | | 1 | | | | 1 | | | 1 | | | | 1 | | | |  | | |
| 17. Are the conclusions of the study supported by results? | 1 | | 1 | | | 1 | | | | 1 | | | 1 | | | | 1 | | | |  | | |
| Competing interests and sources of support |  | |  | | |  | | | |  | | |  | | | |  | | | |  | | |
| 18. Are both competing interests and sources of support for the study reported? | 0 | | 0 | | | 0 | | | | 0 | | | 0 | | | | 0 | | | |  | | |
| Total score | 14 | | 13 | | | 11 | | | | 14 | | | 12 | | | | 13 | | | |  | | |
|  | Zheng BR et al (2013) | | Qi JR et al (2009) | | | Xing QS et al (2011) | | | | Xing QS et al (2010) | | | Yin SL et al (2014) | | | | Zhang GC et al (2012) | | | |  | | |
| Study Objective |  | |  | | |  | | | |  | | |  | | | |  | | | |  | | |
| 1. Is the hypothesis/aim/objective of the study stated clearly in the abstract, introduction, or methods section? | 1 | | 0 | | | 1 | | | | 1 | | | 1 | | | | 0 | | | |  | | |
| Study Population |  | |  | | |  | | | |  | | |  | | | |  | | | |  | | |
| 2. Are the characteristics of the participants included in the study described? | 1 | | 1 | | | 1 | | | | 1 | | | 1 | | | | 1 | | | |  | | |
| 3. Were the cases collected in more than one centre? | 0 | | 0 | | | 1 | | | | 0 | | | 0 | | | | 0 | | | |  | | |
| 4. Are the eligibility criteria (inclusion and exclusion criteria) for entry into the study explicit and appropriate? | 0 | | 0 | | | 0 | | | | 1 | | | 1 | | | | 0 | | | |  | | |
| 5. Were participants recruited consecutively? | 1 | | 1 | | | 1 | | | | 1 | | | 1 | | | | 1 | | | |  | | |
| 6. Did participants enter the study at a similar point in the disease? | 1 | | 1 | | | 1 | | | | 1 | | | 1 | | | | 1 | | | |  | | |
| Intervention and co-intervention |  | |  | | |  | | | |  | | |  | | | |  | | | |  | | |
| 7. Was the intervention clearly described in the study? | 1 | | 1 | | | 1 | | | | 1 | | | 1 | | | | 1 | | | |  | | |
| 8. Were additional interventions (cointerventions) clearly reported in the study? | 1 | | 1 | | | 1 | | | | 1 | | | 1 | | | | 1 | | | |  | | |
| Outcome measures |  | |  | | |  | | | |  | | |  | | | |  | | | |  | | |
| 9. Are the outcome measures clearly defined in the introduction or methods section? | 1 | | 1 | | | 1 | | | | 1 | | | 1 | | | | 1 | | | |  | | |
| 10. Were relevant outcomes appropriately measured with objective and/or subjective methods? | 1 | | 1 | | | 1 | | | | 1 | | | 1 | | | | 1 | | | |  | | |
| 11. Were outcomes measured before and after intervention? | 1 | | 1 | | | 1 | | | | 1 | | | 1 | | | | 1 | | | |  | | |
| Statistical analysis |  | |  | | |  | | | |  | | |  | | | |  | | | |  | | |
| 12. Were the statistical tests used to assess the relevant outcomes appropriate? | 0 | | 0 | | | 0 | | | | 0 | | | 0 | | | | 0 | | | |  | | |
| Results and conclusions |  | |  | | |  | | | |  | | |  | | | |  | | | |  | | |
| 13. Was the length of follow-up reported? | 1 | | 1 | | | 1 | | | | 1 | | | 1 | | | | 0 | | | |  | | |
| 14. Was the loss to follow-up reported? | 0 | | 1 | | | 1 | | | | 1 | | | 1 | | | | 0 | | | |  | | |
| 15. Does the study provide estimates of the random variability in the data analysis of relevant outcomes? | 0 | | 0 | | | 1 | | | | 1 | | | 1 | | | | 1 | | | |  | | |
| 16. Are adverse events reported? | 1 | | 1 | | | 1 | | | | 1 | | | 1 | | | | 1 | | | |  | | |
| 17. Are the conclusions of the study supported by results? | 1 | | 1 | | | 1 | | | | 1 | | | 1 | | | | 1 | | | |  | | |
| Competing interests and sources of support |  | |  | | |  | | | |  | | |  | | | |  | | | |  | | |
| 18. Are both competing interests and sources of support for the study reported? | 0 | | 0 | | | 0 | | | | 0 | | | 0 | | | | 0 | | | |  | | |
| Total score | 12 | | 12 | | | 15 | | | | 15 | | | 15 | | | | 11 | | | |  | | |
|  | Li SZ et al (2014) | | Zeng XJ et al (2009) | | | Hou YB et al (2015) | | | | Zhu XL et al (2014) | | | Yang CY et al (2014) | | | | Zhang W et al (2011) | | | |  | | |
| Study Objective |  | |  | | |  | | | |  | | |  | | | |  | | | |  | | |
| 1. Is the hypothesis/aim/objective of the study stated clearly in the abstract, introduction, or methods section? | 1 | | 1 | | | 1 | | | | 1 | | | 1 | | | | 1 | | | |  | | |
| Study Population |  | |  | | |  | | | |  | | |  | | | |  | | | |  | | |
| 2. Are the characteristics of the participants included in the study described? | 1 | | 1 | | | 1 | | | | 1 | | | 1 | | | | 1 | | | |  | | |
| 3. Were the cases collected in more than one centre? | 0 | | 0 | | | 0 | | | | 0 | | | 0 | | | | 0 | | | |  | | |
| 4. Are the eligibility criteria (inclusion and exclusion criteria) for entry into the study explicit and appropriate? | 0 | | 1 | | | 0 | | | | 0 | | | 0 | | | | 0 | | | |  | | |
| 5. Were participants recruited consecutively? | 1 | | 1 | | | 1 | | | | 1 | | | 1 | | | | 1 | | | |  | | |
| 6. Did participants enter the study at a similar point in the disease? | 1 | | 1 | | | 1 | | | | 1 | | | 1 | | | | 1 | | | |  | | |
| Intervention and co-intervention |  | |  | | |  | | | |  | | |  | | | |  | | | |  | | |
| 7. Was the intervention clearly described in the study? | 1 | | 1 | | | 1 | | | | 1 | | | 1 | | | | 1 | | | |  | | |
| 8. Were additional interventions (cointerventions) clearly reported in the study? | 1 | | 1 | | | 1 | | | | 1 | | | 1 | | | | 1 | | | |  | | |
| Outcome measures |  | |  | | |  | | | |  | | |  | | | |  | | | |  | | |
| 9. Are the outcome measures clearly defined in the introduction or methods section? | 1 | | 1 | | | 0 | | | | 1 | | | 0 | | | | 0 | | | |  | | |
| 10. Were relevant outcomes appropriately measured with objective and/or subjective methods? | 1 | | 1 | | | 1 | | | | 1 | | | 1 | | | | 1 | | | |  | | |
| 11. Were outcomes measured before and after intervention? | 1 | | 1 | | | 1 | | | | 1 | | | 1 | | | | 1 | | | |  | | |
| Statistical analysis |  | |  | | |  | | | |  | | |  | | | |  | | | |  | | |
| 12. Were the statistical tests used to assess the relevant outcomes appropriate? | 0 | | 0 | | | 0 | | | | 0 | | | 0 | | | | 0 | | | |  | | |
| Results and conclusions |  | |  | | |  | | | |  | | |  | | | |  | | | |  | | |
| 13. Was the length of follow-up reported? | 1 | | 0 | | | 1 | | | | 1 | | | 0 | | | | 1 | | | |  | | |
| 14. Was the loss to follow-up reported? | 1 | | 0 | | | 0 | | | | 0 | | | 0 | | | | 0 | | | |  | | |
| 15. Does the study provide estimates of the random variability in the data analysis of relevant outcomes? | 0 | | 1 | | | 1 | | | | 1 | | | 0 | | | | 0 | | | |  | | |
| 16. Are adverse events reported? | 1 | | 1 | | | 1 | | | | 1 | | | 1 | | | | 1 | | | |  | | |
| 17. Are the conclusions of the study supported by results? | 1 | | 1 | | | 1 | | | | 1 | | | 1 | | | | 1 | | | |  | | |
| Competing interests and sources of support |  | |  | | |  | | | |  | | |  | | | |  | | | |  | | |
| 18. Are both competing interests and sources of support for the study reported? | 0 | | 0 | | | 0 | | | | 0 | | | 0 | | | | 0 | | | |  | | |
| Total score | 13 | | 13 | | | 12 | | | | 13 | | | 10 | | | | 11 | | | |  | | |
|  | Liu HY et al (2013) | | Wang SM et al (2013) | | | Lin Y et al (2010) | | | | Wang H et al (2012) | | | Xu HS et al (2013) | | | | Wan LZ et al (2010) | | | |  | | |
| Study Objective |  | |  | | |  | | | |  | | |  | | | |  | | | |  | | |
| 1. Is the hypothesis/aim/objective of the study stated clearly in the abstract, introduction, or methods section? | 1 | | 1 | | | 1 | | | | 1 | | | 1 | | | | 1 | | | |  | | |
| Study Population |  | |  | | |  | | | |  | | |  | | | |  | | | |  | | |
| 2. Are the characteristics of the participants included in the study described? | 1 | | 1 | | | 1 | | | | 1 | | | 1 | | | | 1 | | | |  | | |
| 3. Were the cases collected in more than one centre? | 0 | | 0 | | | 0 | | | | 0 | | | 0 | | | | 0 | | | |  | | |
| 4. Are the eligibility criteria (inclusion and exclusion criteria) for entry into the study explicit and appropriate? | 0 | | 0 | | | 0 | | | | 0 | | | 0 | | | | 1 | | | |  | | |
| 5. Were participants recruited consecutively? | 1 | | 1 | | | 1 | | | | 1 | | | 1 | | | | 1 | | | |  | | |
| 6. Did participants enter the study at a similar point in the disease? | 1 | | 1 | | | 1 | | | | 1 | | | 1 | | | | 1 | | | |  | | |
| Intervention and co-intervention |  | |  | | |  | | | |  | | |  | | | |  | | | |  | | |
| 7. Was the intervention clearly described in the study? | 1 | | 1 | | | 1 | | | | 1 | | | 1 | | | | 1 | | | |  | | |
| 8. Were additional interventions (cointerventions) clearly reported in the study? | 1 | | 1 | | | 1 | | | | 1 | | | 1 | | | | 1 | | | |  | | |
| Outcome measures |  | |  | | |  | | | |  | | |  | | | |  | | | |  | | |
| 9. Are the outcome measures clearly defined in the introduction or methods section? | 1 | | 1 | | | 1 | | | | 1 | | | 1 | | | | 1 | | | |  | | |
| 10. Were relevant outcomes appropriately measured with objective and/or subjective methods? | 1 | | 1 | | | 1 | | | | 1 | | | 1 | | | | 1 | | | |  | | |
| 11. Were outcomes measured before and after intervention? | 1 | | 1 | | | 1 | | | | 1 | | | 1 | | | | 1 | | | |  | | |
| Statistical analysis |  | |  | | |  | | | |  | | |  | | | |  | | | |  | | |
| 12. Were the statistical tests used to assess the relevant outcomes appropriate? | 0 | | 0 | | | 1 | | | | 0 | | | 0 | | | | 0 | | | |  | | |
| Results and conclusions |  | |  | | |  | | | |  | | |  | | | |  | | | |  | | |
| 13. Was the length of follow-up reported? | 1 | | 1 | | | 1 | | | | 0 | | | 0 | | | | 1 | | | |  | | |
| 14. Was the loss to follow-up reported? | 1 | | 0 | | | 0 | | | | 0 | | | 0 | | | | 1 | | | |  | | |
| 15. Does the study provide estimates of the random variability in the data analysis of relevant outcomes? | 1 | | 0 | | | 1 | | | | 0 | | | 0 | | | | 0 | | | |  | | |
| 16. Are adverse events reported? | 1 | | 1 | | | 1 | | | | 1 | | | 1 | | | | 1 | | | |  | | |
| 17. Are the conclusions of the study supported by results? | 1 | | 1 | | | 1 | | | | 1 | | | 1 | | | | 1 | | | |  | | |
| Competing interests and sources of support |  | |  | | |  | | | |  | | |  | | | |  | | | |  | | |
| 18. Are both competing interests and sources of support for the study reported? | 0 | | 0 | | | 0 | | | | 0 | | | 0 | | | | 0 | | | |  | | |
| Total score | 14 | | 12 | | | 14 | | | | 11 | | | 11 | | | | 14 | | | |  | | |
|  | Xi DW et al (2015) | | Yu CZ et al (2012) | | | Li JH et al (2012) | | | | Liu HY et al (2012) | | | Li F et al (2007) | | | | Xing QS et al (2011) | | | |  | | |
| Study Objective |  | |  | | |  | | | |  | | |  | | | |  | | | |  | | |
| 1. Is the hypothesis/aim/objective of the study stated clearly in the abstract, introduction, or methods section? | 1 | | 1 | | | 1 | | | | 1 | | | 1 | | | | 1 | | | |  | | |
| Study Population |  | |  | | |  | | | |  | | |  | | | |  | | | |  | | |
| 2. Are the characteristics of the participants included in the study described? | 1 | | 1 | | | 1 | | | | 0 | | | 1 | | | | 1 | | | |  | | |
| 3. Were the cases collected in more than one centre? | 0 | | 0 | | | 0 | | | | 0 | | | 0 | | | | 0 | | | |  | | |
| 4. Are the eligibility criteria (inclusion and exclusion criteria) for entry into the study explicit and appropriate? | 0 | | 0 | | | 0 | | | | 0 | | | 0 | | | | 0 | | | |  | | |
| 5. Were participants recruited consecutively? | 1 | | 1 | | | 1 | | | | 1 | | | 1 | | | | 1 | | | |  | | |
| 6. Did participants enter the study at a similar point in the disease? | 1 | | 1 | | | 1 | | | | 1 | | | 1 | | | | 1 | | | |  | | |
| Intervention and co-intervention |  | |  | | |  | | | |  | | |  | | | |  | | | |  | | |
| 7. Was the intervention clearly described in the study? | 1 | | 0 | | | 1 | | | | 0 | | | 1 | | | | 1 | | | |  | | |
| 8. Were additional interventions (cointerventions) clearly reported in the study? | 1 | | 0 | | | 1 | | | | 0 | | | 1 | | | | 1 | | | |  | | |
| Outcome measures |  | |  | | |  | | | |  | | |  | | | |  | | | |  | | |
| 9. Are the outcome measures clearly defined in the introduction or methods section? | 0 | | 0 | | | 1 | | | | 0 | | | 0 | | | | 1 | | | |  | | |
| 10. Were relevant outcomes appropriately measured with objective and/or subjective methods? | 1 | | 1 | | | 1 | | | | 1 | | | 1 | | | | 1 | | | |  | | |
| 11. Were outcomes measured before and after intervention? | 1 | | 1 | | | 1 | | | | 1 | | | 1 | | | | 1 | | | |  | | |
| Statistical analysis |  | |  | | |  | | | |  | | |  | | | |  | | | |  | | |
| 12. Were the statistical tests used to assess the relevant outcomes appropriate? | 0 | | 0 | | | 0 | | | | 0 | | | 0 | | | | 0 | | | |  | | |
| Results and conclusions |  | |  | | |  | | | |  | | |  | | | |  | | | |  | | |
| 13. Was the length of follow-up reported? | 1 | | 1 | | | 1 | | | | 1 | | | 0 | | | | 1 | | | |  | | |
| 14. Was the loss to follow-up reported? | 1 | | 0 | | | 0 | | | | 0 | | | 0 | | | | 0 | | | |  | | |
| 15. Does the study provide estimates of the random variability in the data analysis of relevant outcomes? | 1 | | 0 | | | 1 | | | | 0 | | | 0 | | | | 0 | | | |  | | |
| 16. Are adverse events reported? | 1 | | 1 | | | 1 | | | | 1 | | | 1 | | | | 1 | | | |  | | |
| 17. Are the conclusions of the study supported by results? | 1 | | 1 | | | 1 | | | | 1 | | | 1 | | | | 1 | | | |  | | |
| Competing interests and sources of support |  | |  | | |  | | | |  | | |  | | | |  | | | |  | | |
| 18. Are both competing interests and sources of support for the study reported? | 0 | | 0 | | | 0 | | | | 0 | | | 0 | | | | 0 | | | |  | | |
| Total score | 13 | | 9 | | | 13 | | | | 8 | | | 10 | | | | 12 | | | |  | | |
|  | Xing QS et al (2007) | | Hu JW (2015) | | | Guo WB (2010) | | | | Mao ZG et al (2016) | | | Ma LC et al (2016) | | | | Zhu D et al (2015) | | | |  | | |
| Study Objective |  | |  | | |  | | | |  | | |  | | | |  | | | |  | | |
| 1. Is the hypothesis/aim/objective of the study stated clearly in the abstract, introduction, or methods section? | 1 | | 1 | | | 1 | | | | 1 | | | 1 | | | | 1 | | | |  | | |
| Study Population |  | |  | | |  | | | |  | | |  | | | |  | | | |  | | |
| 2. Are the characteristics of the participants included in the study described? | 1 | | 1 | | | 0 | | | | 1 | | | 1 | | | | 1 | | | |  | | |
| 3. Were the cases collected in more than one centre? | 0 | | 0 | | | 0 | | | | 0 | | | 0 | | | | 0 | | | |  | | |
| 4. Are the eligibility criteria (inclusion and exclusion criteria) for entry into the study explicit and appropriate? | 0 | | 1 | | | 0 | | | | 0 | | | 0 | | | | 0 | | | |  | | |
| 5. Were participants recruited consecutively? | 1 | | 1 | | | 0 | | | | 1 | | | 1 | | | | 1 | | | |  | | |
| 6. Did participants enter the study at a similar point in the disease? | 1 | | 1 | | | 1 | | | | 1 | | | 1 | | | | 1 | | | |  | | |
| Intervention and co-intervention |  | |  | | |  | | | |  | | |  | | | |  | | | |  | | |
| 7. Was the intervention clearly described in the study? | 1 | | 1 | | | 1 | | | | 1 | | | 1 | | | | 1 | | | |  | | |
| 8. Were additional interventions (cointerventions) clearly reported in the study? | 1 | | 1 | | | 1 | | | | 1 | | | 1 | | | | 1 | | | |  | | |
| Outcome measures |  | |  | | |  | | | |  | | |  | | | |  | | | |  | | |
| 9. Are the outcome measures clearly defined in the introduction or methods section? | 1 | | 1 | | | 0 | | | | 1 | | | 1 | | | | 1 | | | |  | | |
| 10. Were relevant outcomes appropriately measured with objective and/or subjective methods? | 1 | | 1 | | | 1 | | | | 1 | | | 1 | | | | 1 | | | |  | | |
| 11. Were outcomes measured before and after intervention? | 1 | | 1 | | | 1 | | | | 1 | | | 1 | | | | 1 | | | |  | | |
| Statistical analysis |  | |  | | |  | | | |  | | |  | | | |  | | | |  | | |
| 12. Were the statistical tests used to assess the relevant outcomes appropriate? | 0 | | 1 | | | 0 | | | | 0 | | | 0 | | | | 0 | | | |  | | |
| Results and conclusions |  | |  | | |  | | | |  | | |  | | | |  | | | |  | | |
| 13. Was the length of follow-up reported? | 0 | | 1 | | | 1 | | | | 1 | | | 1 | | | | 1 | | | |  | | |
| 14. Was the loss to follow-up reported? | 0 | | 1 | | | 0 | | | | 0 | | | 1 | | | | 1 | | | |  | | |
| 15. Does the study provide estimates of the random variability in the data analysis of relevant outcomes? | 0 | | 1 | | | 1 | | | | 1 | | | 1 | | | | 1 | | | |  | | |
| 16. Are adverse events reported? | 1 | | 1 | | | 1 | | | | 1 | | | 1 | | | | 1 | | | |  | | |
| 17. Are the conclusions of the study supported by results? | 1 | | 1 | | | 1 | | | | 1 | | | 1 | | | | 1 | | | |  | | |
| Competing interests and sources of support |  | |  | | |  | | | |  | | |  | | | |  | | | |  | | |
| 18. Are both competing interests and sources of support for the study reported? | 0 | | 0 | | | 0 | | | | 0 | | | 0 | | | | 0 | | | |  | | |
| Total score | 11 | | 16 | | | 10 | | | | 13 | | | 14 | | | | 14 | | | |  | | |
|  | Yang SH et al (2015) | Shen S et al (2013) | | | Cao Y et al (2015) | | | | MUHOOZI RWAKARYEBE MBAGGA MD (2014) | | | | | | | Yu CJ et al (2013) | | | | Wu Q et al (2013) | | | |
| Study Objective |  |  | | |  | | | |  | | | | | | |  | | | |  | | | |
| 1. Is the hypothesis/aim/objective of the study stated clearly in the abstract, introduction, or methods section? | 1 | 1 | | | 1 | | | | 1 | | | | | | | 1 | | | | 1 | | | |
| Study Population |  |  | | |  | | | |  | | | | | | |  | | | |  | | | |
| 2. Are the characteristics of the participants included in the study described? | 1 | 1 | | | 1 | | | | 1 | | | | | | | 1 | | | | 1 | | | |
| 3. Were the cases collected in more than one centre? | 0 | 0 | | | 0 | | | | 0 | | | | | | | 0 | | | | 1 | | | |
| 4. Are the eligibility criteria (inclusion and exclusion criteria) for entry into the study explicit and appropriate? | 0 | 0 | | | 0 | | | | 0 | | | | | | | 0 | | | | 1 | | | |
| 5. Were participants recruited consecutively? | 1 | 1 | | | 1 | | | | 1 | | | | | | | 1 | | | | 1 | | | |
| 6. Did participants enter the study at a similar point in the disease? | 1 | 1 | | | 1 | | | | 1 | | | | | | | 1 | | | | 1 | | | |
| Intervention and co-intervention |  |  | | |  | | | |  | | | | | | |  | | | |  | | | |
| 7. Was the intervention clearly described in the study? | 1 | 1 | | | 1 | | | | 1 | | | | | | | 1 | | | | 1 | | | |
| 8. Were additional interventions (cointerventions) clearly reported in the study? | 1 | 1 | | | 1 | | | | 1 | | | | | | | 1 | | | | 1 | | | |
| Outcome measures |  |  | | |  | | | |  | | | | | | |  | | | |  | | | |
| 9. Are the outcome measures clearly defined in the introduction or methods section? | 0 | 1 | | | 1 | | | | 0 | | | | | | | 0 | | | | 1 | | | |
| 10. Were relevant outcomes appropriately measured with objective and/or subjective methods? | 1 | 1 | | | 1 | | | | 1 | | | | | | | 1 | | | | 1 | | | |
| 11. Were outcomes measured before and after intervention? | 1 | 1 | | | 1 | | | | 1 | | | | | | | 1 | | | | 1 | | | |
| Statistical analysis |  |  | | |  | | | |  | | | | | | |  | | | |  | | | |
| 12. Were the statistical tests used to assess the relevant outcomes appropriate? | 0 | 0 | | | 0 | | | | 0 | | | | | | | 0 | | | | 1 | | | |
| Results and conclusions |  |  | | |  | | | |  | | | | | | |  | | | |  | | | |
| 13. Was the length of follow-up reported? | 1 | 1 | | | 0 | | | | 0 | | | | | | | 1 | | | | 1 | | | |
| 14. Was the loss to follow-up reported? | 1 | 1 | | | 0 | | | | 0 | | | | | | | 0 | | | | 1 | | | |
| 15. Does the study provide estimates of the random variability in the data analysis of relevant outcomes? | 0 | 1 | | | 1 | | | | 1 | | | | | | | 1 | | | | 1 | | | |
| 16. Are adverse events reported? | 1 | 1 | | | 1 | | | | 1 | | | | | | | 1 | | | | 1 | | | |
| 17. Are the conclusions of the study supported by results? | 1 | 1 | | | 1 | | | | 1 | | | | | | | 1 | | | | 1 | | | |
| Competing interests and sources of support |  |  | | |  | | | |  | | | | | | |  | | | |  | | | |
| 18. Are both competing interests and sources of support for the study reported? | 0 | 0 | | | 0 | | | | 0 | | | | | | | 0 | | | | 0 | | | |
| Total score | 12 | 14 | | | 12 | | | | 11 | | | | | | | 12 | | | | 17 | | | |
|  | Lin ZB et al (2013) | | Wan L et al (2013) | | | Liu J et al (2014) | | | | Xing QS et al (2015) | | | Ma LC et al (2016) | | | | Dong HJ et al (2015) | | | |  | | |
| Study Objective |  | |  | | |  | | | |  | | |  | | | |  | | | |  | | |
| 1. Is the hypothesis/aim/objective of the study stated clearly in the abstract, introduction, or methods section? | 1 | | 1 | | | 1 | | | | 1 | | | 1 | | | | 1 | | | |  | | |
| Study Population |  | |  | | |  | | | |  | | |  | | | |  | | | |  | | |
| 2. Are the characteristics of the participants included in the study described? | 1 | | 1 | | | 1 | | | | 1 | | | 1 | | | | 1 | | | |  | | |
| 3. Were the cases collected in more than one centre? | 0 | | 0 | | | 0 | | | | 0 | | | 0 | | | | 0 | | | |  | | |
| 4. Are the eligibility criteria (inclusion and exclusion criteria) for entry into the study explicit and appropriate? | 0 | | 0 | | | 0 | | | | 0 | | | 0 | | | | 1 | | | |  | | |
| 5. Were participants recruited consecutively? | 1 | | 1 | | | 1 | | | | 1 | | | 1 | | | | 1 | | | |  | | |
| 6. Did participants enter the study at a similar point in the disease? | 1 | | 1 | | | 1 | | | | 1 | | | 1 | | | | 1 | | | |  | | |
| Intervention and co-intervention |  | |  | | |  | | | |  | | |  | | | |  | | | |  | | |
| 7. Was the intervention clearly described in the study? | 1 | | 1 | | | 1 | | | | 0 | | | 1 | | | | 1 | | | |  | | |
| 8. Were additional interventions (cointerventions) clearly reported in the study? | 1 | | 1 | | | 1 | | | | 0 | | | 1 | | | | 1 | | | |  | | |
| Outcome measures |  | |  | | |  | | | |  | | |  | | | |  | | | |  | | |
| 9. Are the outcome measures clearly defined in the introduction or methods section? | 1 | | 1 | | | 1 | | | | 1 | | | 1 | | | | 1 | | | |  | | |
| 10. Were relevant outcomes appropriately measured with objective and/or subjective methods? | 1 | | 1 | | | 1 | | | | 1 | | | 1 | | | | 1 | | | |  | | |
| 11. Were outcomes measured before and after intervention? | 1 | | 1 | | | 1 | | | | 1 | | | 1 | | | | 1 | | | |  | | |
| Statistical analysis |  | |  | | |  | | | |  | | |  | | | |  | | | |  | | |
| 12. Were the statistical tests used to assess the relevant outcomes appropriate? | 0 | | 0 | | | 0 | | | | 0 | | | 0 | | | | 0 | | | |  | | |
| Results and conclusions |  | |  | | |  | | | |  | | |  | | | |  | | | |  | | |
| 13. Was the length of follow-up reported? | 1 | | 1 | | | 1 | | | | 0 | | | 1 | | | | 1 | | | |  | | |
| 14. Was the loss to follow-up reported? | 1 | | 1 | | | 1 | | | | 1 | | | 1 | | | | 1 | | | |  | | |
| 15. Does the study provide estimates of the random variability in the data analysis of relevant outcomes? | 1 | | 0 | | | 1 | | | | 0 | | | 1 | | | | 1 | | | |  | | |
| 16. Are adverse events reported? | 1 | | 1 | | | 1 | | | | 1 | | | 1 | | | | 1 | | | |  | | |
| 17. Are the conclusions of the study supported by results? | 1 | | 1 | | | 1 | | | | 1 | | | 1 | | | | 1 | | | |  | | |
| Competing interests and sources of support |  | |  | | |  | | | |  | | |  | | | |  | | | |  | | |
| 18. Are both competing interests and sources of support for the study reported? | 0 | | 0 | | | 0 | | | | 0 | | | 0 | | | | 0 | | | |  | | |
| Total score | 14 | | 13 | | | 14 | | | | 10 | | | 14 | | | | 15 | | | |  | | |
|  | Xing QS et al (2007) | | Zhu XM et al (2013) | | | Wang XN et al (2012) | | | | Liu Y et al (2016) | | | Song SB et al (2016) | | | | Lin K et al (2008) | | | |  | | |
| Study Objective |  | |  | | |  | | | |  | | |  | | | |  | | | |  | | |
| 1. Is the hypothesis/aim/objective of the study stated clearly in the abstract, introduction, or methods section? | 1 | | 1 | | | 1 | | | | 1 | | | 1 | | | | 1 | | | |  | | |
| Study Population |  | |  | | |  | | | |  | | |  | | | |  | | | |  | | |
| 2. Are the characteristics of the participants included in the study described? | 1 | | 1 | | | 1 | | | | 1 | | | 1 | | | | 1 | | | |  | | |
| 3. Were the cases collected in more than one centre? | 0 | | 0 | | | 0 | | | | 0 | | | 0 | | | | 0 | | | |  | | |
| 4. Are the eligibility criteria (inclusion and exclusion criteria) for entry into the study explicit and appropriate? | 0 | | 0 | | | 0 | | | | 1 | | | 1 | | | | 0 | | | |  | | |
| 5. Were participants recruited consecutively? | 1 | | 1 | | | 1 | | | | 1 | | | 1 | | | | 1 | | | |  | | |
| 6. Did participants enter the study at a similar point in the disease? | 1 | | 1 | | | 1 | | | | 1 | | | 1 | | | | 1 | | | |  | | |
| Intervention and co-intervention |  | |  | | |  | | | |  | | |  | | | |  | | | |  | | |
| 7. Was the intervention clearly described in the study? | 1 | | 1 | | | 1 | | | | 1 | | | 1 | | | | 1 | | | |  | | |
| 8. Were additional interventions (cointerventions) clearly reported in the study? | 1 | | 1 | | | 1 | | | | 1 | | | 1 | | | | 1 | | | |  | | |
| Outcome measures |  | |  | | |  | | | |  | | |  | | | |  | | | |  | | |
| 9. Are the outcome measures clearly defined in the introduction or methods section? | 1 | | 1 | | | 1 | | | | 1 | | | 1 | | | | 1 | | | |  | | |
| 10. Were relevant outcomes appropriately measured with objective and/or subjective methods? | 1 | | 1 | | | 1 | | | | 1 | | | 1 | | | | 1 | | | |  | | |
| 11. Were outcomes measured before and after intervention? | 1 | | 1 | | | 1 | | | | 1 | | | 1 | | | | 1 | | | |  | | |
| Statistical analysis |  | |  | | |  | | | |  | | |  | | | |  | | | |  | | |
| 12. Were the statistical tests used to assess the relevant outcomes appropriate? | 0 | | 0 | | | 0 | | | | 1 | | | 0 | | | | 0 | | | |  | | |
| Results and conclusions |  | |  | | |  | | | |  | | |  | | | |  | | | |  | | |
| 13. Was the length of follow-up reported? | 1 | | 1 | | | 1 | | | | 1 | | | 1 | | | | 1 | | | |  | | |
| 14. Was the loss to follow-up reported? | 1 | | 1 | | | 1 | | | | 1 | | | 0 | | | | 0 | | | |  | | |
| 15. Does the study provide estimates of the random variability in the data analysis of relevant outcomes? | 0 | | 0 | | | 0 | | | | 1 | | | 1 | | | | 1 | | | |  | | |
| 16. Are adverse events reported? | 1 | | 1 | | | 1 | | | | 1 | | | 1 | | | | 1 | | | |  | | |
| 17. Are the conclusions of the study supported by results? | 1 | | 1 | | | 1 | | | | 1 | | | 1 | | | | 1 | | | |  | | |
| Competing interests and sources of support |  | |  | | |  | | | |  | | |  | | | |  | | | |  | | |
| 18. Are both competing interests and sources of support for the study reported? | 0 | | 0 | | | 0 | | | | 0 | | | 0 | | | | 0 | | | |  | | |
| Total score | 13 | | 13 | | | 13 | | | | 16 | | | 14 | | | | 13 | | | |  | | |
|  | Wang SX et al (2014) | | Wang C (2013) | | | Wang KX et al (2014) | | | | Liang F (2014) | | | Bian H et al (2012) | | | | Xing QS (2013) | | | |  | | |
| Study Objective |  | |  | | |  | | | |  | | |  | | | |  | | | |  | | |
| 1. Is the hypothesis/aim/objective of the study stated clearly in the abstract, introduction, or methods section? | 1 | | 1 | | | 1 | | | | 1 | | | 1 | | | | 1 | | | |  | | |
| Study Population |  | |  | | |  | | | |  | | |  | | | |  | | | |  | | |
| 2. Are the characteristics of the participants included in the study described? | 1 | | 1 | | | 1 | | | | 1 | | | 1 | | | | 1 | | | |  | | |
| 3. Were the cases collected in more than one centre? | 0 | | 0 | | | 0 | | | | 0 | | | 0 | | | | 0 | | | |  | | |
| 4. Are the eligibility criteria (inclusion and exclusion criteria) for entry into the study explicit and appropriate? | 0 | | 1 | | | 0 | | | | 0 | | | 0 | | | | 0 | | | |  | | |
| 5. Were participants recruited consecutively? | 1 | | 1 | | | 1 | | | | 1 | | | 1 | | | | 1 | | | |  | | |
| 6. Did participants enter the study at a similar point in the disease? | 1 | | 1 | | | 1 | | | | 1 | | | 1 | | | | 1 | | | |  | | |
| Intervention and co-intervention |  | |  | | |  | | | |  | | |  | | | |  | | | |  | | |
| 7. Was the intervention clearly described in the study? | 1 | | 1 | | | 1 | | | | 0 | | | 1 | | | | 1 | | | |  | | |
| 8. Were additional interventions (cointerventions) clearly reported in the study? | 1 | | 1 | | | 1 | | | | 0 | | | 1 | | | | 1 | | | |  | | |
| Outcome measures |  | |  | | |  | | | |  | | |  | | | |  | | | |  | | |
| 9. Are the outcome measures clearly defined in the introduction or methods section? | 1 | | 1 | | | 0 | | | | 0 | | | 1 | | | | 1 | | | |  | | |
| 10. Were relevant outcomes appropriately measured with objective and/or subjective methods? | 1 | | 1 | | | 1 | | | | 1 | | | 1 | | | | 1 | | | |  | | |
| 11. Were outcomes measured before and after intervention? | 1 | | 1 | | | 1 | | | | 1 | | | 1 | | | | 1 | | | |  | | |
| Statistical analysis |  | |  | | |  | | | |  | | |  | | | |  | | | |  | | |
| 12. Were the statistical tests used to assess the relevant outcomes appropriate? | 0 | | 1 | | | 0 | | | | 0 | | | 1 | | | | 0 | | | |  | | |
| Results and conclusions |  | |  | | |  | | | |  | | |  | | | |  | | | |  | | |
| 13. Was the length of follow-up reported? | 1 | | 1 | | | 1 | | | | 0 | | | 1 | | | | 1 | | | |  | | |
| 14. Was the loss to follow-up reported? | 1 | | 1 | | | 1 | | | | 0 | | | 1 | | | | 1 | | | |  | | |
| 15. Does the study provide estimates of the random variability in the data analysis of relevant outcomes? | 0 | | 1 | | | 1 | | | | 0 | | | 1 | | | | 1 | | | |  | | |
| 16. Are adverse events reported? | 1 | | 1 | | | 1 | | | | 1 | | | 1 | | | | 1 | | | |  | | |
| 17. Are the conclusions of the study supported by results? | 1 | | 1 | | | 1 | | | | 1 | | | 1 | | | | 1 | | | |  | | |
| Competing interests and sources of support |  | |  | | |  | | | |  | | |  | | | |  | | | |  | | |
| 18. Are both competing interests and sources of support for the study reported? | 0 | | 0 | | | 0 | | | | 0 | | | 0 | | | | 0 | | | |  | | |
| Total score | 13 | | 16 | | | 13 | | | | 8 | | | 15 | | | | 14 | | | |  | | |
|  | Xing QS et al (2008) | | Peng Q et al (2014) | | | Zhang YZ et al (2013) | | | | Wang H et al (2016) | | | Wu Q et al (2012) | | | | Wang SM et al (2012) | | | |  | | |
| Study Objective |  | |  | | |  | | | |  | | |  | | | |  | | | |  | | |
| 1. Is the hypothesis/aim/objective of the study stated clearly in the abstract, introduction, or methods section? | 1 | | 1 | | | 1 | | | | 1 | | | 1 | | | | 1 | | | |  | | |
| Study Population |  | |  | | |  | | | |  | | |  | | | |  | | | |  | | |
| 2. Are the characteristics of the participants included in the study described? | 1 | | 1 | | | 1 | | | | 0 | | | 1 | | | | 1 | | | |  | | |
| 3. Were the cases collected in more than one centre? | 0 | | 0 | | | 0 | | | | 0 | | | 1 | | | | 0 | | | |  | | |
| 4. Are the eligibility criteria (inclusion and exclusion criteria) for entry into the study explicit and appropriate? | 0 | | 1 | | | 1 | | | | 1 | | | 0 | | | | 0 | | | |  | | |
| 5. Were participants recruited consecutively? | 1 | | 1 | | | 1 | | | | 1 | | | 1 | | | | 1 | | | |  | | |
| 6. Did participants enter the study at a similar point in the disease? | 1 | | 1 | | | 1 | | | | 1 | | | 1 | | | | 1 | | | |  | | |
| Intervention and co-intervention |  | |  | | |  | | | |  | | |  | | | |  | | | |  | | |
| 7. Was the intervention clearly described in the study? | 1 | | 1 | | | 1 | | | | 1 | | | 1 | | | | 1 | | | |  | | |
| 8. Were additional interventions (cointerventions) clearly reported in the study? | 1 | | 1 | | | 1 | | | | 1 | | | 1 | | | | 1 | | | |  | | |
| Outcome measures |  | |  | | |  | | | |  | | |  | | | |  | | | |  | | |
| 9. Are the outcome measures clearly defined in the introduction or methods section? | 1 | | 1 | | | 1 | | | | 1 | | | 1 | | | | 0 | | | |  | | |
| 10. Were relevant outcomes appropriately measured with objective and/or subjective methods? | 1 | | 1 | | | 1 | | | | 1 | | | 1 | | | | 1 | | | |  | | |
| 11. Were outcomes measured before and after intervention? | 1 | | 1 | | | 1 | | | | 1 | | | 1 | | | | 1 | | | |  | | |
| Statistical analysis |  | |  | | |  | | | |  | | |  | | | |  | | | |  | | |
| 12. Were the statistical tests used to assess the relevant outcomes appropriate? | 0 | | 0 | | | 1 | | | | 0 | | | 1 | | | | 0 | | | |  | | |
| Results and conclusions |  | |  | | |  | | | |  | | |  | | | |  | | | |  | | |
| 13. Was the length of follow-up reported? | 1 | | 1 | | | 1 | | | | 1 | | | 1 | | | | 1 | | | |  | | |
| 14. Was the loss to follow-up reported? | 1 | | 1 | | | 1 | | | | 0 | | | 1 | | | | 0 | | | |  | | |
| 15. Does the study provide estimates of the random variability in the data analysis of relevant outcomes? | 0 | | 1 | | | 1 | | | | 0 | | | 1 | | | | 0 | | | |  | | |
| 16. Are adverse events reported? | 1 | | 1 | | | 1 | | | | 1 | | | 1 | | | | 1 | | | |  | | |
| 17. Are the conclusions of the study supported by results? | 1 | | 1 | | | 1 | | | | 1 | | | 1 | | | | 1 | | | |  | | |
| Competing interests and sources of support |  | |  | | |  | | | |  | | |  | | | |  | | | |  | | |
| 18. Are both competing interests and sources of support for the study reported? | 0 | | 0 | | | 0 | | | | 0 | | | 0 | | | | 0 | | | |  | | |
| Total score | 13 | | 15 | | | 16 | | | | 12 | | | 16 | | | | 11 | | | |  | | |
|  | Lin K et al (2013) | | Haponiuk I et al (2013) | | | Liu L et al (2013) | | | | Bendaly EA et al (2011) | | | Pedra CAC et al (2009) | | | | | Fouilloux V et al (2012) | | | |  | |
| Study Objective |  | |  | | |  | | | |  | | |  | | | | |  | | | |  | |
| 1. Is the hypothesis/aim/objective of the study stated clearly in the abstract, introduction, or methods section? | 1 | | 1 | | | 1 | | | | 1 | | | 1 | | | | | 1 | | | |  | |
| Study Population |  | |  | | |  | | | |  | | |  | | | | |  | | | |  | |
| 2. Are the characteristics of the participants included in the study described? | 1 | | 1 | | | 1 | | | | 1 | | | 1 | | | | | 1 | | | |  | |
| 3. Were the cases collected in more than one centre? | 0 | | 0 | | | 0 | | | | 0 | | | 1 | | | | | 0 | | | |  | |
| 4. Are the eligibility criteria (inclusion and exclusion criteria) for entry into the study explicit and appropriate? | 1 | | 0 | | | 1 | | | | 0 | | | 1 | | | | | 1 | | | |  | |
| 5. Were participants recruited consecutively? | 1 | | 0 | | | 1 | | | | 1 | | | 1 | | | | | 1 | | | |  | |
| 6. Did participants enter the study at a similar point in the disease? | 1 | | 1 | | | 1 | | | | 1 | | | 1 | | | | | 1 | | | |  | |
| Intervention and co-intervention |  | |  | | |  | | | |  | | |  | | | | |  | | | |  | |
| 7. Was the intervention clearly described in the study? | 1 | | 1 | | | 1 | | | | 1 | | | 1 | | | | | 1 | | | |  | |
| 8. Were additional interventions (cointerventions) clearly reported in the study? | 1 | | 1 | | | 1 | | | | 1 | | | 1 | | | | | 1 | | | |  | |
| Outcome measures |  | |  | | |  | | | |  | | |  | | | | |  | | | |  | |
| 9. Are the outcome measures clearly defined in the introduction or methods section? | 1 | | 1 | | | 0 | | | | 1 | | | 1 | | | | | 1 | | | |  | |
| 10. Were relevant outcomes appropriately measured with objective and/or subjective methods? | 1 | | 1 | | | 1 | | | | 1 | | | 1 | | | | | 1 | | | |  | |
| 11. Were outcomes measured before and after intervention? | 1 | | 1 | | | 1 | | | | 1 | | | 1 | | | | | 1 | | | |  | |
| Statistical analysis |  | |  | | |  | | | |  | | |  | | | | |  | | | |  | |
| 12. Were the statistical tests used to assess the relevant outcomes appropriate? | 1 | | 0 | | | 1 | | | | 0 | | | 0 | | | | | 0 | | | |  | |
| Results and conclusions |  | |  | | |  | | | |  | | |  | | | | |  | | | |  | |
| 13. Was the length of follow-up reported? | 1 | | 1 | | | 1 | | | | 1 | | | 1 | | | | | 1 | | | |  | |
| 14. Was the loss to follow-up reported? | 1 | | 1 | | | 1 | | | | 1 | | | 1 | | | | | 1 | | | |  | |
| 15. Does the study provide estimates of the random variability in the data analysis of relevant outcomes? | 1 | | 0 | | | 1 | | | | 0 | | | 0 | | | | | 0 | | | |  | |
| 16. Are adverse events reported? | 1 | | 1 | | | 1 | | | | 1 | | | 1 | | | | | 1 | | | |  | |
| 17. Are the conclusions of the study supported by results? | 1 | | 1 | | | 1 | | | | 1 | | | 1 | | | | | 1 | | | |  | |
| Competing interests and sources of support |  | |  | | |  | | | |  | | |  | | | | |  | | | |  | |
| 18. Are both competing interests and sources of support for the study reported? | 0 | | 0 | | | 0 | | | | 0 | | | 0 | | | | | 0 | | | |  | |
| Total score | 16 | | 12 | | | 15 | | | | 13 | | | 15 | | | | | 14 | | | |  | |
|  | Thakkar B et al (2012) | | Gan C et al (2009) | | | Tao K et al (2010) | | | | Kim SJ et al (2013) | | | Lim DS et al (2007) | | | | Gao Z et al (2014) | | | |  | | |
| Study Objective |  | |  | | |  | | | |  | | |  | | | |  | | | |  | | |
| 1. Is the hypothesis/aim/objective of the study stated clearly in the abstract, introduction, or methods section? | 1 | | 1 | | | 1 | | | | 1 | | | 1 | | | | 1 | | | |  | | |
| Study Population |  | |  | | |  | | | |  | | |  | | | |  | | | |  | | |
| 2. Are the characteristics of the participants included in the study described? | 1 | | 1 | | | 1 | | | | 1 | | | 1 | | | | 1 | | | |  | | |
| 3. Were the cases collected in more than one centre? | 0 | | 0 | | | 0 | | | | 0 | | | 0 | | | | 0 | | | |  | | |
| 4. Are the eligibility criteria (inclusion and exclusion criteria) for entry into the study explicit and appropriate? | 1 | | 0 | | | 1 | | | | 0 | | | 1 | | | | 0 | | | |  | | |
| 5. Were participants recruited consecutively? | 1 | | 1 | | | 1 | | | | 1 | | | 1 | | | | 1 | | | |  | | |
| 6. Did participants enter the study at a similar point in the disease? | 1 | | 1 | | | 1 | | | | 1 | | | 1 | | | | 1 | | | |  | | |
| Intervention and co-intervention |  | |  | | |  | | | |  | | |  | | | |  | | | |  | | |
| 7. Was the intervention clearly described in the study? | 1 | | 1 | | | 1 | | | | 1 | | | 1 | | | | 1 | | | |  | | |
| 8. Were additional interventions (cointerventions) clearly reported in the study? | 1 | | 1 | | | 1 | | | | 1 | | | 1 | | | | 1 | | | |  | | |
| Outcome measures |  | |  | | |  | | | |  | | |  | | | |  | | | |  | | |
| 9. Are the outcome measures clearly defined in the introduction or methods section? | 1 | | 1 | | | 1 | | | | 1 | | | 1 | | | | 1 | | | |  | | |
| 10. Were relevant outcomes appropriately measured with objective and/or subjective methods? | 1 | | 1 | | | 1 | | | | 1 | | | 1 | | | | 1 | | | |  | | |
| 11. Were outcomes measured before and after intervention? | 1 | | 1 | | | 1 | | | | 1 | | | 1 | | | | 1 | | | |  | | |
| Statistical analysis |  | |  | | |  | | | |  | | |  | | | |  | | | |  | | |
| 12. Were the statistical tests used to assess the relevant outcomes appropriate? | 0 | | 0 | | | 1 | | | | 0 | | | 0 | | | | 0 | | | |  | | |
| Results and conclusions |  | |  | | |  | | | |  | | |  | | | |  | | | |  | | |
| 13. Was the length of follow-up reported? | 1 | | 1 | | | 1 | | | | 1 | | | 0 | | | | 1 | | | |  | | |
| 14. Was the loss to follow-up reported? | 1 | | 0 | | | 1 | | | | 1 | | | 0 | | | | 1 | | | |  | | |
| 15. Does the study provide estimates of the random variability in the data analysis of relevant outcomes? | 1 | | 1 | | | 1 | | | | 0 | | | 0 | | | | 1 | | | |  | | |
| 16. Are adverse events reported? | 1 | | 1 | | | 1 | | | | 1 | | | 1 | | | | 1 | | | |  | | |
| 17. Are the conclusions of the study supported by results? | 1 | | 1 | | | 1 | | | | 1 | | | 1 | | | | 1 | | | |  | | |
| Competing interests and sources of support |  | |  | | |  | | | |  | | |  | | | |  | | | |  | | |
| 18. Are both competing interests and sources of support for the study reported? | 0 | | 0 | | | 0 | | | | 0 | | | 0 | | | | 0 | | | |  | | |
| Total score | 15 | | 13 | | | 16 | | | | 13 | | | 12 | | | | 14 | | | |  | | |
|  | Zhu D et al (2013) | | Chen Q et al (2012) | | | Zeng W et al (2011) | | | |  | | | | | | | | | | | | | |
| Study Objective |  | |  | | |  | | | |  | | | | | | | | | | | | | |
| 1. Is the hypothesis/aim/objective of the study stated clearly in the abstract, introduction, or methods section? | 1 | | 1 | | | 1 | | | |  | | | | | | | | | | | | | |
| Study Population |  | |  | | |  | | | |  | | | | | | | | | | | | | |
| 2. Are the characteristics of the participants included in the study described? | 1 | | 1 | | | 1 | | | |  | | | | | | | | | | | | | |
| 3. Were the cases collected in more than one centre? | 0 | | 0 | | | 0 | | | |  | | | | | | | | | | | | | |
| 4. Are the eligibility criteria (inclusion and exclusion criteria) for entry into the study explicit and appropriate? | 1 | | 1 | | | 1 | | | |  | | | | | | | | | | | | | |
| 5. Were participants recruited consecutively? | 1 | | 1 | | | 1 | | | |  | | | | | | | | | | | | | |
| 6. Did participants enter the study at a similar point in the disease? | 1 | | 1 | | | 1 | | | |  | | | | | | | | | | | | | |
| Intervention and co-intervention |  | |  | | |  | | | |  | | | | | | | | | | | | | |
| 7. Was the intervention clearly described in the study? | 1 | | 1 | | | 1 | | | |  | | | | | | | | | | | | | |
| 8. Were additional interventions (cointerventions) clearly reported in the study? | 1 | | 1 | | | 1 | | | |  | | | | | | | | | | | | | |
| Outcome measures |  | |  | | |  | | | |  | | | | | | | | | | | | | |
| 9. Are the outcome measures clearly defined in the introduction or methods section? | 1 | | 1 | | | 0 | | | |  | | | | | | | | | | | | | |
| 10. Were relevant outcomes appropriately measured with objective and/or subjective methods? | 1 | | 1 | | | 1 | | | |  | | | | | | | | | | | | | |
| 11. Were outcomes measured before and after intervention? | 1 | | 1 | | | 1 | | | |  | | | | | | | | | | | | | |
| Statistical analysis |  | |  | | |  | | | |  | | | | | | | | | | | | | |
| 12. Were the statistical tests used to assess the relevant outcomes appropriate? | 1 | | 0 | | | 0 | | | |  | | | | | | | | | | | | | |
| Results and conclusions |  | |  | | |  | | | |  | | | | | | | | | | | | | |
| 13. Was the length of follow-up reported? | 1 | | 1 | | | 1 | | | |  | | | | | | | | | | | | | |
| 14. Was the loss to follow-up reported? | 1 | | 0 | | | 1 | | | |  | | | | | | | | | | | | | |
| 15. Does the study provide estimates of the random variability in the data analysis of relevant outcomes? | 1 | | 1 | | | 0 | | | |  | | | | | | | | | | | | | |
| 16. Are adverse events reported? | 1 | | 1 | | | 1 | | | |  | | | | | | | | | | | | | |
| 17. Are the conclusions of the study supported by results? | 1 | | 1 | | | 1 | | | |  | | | | | | | | | | | | | |
| Competing interests and sources of support |  | |  | | |  | | | |  | | | | | | | | | | | | | |
| 18. Are both competing interests and sources of support for the study reported? | 0 | | 0 | | | 0 | | | |  | | | | | | | | | | | | | |
| Total score | 16 | | 14 | | | 13 | | | |  | | | | | | | | | | | | | |

a The number of yes responses is counted for each item and the total score is 18.

Supplemental tab E. Secondary Results of Clinical Index, Intra- and Post-operative Complications

| Outcomes |  | Study type | Included study NO. | I2 | Effect size (95%CI) | P value | Egger’ test for publication bias (p-value) |
| --- | --- | --- | --- | --- | --- | --- | --- |
| Other results of clinical index | Duration of procedure (min) | RCT | 4 | 97 | SMD = -2.86 (-4.01, -1.71) | <0.00001 | 0.127 |
|  |  | PCS | 6 | 98 | SMD = -2.90 (-3.90, -1.89) | <0.00001 |  |
|  |  | RCC | 8 | 96 | SMD = -2.38 (-3.09, -1.67) | <0.00001 |  |
|  | Intensive care units stay (h) | RCT | 2 | 95 | SMD = -2.07 (-3.21, -0.92) | 0.0004 | 0.547 |
|  |  | PCS | 3 | 97 | SMD = -1.12 (-2.28, 0.04) | 0.06 |  |
|  |  | RCC | 8 | 99 | SMD = -1.91 (-3.25, -0.57) | 0.005 |  |
|  | Hospital stay (d) | RCT | 2 | 0 | SMD = -0.80 (-1.01, -0.59) | <0.00001 | 0.239 |
|  |  | PCS | 4 | 34 | SMD = -1.68 (-1.88, -1.48) | <0.00001 |  |
|  |  | RCC | 7 | 98 | SMD = -1.92 (-2.74, -1.11) | <0.00001 |  |
|  | Transfusion patient No. | RCT | 1 | 0 | RR = 0.04 (0.01, 0.11) | <0.00001 | 0.137 |
|  |  | PCS | 5 | 94 | RR = 0.11 (0.03, 0.37) | 0.0003 |  |
|  |  | RCC | 7 | 94 | OR = 0.00 (0.00, 0.10) | 0.001 |  |
|  | Total cost (RMB) | RCT | 2 | 100 | SMD = -1.59 (-4.54, 1.36) | 0.29 | 0.627 |
|  |  | PCS | 5 | 99 | SMD = -0.43 (-1.48, 0.62) | 0.42 |  |
|  |  | RCC | 5 | 99 | SMD = 3.92 (1.35, 6.49) | 0.003 |  |
| Intra-operative arrhythmia | Severe arrhythmia^a^ | PCS | 2 | 86 | RR = 0.38 (0.04, 3.24) | 0.38 |  |
|  | Mild arrhythmia^b^ | PCS | 2 | 21 | RR = 0.42 (0.31, 0.58) | <0.00001 |  |
|  |  | RCC | 1 | 0 | OR = 1.18 (0.52, 2.67) | 0.68 |  |
| Other intra-operative complications | Left ventricular failure | PCS | 1 | 0 | RR = 0.33 (0.01, 8.04) | 0.5 |  |
|  | hemorrhage | RCC | 1 | 0 | OR = 1.00 (0.04, 27.70) | 1 |  |
| Post-operative arrhythmia | Complete atrioventricular block | PCS | 4 | 3 | RR = 0.55 (0.18, 1.71) | 0.3 |  |
|  |  | RCC | 1 | 0 | OR = 0.70 (0.03, 17.41) | 0.83 |  |
|  | Intermittent complete atrioventricular block | RCT | 1 | 0 | RR = 0.20 (0.01, 4.15) | 0.3 |  |
|  | Mobitz type II atrioventricular block | PCS | 2 | 0 | RR = 1.23 (0.18, 8.23) | 0.83 |  |
|  |  | RCC | 1 | 0 | OR = 0.98 (0.04, 24.35) | 0.99 |  |
|  | Mobitz type I atrioventricular block | PCS | 1 | 0 | RR = 3.28 (0.16, 67.07) | 0.44 |  |
|  |  | RCC | 1 | 0 | OR = 0.11 (0.01, 2.22) | 0.15 |  |
|  | First degree atrioventricular block | RCT | 1 | 0 | RR = 17.00 (0.99, 293.04) | 0.05 |  |
|  |  | RCC | 1 | 0 | OR = 0.84 (0.07, 9.48) | 0.89 |  |
|  | Complete right bundle branch block | RCT | 1 | 0 | RR = 0.05 (0.02, 0.15) | <0.00001 |  |
|  |  | RCC | 2 | 57 | OR = 0.90 (0.04, 23.00) | 0.95 |  |
|  | Incomplete right bundle branch block | RCT | 2 | 0 | RR = 0.31 (0.19, 0.53) | <0.0001 |  |
|  |  | PCS | 1 | 0 | RR = 0.45 (0.30, 0.67) | <0.0001 |  |
|  |  | RCC | 6 | 0 | OR = 0.81 (0.50, 1.29) | 0.36 |  |
|  | Incomplete left bundle branch block | RCT | 1 | 0 | RR = 1.50 (0.25, 8.90) | 0.66 |  |
|  |  | PCS | 2 | 0 | RR = 0.93 (0.21, 4.25) | 0.93 |  |
|  |  | RCC | 2 | 0 | OR = 3.28 (0.41, 26.28) | 0.26 |  |
|  | Incomplete auriculoventricular dissociation | RCC | 1 | 0 | OR = 0.33 (0.02, 6.97) | 0.48 |  |
|  | Paroxysmal atrial fibrillation | PCS | 1 | 0 | RR = 0.13 (0.02, 1.09) | 0.06 |  |
|  |  | RCC | 1 | 0 | OR = 0.23 (0.02, 2.07) | 0.19 |  |
|  | Atrial flutter | RCC | 1 | 0 | OR = 0.55 (0.02, 13.83) | 0.72 |  |
|  | Atrial tachycardia | RCC | 1 | 0 | OR = 0.55 (0.02, 13.83) | 0.72 |  |
|  | Atrial premature beats | PCS | 1 | 0 | RR = 1.09 (0.16, 7.57) | 0.93 |  |
|  |  | RCC | 2 | 0 | OR = 0.85 (0.09, 8.33) | 0.89 |  |
|  | Transient sinus or supraventricular arrhythmia | RCC | 2 | 0 | OR = 3.37 (1.29, 8.81) | 0.01 |  |
|  | Junctional escape rhythm | RCC | 1 | 0 | OR = 0.18 (0.01, 3.88) | 0.28 |  |
|  | Junctional ectopic tachycardia | RCT | 1 | 0 | RR = 0.33 (0.01, 8.15) | 0.5 |  |
|  |  | RCC | 1 | 0 | OR = 0.33 (0.02, 6.97) | 0.48 |  |
|  | Short paroxysmal ventricular tachycardia | RCC | 1 | 0 | OR = 1.64 (0.57, 4.76) | 0.36 |  |
|  | Premature ventricular contraction | RCC | 4 | 0 | OR = 1.04 (0.26, 4.16) | 0.96 |  |
| Post-operative valvular regurgitation | Tricuspid regurgitation | RCT | 1 | 0 | RR = 2.45 (1.50, 4.00) | 0.0004 |  |
|  |  | PCS | 4 | 74 | RR = 0.60 (0.33, 1.09) | 0.09 |  |
|  |  | RCC | 8 | 39 | OR = 0.50 (0.31, 0.82) | 0.006 |  |
|  | Aortic regurgitation | RCT | 2 | 0 | RR = 0.80 (0.39, 1.66) | 0.55 |  |
|  |  | PCS | 2 | 54 | RR = 0.21 (0.04, 1.02) | 0.05 |  |
|  |  | RCC | 7 | 11 | OR = 1.59 (0.98, 2.57) | 0.06 |  |
|  | Mitral regurgitation | RCT | 1 | 0 | RR = 0.89 (0.46, 1.71) | 0.72 |  |
|  |  | RCC | 4 | 45 | OR = 1.43 (0.49, 4.13) | 0.51 |  |
|  | Pulmonary regurgitation | RCC | 3 | 0 | OR = 0.67 (0.20, 2.22) | 0.52 |  |
| Other post-operative complication | Incision infection | PCS | 2 | 0 | RR = 0.28 (0.11, 0.70) | 0.007 |  |
|  |  | RCC | 5 | 0 | OR = 0.31 (0.09, 1.08) | 0.07 |  |
|  | Right ventricular outflow tract stenosis | PCS | 1 | 0 | RR = 2.03 (0.08, 49.44) | 0.66 |  |
|  | Left ventricular dysfunction | RCT | 1 | 0 | RR = 0.03 (0.00, 0.47) | 0.01 |  |
|  |  | RCC | 3 | 0 | OR = 0.30 (0.05, 1.94) | 0.21 |  |
|  | Pericardial effusion or pleural effusion | RCT | 2 | 27 | RR = 0.63 (0.11, 3.82) | 0.62 |  |
|  |  | PCS | 5 | 61 | RR = 0.35 (0.07, 1.75) | 0.2 |  |
|  |  | RCC | 9 | 25 | OR = 0.35 (0.20, 0.62) | 0.0004 |  |
|  | Pneumonia | RCT | 1 | 0 | RR = 0.67 (0.12, 3.64) | 0.64 |  |
|  |  | PCS | 2 | 0 | RR = 0.05 (0.01, 0.38) | 0.003 |  |
|  |  | RCC | 5 | 0 | OR = 0.19 (0.09, 0.41) | <0.0001 |  |
|  | Pneumothorax | PCS | 1 | 0 | RR = 0.23 (0.01, 5.49) | 0.36 |  |
|  |  | RCC | 1 | 0 | OR = 0.87 (0.04, 18.78) | 0.93 |  |
|  | Subcutaneous emphysema | RCT | 1 | 0 | RR = 0.33 (0.01, 7.80) | 0.49 |  |
|  |  | RCC | 1 | 0 | OR = 0.62 (0.03, 12.36) | 0.75 |  |
|  | Atelectasis | PCS | 1 | 0 | RR = 0.23 (0.01, 5.49) | 0.36 |  |
|  |  | RCC | 1 | 0 | OR = 0.13 (0.01, 2.65) | 0.19 |  |
|  | Peripheral vascular injury | PCS | 1 | 0 | RR = 0.25 (0.06, 1.13) | 0.07 |  |
|  | Thromboembolism | PCS | 1 | 0 | RR = 3.00 (0.12, 72.35) | 0.5 |  |
|  | Pericardial tamponade | RCC | 2 | 0 | OR = 2.93 (0.35, 24.38) | 0.32 |  |
|  | Low output syndrome | RCC | 2 | 9 | OR = 0.20 (0.02, 1.75) | 0.15 |  |
|  | Acute respiratory distress syndrome or acute lung injury | RCC | 2 | 56 | OR = 0.17 (0.01, 4.09) | 0.27 |  |
|  | Abnormal bleeding | RCC | 1 | 0 | OR = 0.32 (0.02, 5.93) | 0.45 |  |
|  | Hemolysis | RCC | 1 | 0 | OR = 0.98 (0.04, 24.35) | 0.99 |  |
|  | Pnepmopericardium | RCC | 1 | 0 | OR = 0.33 (0.01, 8.22) | 0.5 |  |
|  | Infectious endocarditis | RCC | 1 | 0 | OR = 0.50 (0.02, 12.66) | 0.67 |  |
|  | Death | RCC | 2 | 0 | OR = 0.74 (0.12, 4.48) | 0.75 |  |

a Severe arrhythmia refer to the rhythm disorder which can cause severe hemodynamic disorder, temporary syncope, or sudden death, including transient or persistent complete atrioventricular block(AVB), Mobitz type II AVB, ventricular tachycardia.

b Mild arrhythmia is the well-tolerated rhythm disorder, including short and well-tolerated episode of junctional rhythm, new-onset incomplete or complete right bundle branch block, temporary sinus bradycardia, atrial premature beats, transient left bundle branch block.

Supplemental tab F. Sensitivity Analyses of Complications, Conversion, and Reoperation

|  | ES | [95% Conf. Interval] | p | I-squared | p | Q |
| --- | --- | --- | --- | --- | --- | --- |
| cAVB |  |  |  |  |  |  |
| Fixed model | 0.107 | (0.091, 0.123) | 0.000 | 0.0% | 1.000 | 106.22 |
| Random model | 0.107 | (0.091, 0.123) | 0.000 | 0.0% | 1.000 | 106.22 |
| Influence analysis | No sig. influence |  |  |  |  |  |
| Trim and fill | fixed |  |  |  |  |  |
| Fixed model | 1.084 | (1.068, 1.101) | 0.000 | 0.0% | 0.933 | 209.965 |
| Random model | 1.084 | (1.068, 1.101) | 0.000 | 0.0% | 0.933 | 209.965 |
| Publication bias (Egger) |  |  | 0.000 |  |  |  |
| RS |  |  |  |  |  |  |
| Fixed model | 0.252 | (0.236, 0.268) | 0.000 | 81.3% | 0.000 | 875.60 |
| Random model | 0.309 | (0.266, 0.352) | 0.000 | 81.3% | 0.000 | 875.60 |
| Influence analysis | No sig. influence |  |  |  |  |  |
| Trim and fill | random |  |  |  |  |  |
| Fixed model | 1.261 | (1.241, 1.281) | 0.000 | 84.1% | 0.000 | 1138.478 |
| Random model | 1.304 | (1.245, 1.366) | 0.000 | 84.1% | 0.000 | 1138.478 |
| Publication bias (Egger) |  |  | 0.001 |  |  |  |
| TR |  |  |  |  |  |  |
| Fixed model | 0.216% | (0.200, 0.232) | 0.000 | 80.6% | 0.000 | 842.36 |
| Random model | 0.261% | (0.218, 0.303) | 0.000 | 80.6% | 0.000 | 842.36 |
| Influence analysis | No sig. influence |  |  |  |  |  |
| Trim and fill | random |  |  |  |  |  |
| Fixed model | 1.241 | (1.222, 1.261) | 0.000 | 80.6% | 0.000 | 842.364 |
| Random model | 1.298 | (1.244, 1.354) | 0.000 | 80.6% | 0.000 | 842.364 |
| Publication bias (Egger) |  |  | 0.012 |  |  |  |
| AR |  |  |  |  |  |  |
| Fixed model | 0.125% | (0.109, 0.141) | 0.000 | 58.0% | 0.000 | 388.06 |
| Random model | 0.192% | (0.162, 0.222) | 0.000 | 58.0% | 0.000 | 388.06 |
| Influence analysis | No sig. influence |  |  |  |  |  |
| Trim and fill | random |  |  |  |  |  |
| Fixed model | 1.075% | (1.059, 1.091) | 0.000 | 71.3% | 0.000 | 840.298 |
| Random model | 1.094% | (1.058, 1.132) | 0.000 | 71.3% | 0.000 | 840.298 |
| Publication bias (Egger) |  |  | 0.000 |  |  |  |

Post-operative complications

Conversion to COHS:

|  | ES | [95% Conf. Interval] | p | I-squared | p | Q |
| --- | --- | --- | --- | --- | --- | --- |
| Conversion |  |  |  |  |  |  |
| Fixed model | 0.401 | (0.385, 0.418) | 0.000 | 59.6% | 0.000 | 395.91 |
| Random model | 0.400 | (0.369, 0.431) | 0.000 | 59.6% | 0.000 | 395.91 |
| Influence analysis | No sig. influence |  |  |  |  |  |
| Trim and fill | fixed | exponentialform |  |  |  |  |
| Fixed model | 1.477 | ( 1.453, 1.501) | 0.000 | 64.7% | 0.000 | 493.558 |
| Random model | 1.455 | ( 1.408, 1.504) | 0.000 | 64.7% | 0.000 | 493.558 |
| Publication bias (Egger) |  |  | 0.417 |  |  |  |
| RS |  |  |  |  |  |  |
| Fixed model | 0.217 | ( 0.198, 0.236) | 0.000 | 11.3% | 0.139 | 166.78 |
| Random model | 0.213 | (0.191, 0.235) | 0.000 | 11.3% | 0.139 | 166.78 |
| Influence analysis | No sig. influence |  |  |  |  |  |
| Trim and fill | fixed | exponentialform |  |  |  |  |
| Fixed model | 1.219 | (1.197, 1.242) | 0.000 | 27.3% | 0.001 | 246.232 |
| Random model | 1.206 | (1.177, 1.237) | 0.000 | 27.3% | 0.001 | 246.232 |
| Publication bias (Egger) |  |  | 0.010 |  |  |  |
| OFO |  |  |  |  |  |  |
| Fixed model | 0.133 | ( 0.115, 0.152) | 0.000 | 0.0% | 0.963 | 118.79 |
| Random model | 0.133 | (0.115, 0.152) | 0.000 | 0.0% | 0.963 | 118.79 |
| Influence analysis | No sig. influence |  |  |  |  |  |
| Trim and fill | fixed | exponentialform |  |  |  |  |
| Fixed model | 1.109 | (1.090, 1.129) | 0.000 | 6.1% | 0.244 | 227.949 |
| Random model | 1.112 | (1.057, 1.122) | 0.000 | 6.1% | 0.244 | 227.949 |
| Publication bias (Egger) |  |  | 0.000 |  |  |  |
| TR |  |  |  |  |  |  |
| Fixed model | 0.120 | ( 0.101, 0.138) | 0.000 | 0.0% | 1.000 | 86.89 |
| Random model | 0.120 | (0.101, 0.138) | 0.000 | 0.0% | 1.000 | 86.89 |
| Influence analysis | No sig. influence |  |  |  |  |  |
| Trim and fill | fixed | exponentialform |  |  |  |  |
| Fixed model | 1.096 | (1.077, 1.115) | 0.000 | 0.0% | 0.983 | 176.134 |
| Random model | 1.096 | (1.077, 1.115) | 0.000 | 0.0% | 0.983 | 176.134 |
| Publication bias (Egger) |  |  | 0.000 |  |  |  |
| AR |  |  |  |  |  |  |
| Fixed model | 0.187 | ( 0.168, 0.206) | 0.000 | 17.7% | 0.039 | 179.78 |
| Random model | 0.205 | (0.181, 0.228) | 0.000 | 17.7% | 0.039 | 179.78 |
| Influence analysis | No sig. influence |  |  |  |  |  |
| Trim and fill | fixed |  |  |  |  |  |
| Fixed model | 1.145 | (1.125, 1.165) | 0.000 | 44.5% | 0.000 | 376.551 |
| Random model | 1.154 | (1.122, 1.186) | 0.000 | 44.5% | 0.000 | 376.551 |
| Publication bias (Egger) |  |  | 0.000 |  |  |  |
| FTER |  |  |  |  |  |  |
| Fixed model | 0.173 | ( 0.154, 0.192) | 0.000 | 0.0% | 0.921 | 124.45 |
| Random model | 0.173 | ( 0.154, 0.192) | 0.000 | 0.0% | 0.921 | 124.45 |
| Influence analysis | No sig. influence |  |  |  |  |  |
| Trim and fill | fixed |  |  |  |  |  |
| Fixed model | 1.158 | (1.137, 1.179) | 0.000 | 8.5% | 0.174 | 218.639 |
| Random model | 1.155 | (1.132, 1.179) | 0.000 | 8.5% | 0.174 | 218.639 |
| Publication bias (Egger) |  |  | 0.000 |  |  |  |
| SA |  |  |  |  |  |  |
| Fixed model | 0.134 | ( 0.115, 0.153) | 0.000 | 0.0% | 1.000 | 74.61 |
| Random model | 0.134 | ( 0.115, 0.153) | 0.000 | 0.0% | 1.000 | 74.61 |
| Influence analysis | No sig. influence |  |  |  |  |  |
| Trim and fill | fixed |  |  |  |  |  |
| Fixed model | 1.113 | (1.094, 1.133) | 0.000 | 0.0% | 1.000 | 147.044 |
| Random model | 1.113 | (1.094, 1.133) | 0.000 | 0.0% | 1.000 | 147.044 |
| Publication bias (Egger) |  |  | 0.000 |  |  |  |

FTER: failure to establish the occluder conveying rail, OFO: occluder fall off, SA: severe arrhythmia

Reoperation (conventional open-heart surgery):

|  | ES | [95% Conf. Interval] | p | I-squared | p | Q |
| --- | --- | --- | --- | --- | --- | --- |
| Reoperation |  |  |  |  |  |  |
| Fixed model | 0.120 | (0.104, 0.136) | 0.000 | 0.0% | 0.972 | 133.10 |
| Random model | 0.120 | (0.104, 0.136) | 0.000 | 0.0% | 0.972 | 133.10 |
| Influence analysis | No sig. influence |  |  |  |  |  |
| Trim and fill | fixed |  |  |  |  |  |
| Fixed model | 0.090 | (0.075, 0.105) | 0.000 | 10.0% | 0.112 | 271.199 |
| Random model | 0.095 | (0.077, 0.112) | 0.000 | 10.0% | 0.112 | 271.199 |
| Publication bias (Egger) |  |  | 0.000 |  |  |  |
| DOTO |  |  |  |  |  |  |
| Fixed model | 0.099 | (0.083, 0.115) | 0.000 | 0.0% | 1.000 | 85.71 |
| Random model | 0.099 | (0.083, 0.115) | 0.000 | 0.0% | 1.000 | 85.71 |
| Influence analysis | No sig. influence |  |  |  |  |  |
| Trim and fill | fixed |  |  |  |  |  |
| Fixed model | 0.075 | (0.060, 0.090) | 0.000 | 0.0% | 1.000 | 169.941 |
| Random model | 0.075 | (0.060, 0.090) | 0.000 | 0.0% | 1.000 | 169.941 |
| Publication bias (Egger) |  |  | 0.000 |  |  |  |
| AR |  |  |  |  |  |  |
| Fixed model | 0.092 | (0.076, 0.109) | 0.000 | 0.0% | 1.000 | 78.67 |
| Random model | 0.092 | (0.076, 0.109) | 0.000 | 0.0% | 1.000 | 78.67 |
| Influence analysis | No sig. influence |  |  |  |  |  |
| Trim and fill | fixed |  |  |  |  |  |
| Fixed model | 0.074 | (0.059, 0.090) | 0.000 | 0.0% | 1.000 | 149.968 |
| Random model | 0.074 | (0.059, 0.090) | 0.000 | 0.0% | 1.000 | 149.968 |
| Publication bias (Egger) |  |  | 0.000 |  |  |  |
| cAVB |  |  |  |  |  |  |
| Fixed model | 0.103 | (0.086, 0.119) | 0.000 | 0.0% | 1.000 | 62.39 |
| Random model | 0.103 | (0.086, 0.119) | 0.000 | 0.0% | 1.000 | 62.39 |
| Influence analysis | No sig. influence |  |  |  |  |  |
| Trim and fill | fixed |  |  |  |  |  |
| Fixed model | 0.086 | (0.071, 0.102) | 0.000 | 0.0% | 1.000 | 113.678 |
| Random model | 0.086 | (0.071, 0.102) | 0.000 | 0.0% | 1.000 | 113.678 |
| Publication bias (Egger) |  |  | 0.000 |  |  |  |
| RS |  |  |  |  |  |  |
| Fixed model | 0.092 | (0.076, 0.109) | 0.000 | 0.0% | 1.000 | 72.07 |
| Random model | 0.092 | (0.076, 0.109) | 0.000 | 0.0% | 1.000 | 72.07 |
| Influence analysis | No sig. influence |  |  |  |  |  |
| Trim and fill | fixed |  |  |  |  |  |
| Fixed model | 0.075 | (0.059, 0.090) | 0.000 | 0.0% | 1.000 | 136.173 |
| Random model | 0.075 | (0.059, 0.090) | 0.000 | 0.0% | 1.000 | 136.173 |
| Publication bias (Egger) |  |  | 0.000 |  |  |  |

DOTO: dropout of the occluder

Supplemental tab G. Relevant factors of Complications, Conversion and Reoperation

| Outcomes | Middle approach | Left approach | Right approach | mVSD | pmVSD | dcsVSD | other | VSD size | age | gender | weight | Occluder size |
| --- | --- | --- | --- | --- | --- | --- | --- | --- | --- | --- | --- | --- |
| Post-operative complications |  |  |  |  |  |  |  |  |  |  |  |  |
| Complete atrioventricular block | n.s. | n.s. | n.s. | 0.002 | 0.009 | n.s. | n.s. | 0.013 | n.s. | 0.023 | n.s. | 0.006 |
| Residual shunt | n.s. | n.s. | n.s. | 0.000 | 0.000 | n.s. | n.s. | n.s. | n.s. | n.s. | n.s. | 0.028 |
| Tricuspid regurgitation | n.s. | n.s. | n.s. | n.s. | n.s. | n.s. | n.s. | n.s. | n.s. | n.s. | n.s. | n.s. |
| Aortic regurgitation | n.s. | 0.033 | n.s. | n.s. | 0.000 | 0.000 | n.s. | n.s. | n.s. | n.s. | n.s. | n.s. |
| Conversion from TTDC to COHS | n.s. | n.s. | n.s. | n.s. | 0.003 | 0.048 | 0.022 | n.s. | n.s. | n.s. | n.s. | 0.001 |
| Reasons for Conversion |  |  |  |  |  |  |  |  |  |  |  |  |
| Residual shunt | n.s. | n.s. | n.s. | n.s. | n.s. | n.s. | 0.006 | 0.025 | n.s. | n.s. | n.s. | 0.001 |
| Aortic regurgitation | 0.012 | 0.001 | n.s. | n.s. | 0.000 | 0.001 | n.s. | n.s. | n.s. | n.s. | n.s. | 0.039* |
| Failure to establish the rail | n.s. | n.s. | n.s. | 0.032 | n.s. | n.s. | n.s. | n.s. | n.s. | n.s. | n.s. | 0.032 |
| Severe arrhythmia | n.s. | n.s. | n.s. | n.s. | 0.014 | n.s. | n.s. | 0.013 | n.s. | n.s. | n.s. | n.s. |
| Occluder fall off | n.s. | n.s. | n.s. | 0.001 | 0.000* | 0.005 | n.s. | n.s. | n.s. | n.s. | n.s. | 0.032 |
| Tricuspid regurgitation | n.s. | n.s. | n.s. | 0.017 | 0.003 | n.s. | n.s. | 0.001 | n.s. | n.s. | n.s. | 0.002 |
| Open-heart reoperation | n.s. | n.s. | n.s. | 0.001 | 0.000* | 0.008 | n.s. | n.s. | n.s. | n.s. | n.s. | 0.021 |
| Reasons for Conversion |  |  |  |  |  |  |  |  |  |  |  |  |
| Dropout of the occluder | n.s. | n.s. | n.s. | 0.001 | 0.000 | n.s. | n.s. | n.s. | n.s. | n.s. | n.s. | 0.042 |
| Aortic regurgitation | n.s. | n.s. | n.s. | 0.003 | 0.000* | 0.002 | n.s. | 0.032 | n.s. | n.s. | n.s. | 0.018 |
| Complete atrioventricular block | n.s. | n.s. | n.s. | 0.001 | 0.001 | n.s. | n.s. | n.s. | n.s. | n.s. | n.s. | 0.017 |
| Residual shunts | n.s. | n.s. | n.s. | 0.001 | 0.000 | 0.029 | n.s. | n.s. | n.s. | n.s. | n.s. | 0.022 |

mVSD, muscular ventricular septal defect. pmVSD, perimembrane ventricular septal defect. dcsVSD, doubly committed subarterial ventricular septal defect. other, the special type of ventricular septal defect. VSD, ventricular septal defect. Gender was calculated as the proportion of female.

n.s., no significant. red, positive correlation. green, negative correlation. *, independent factor. The results was present as p-value.

Supplemental fig A. Risk of bias in randomized controlled trials


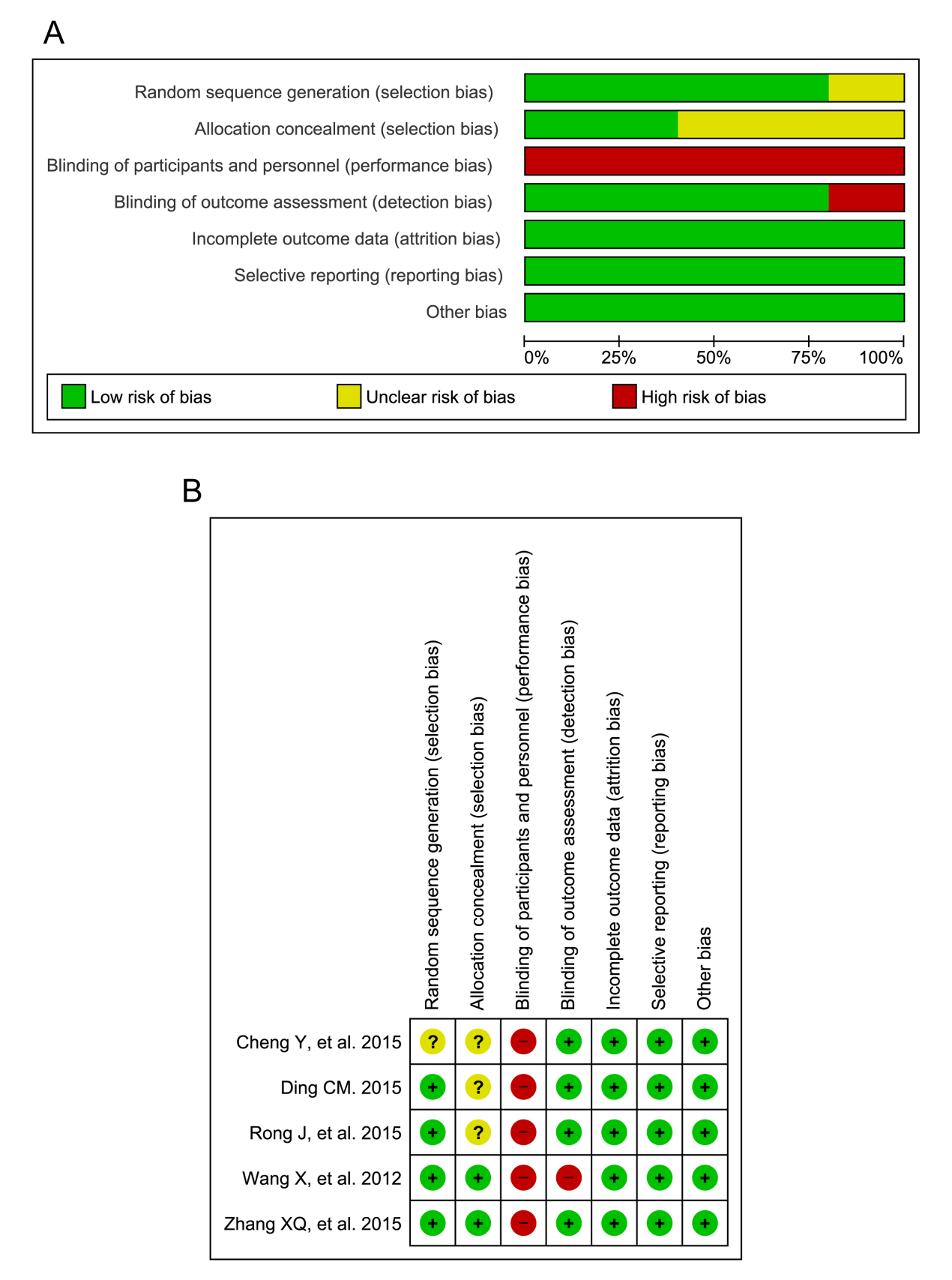


Supplemental fig B. Trial sequential analysis (TSA) of success rate in randomized controlled trials


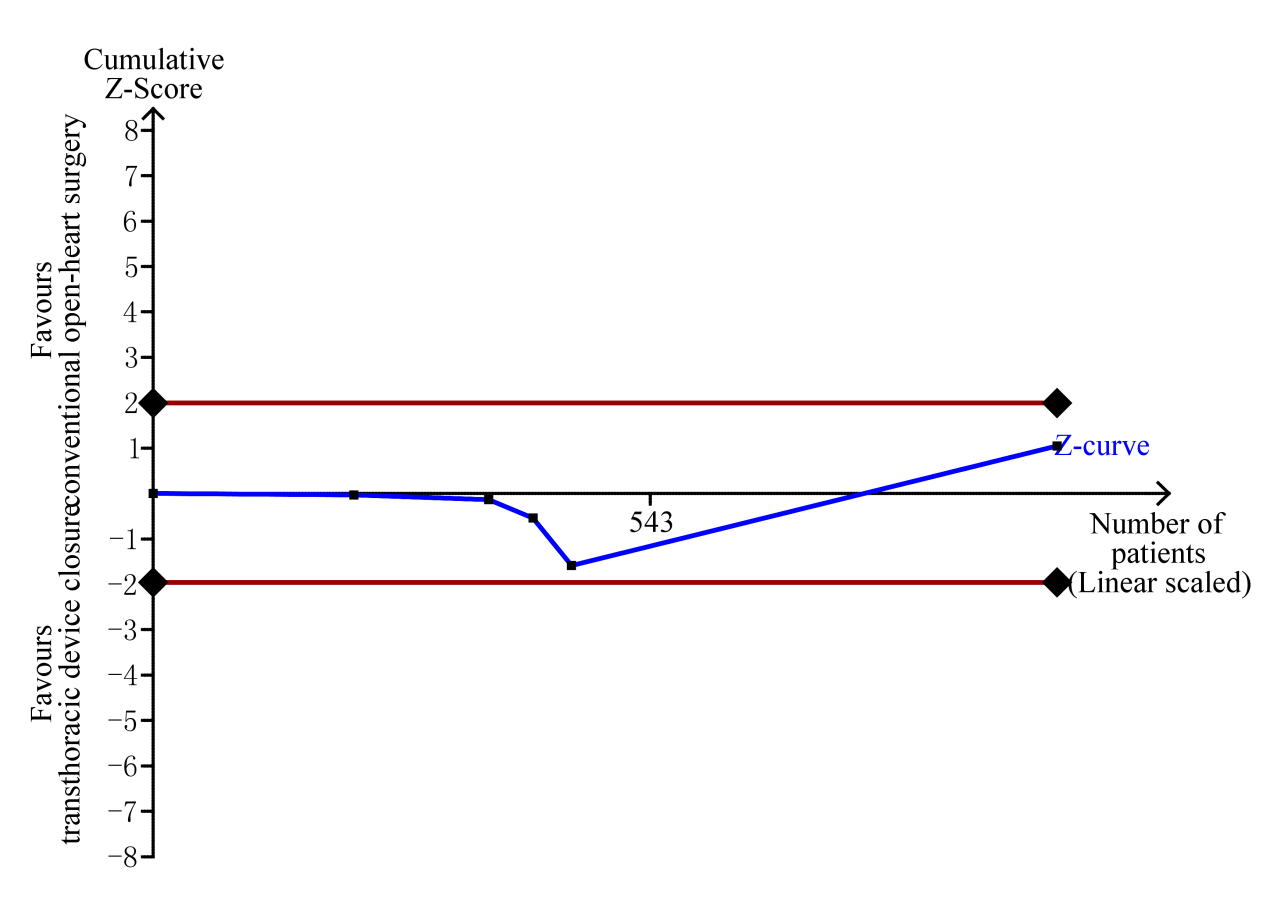

Supplement: Supplementary file 1 — Supplemental materials [file 41598_2017_12500_MOESM1_ESM.doc]
